# Polymeric Dopant-Free Hole Transporting Materials for Perovskite Solar Cells: Structures and Concepts towards Better Performances

**DOI:** 10.3390/polym13101652

**Published:** 2021-05-19

**Authors:** Mohamed M. H. Desoky, Matteo Bonomo, Nadia Barbero, Guido Viscardi, Claudia Barolo, Pierluigi Quagliotto

**Affiliations:** 1 Department of Chemistry and NIS Interdepartmental Center and INSTM Reference Centre, University of Torino, Via Pietro Giuria 7, 10125 Torino, Italy; mohamedmagdyhassan.desoky@unito.it (M.M.H.D.); matteo.bonomo@unito.it (M.B.); nadia.barbero@unito.it (N.B.); guido.viscardi@unito.it (G.V.); claudia.barolo@unito.it (C.B.); 2 ICxT Interdepartmental Centre, Università degli Studi di Torino, Via Lungo Dora Siena 100, 10153 Torino, Italy

**Keywords:** hole transporting materials, dopant-free polymers, organic materials, perovskite solar cells

## Abstract

Perovskite solar cells are a hot topic of photovoltaic research, reaching, in few years, an impressive efficiency (25.5%), but their long-term stability still needs to be addressed for industrial production. One of the most sizeable reasons for instability is the doping of the Hole Transporting Material (HTM), being the salt commonly employed as a vector bringing moisture in contact with perovskite film and destroying it. With this respect, the research focused on new and stable “dopant-free” HTMs, which are inherently conductive, being able to effectively work without any addition of dopants. Notwithstanding, they show impressive efficiency and stability results. The dopant-free polymers, often made of alternated donor and acceptor cores, have properties, namely the filming ability, the molecular weight tunability, the stacking and packing peculiarities, and high hole mobility in absence of any dopant, that make them very attractive and a real innovation in the field. In this review, we tried our best to collect all the dopant-free polymeric HTMs known so far in the perovskite solar cells field, providing a brief historical introduction, followed by the classification and analysis of the polymeric structures, based on their building blocks, trying to find structure–activity relationships whenever possible. The research is still increasing and a very simple polymer (PFDT–2F–COOH) approaches PCE = 22% while some more complex ones overcome 22%, up to 22.41% (PPY2).

## 1. Introduction

The photovoltaic research field has grown enormously in the last few decades and, after the silicon technology, several other new technologies appeared such as Organic PhotoVoltaic (OPV), [1] Dye-sensitized Solar Cells (DSC) [2] and last but surely not least, the Perovskite Solar Cells (PSC) [3]. This latter showed its efficiency to boost in only the last decade from the initial 9.1% of 2012 [4] to the actual 25.5% of early 2021, [5,6] also thanks to the development of materials for the charge transport, [7] becoming a reliable hot topic. While we will discuss the structure of those cells later, here we can stress that the perovskite layer can efficiently achieve charge separation when hit by sunlight rays, and the migration of charges is very fast in this material [7,8]. Due to this, it is of the utmost importance to extract efficiently the charges from perovskite, to avoid charge recombination [9,10] and to gain high photovoltaic efficiencies. In this context, the Hole Transporting Materials (HTMs) are of crucial importance [7,11]. Although inorganic HTMs have been effectively implemented in PSCs (especially when inverted geometry is considered), [12] most of the HTMs have organic nature and can be roughly separated into two main classes: (i) Small Molecule [12,13] and (ii) Polymeric HTMs [12,14]. Conductive polymers [15] were the first organic materials that showed the ability to conduct electricity and they were essentially prepared as highly/fully conjugated organic polymers. Since then, the conductive polymers field developed enormously, and they were used in several organic electronic applications such as OLED, OPV, OFET, but not only those [1,16,17]. In the past, organic molecules were known to behave as insulants being their native conductivity of organic materials (molecules and polymers) often minimal, so that they can be induced to enhance their conductivity by the doping process [9,18,19,20]. This roughly means that the organic structures need to be oxidized to become cationic and thus be able to receive electrons on one side leaving the holes to flow in opposite directions. The doping process can be easily accessed with good results, but has also several drawbacks, e.g., the lowered chemical stability of the HTM in presence of tert-butylpyridine (tBP) [21], chemical and photochemical instability [22] and to behave as a vector for moisture and oxygen that could lead to degradation of the perovskite film and, consequently, of the whole devices. Due to this, in the last years, a great effort was made to prepare new conductive molecules and polymers in which the high polarization of the different moieties composing them can enhance the intrinsic conductivity. As a result, it was found that some polymers and molecules can show a decent to high native conductivity that can make them useful for the PSCs application without any doping. These novel materials are now known as dopant-free HTMs. Throughout the present review, the focus will be directed to dopant-free HTM polymers being small molecules already analyzed in a dedicated paper [23]. While conductive polymers were introduced and studied a long time ago, the conductive behavior of these polymers is still a challenge since it is strictly related to the solid-state film organization. The chemical structure is important but also the formation of amorphous or crystalline domains is important, such as in the case of the well-known poly(3-hexylthiophene) (**P3HT**) [24,25]. The way in which the long polymeric molecules pack is of the utmost importance, since the conductivity is normally related to the charge flow along the main conjugated chain and to the hopping among contiguous polymeric chains, which should have tight contact distances among them [9,25,26,27,28]. This means that not only the difference in acceptor/donor character of the moieties is important, but also the contact distance among chains, to help the charge to flow at high rate [29].

The HTM research field covers an enormous number and a variety of structures and different applications and so it is practically unaffordable to cover all aspects. Even reducing the search to the application in PSCs makes this search highly challenging since this field sees a few papers published per day, making it a rapidly growing hot topic. Luckily, the limitation to the dopant-free polymeric HTMs [12,30,31] made this work feasible even if we cannot exclude losing some papers due to the fast increase of the literature about this topic and we apologize for any unintentional omission.

We also tried to discuss the polymers by looking at the donor and acceptor moieties and tried to find, if any, some structure–activity relationships or at least some indication about the effect that every moiety can give to the final HTM. This is essential to evidence the most promising donor and acceptor moieties and to guide chemists to prepare novel generations of polymeric HTMs aiming at high photovoltaic efficiency.

## 2. Perovskite Solar Cells: The Essential

The use of perovskites in the solar cells field appeared for the first time in 2009 on Dye Sensitized Solar Cells (DSSC or DSC), just to substitute the dye as a light harvester, obtaining a modest efficiency (3.9%) [32]. This was mainly due to intrinsic instability of the perovskite towards liquid systems commonly employed as electrolytes in DSC (see below). The first perovskite solar cell was prepared in 2012 as a solid-state cell, with no liquid inside and where the perovskite could show that its light-harvesting properties were consistently better than that shown in DSSC (efficiency 9.1%) [4], and confirmed that a novel structure of the solar cells would have been developed to exploit properly the high potential of perovskites. Up to now, an excellent and potentially competitive certified efficiency as high as 25.5% was obtained [6]. In order to introduce properly the PSC into the context of this review, we will start with a brief introduction on perovskites and their main characteristics, and we will continue with a discussion of the main different structures of PSC. At the end we will introduce the organic HTMs, mainly focusing on the polymeric ones and on the mechanism of doping.

### 2.1. Perovskite Structure and Properties

**Figure 1:**
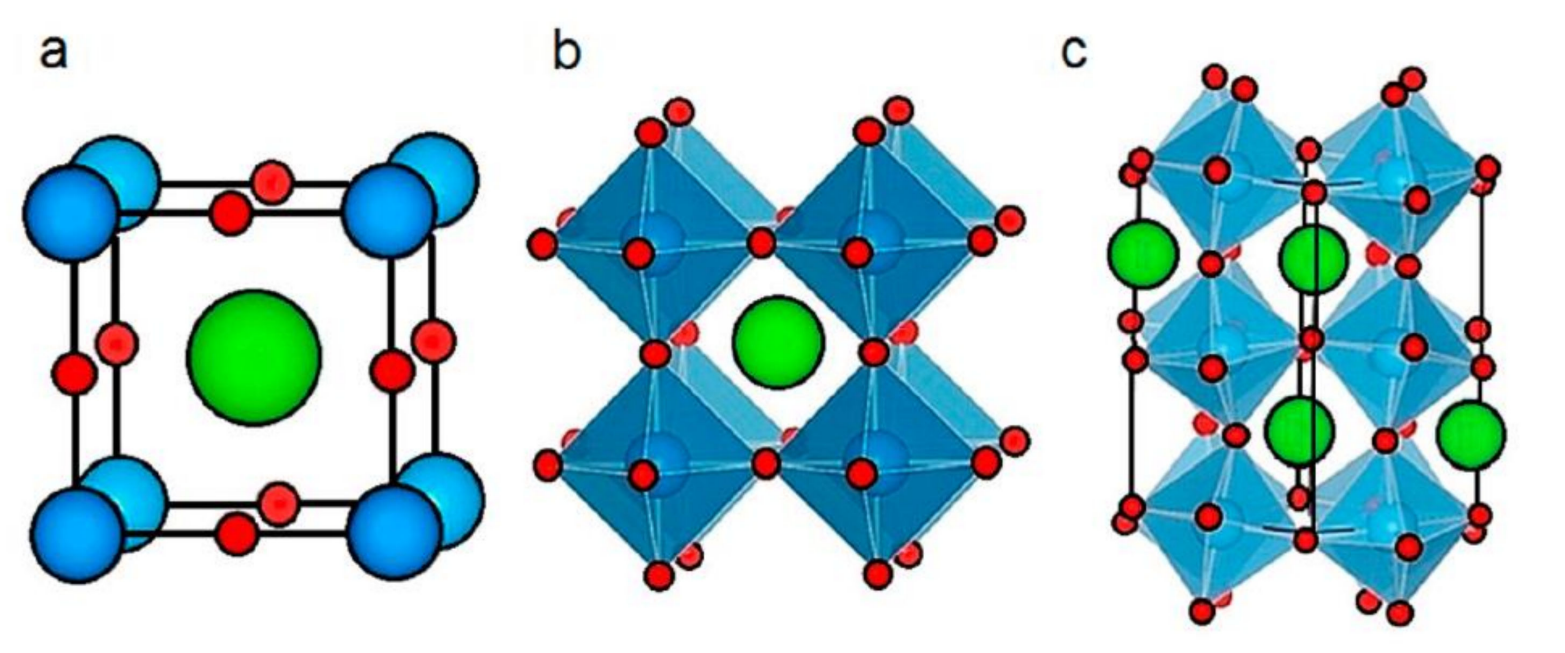
Different visualization of the perovskite structure: (**a**) Crystal structure of organometal trihalide with chemical structure of ABX_3_, where A is the organic cation (green), B the metal cation (blue), and X the halide anion (red); (**b**) perovskite structure showing the B cation assembled around X anions to form BX6 octahedra. (**c**) Tilting of BX_6_ octahedra occurring from nonideal size effects and other factors. Reprinted with permission from (J. Phys. Chem. Lett. 2016, 7, 851–866, ref. [35]) Copyright (2016) American Chemical Society.

Perovskites have a chemical structure represented by the formula ABX_3_. In the perovskite mostly used in the photovoltaic field, A is normally a large volume organic cation (methylammonium (MA), ethylammonium (EA), formamidinium (FA)), B is a small volume inorganic cation, usually consisting of Pb even if also Sn was tried [33]. The Pb is however better protected from oxidation than Sn [34]. giving better stability to the perovskite [35]. The A cation is situated at the corners of the unit cell, X is the halide anion in the six-face center and B is the metal cation placed at the body center (Figure 1) [35].

As a typical example, the most used perovskite is the MAPbI_3_, formed by methylammonium iodide and lead iodide. The typical band-gap (BG) of perovskite used in solar cells is around 1.2–1.6 eV, since it needs to cover almost all the visible range, thus harvesting light at all the possible visible wavelengths. Perovskites of more than 2.0 eV BG can exploit only a minimal fraction of the visible light, while being interesting for applications in which transparency is a mandatory requirement, such as indoor and Building Integrated Photovoltaics (BIPV) [36,37]. The band-gap of these materials is decreasing with (i) an increase of the in-plane M–I–M (M = metal) bond angle (through octahedral distortion), (ii) the increase in the dimensionality of the M(X)_6_ network and (iii) the decrease in the electronegativity of the anions [38]. The band-gap can be tuned by changing the A cation and it increases in the order FAPbI_3_ < MAPbI_3_, < CsPbI_3_. By preparing a proper mixture of FA and MA cations, a “double cation” MA_x_FA_1−x_PbI_3_ perovskite (FAMA) can be obtained whose absorption is red shifted, enhancing the light-harvesting properties. This mixture showed better carrier-collection efficiency, due to their increased diffusion length and reduced charge recombination [39]. The crucial property of the perovskite, in fact, is its carrier diffusion length. In a study comparing the MAPbI_3_ vs. MAPbI_3-x_Cl_x_ it was found that the second one can afford a longer diffusion length for both electrons and holes, reaching even more than 1 mm, one order of magnitude higher than that of MAPbI_3_ [40]. Besides, the FAPbI_3_ shows a larger hole-diffusion length than electron diffusion length with respect to the MAPbI_3_ [41,42]. This also holds in the double cation mixed perovskites, such as MA_x_FA_1−x_PbI_x_ and, in general, holes can travel more than electrons, while the electron and hole diffusion lengths are however longer than the mm scale. These show why it was essential to modify the perovskite structure to improve it by limiting the charge recombination [40]. The determination of bandgap (and energy levels) of perovskite is of utmost importance in the design and choice of a proper HTM; indeed, if the HTM has correctly aligned energy levels to be able to block electron flow from perovskite, even relatively low hole mobility as shown by the organic HTMs is sufficient to ensure a good hole extraction performance [43].

Double cation perovskites such as MA_x_FA_1−x_PbI_3_, however, showed problems in the control of different crystalline phases and, as such, the results obtained can be dependent on the different phase composition. In view of solving this, Saliba et al. incorporated Cs cation into the previous formulations and obtained a stabilization of the black (active) phase for this “triple cation” perovskite (CsFAMA) [44]. Different processes were set up and while the spin coating method is the most used one, this can give problems such as small crystals and many grain boundaries and defects. These facts have important effects on the final device stability, and were efficiently addressed in many ways which are excellently described in a dedicated review [45]. The superior behavior of this kind of perovskite is also witnessed by its use in the cell that achieved the actual PCE record of 25.5% [6].

### 2.2. Deposition Method of Perovskite for PSCs

**Figure 2:**
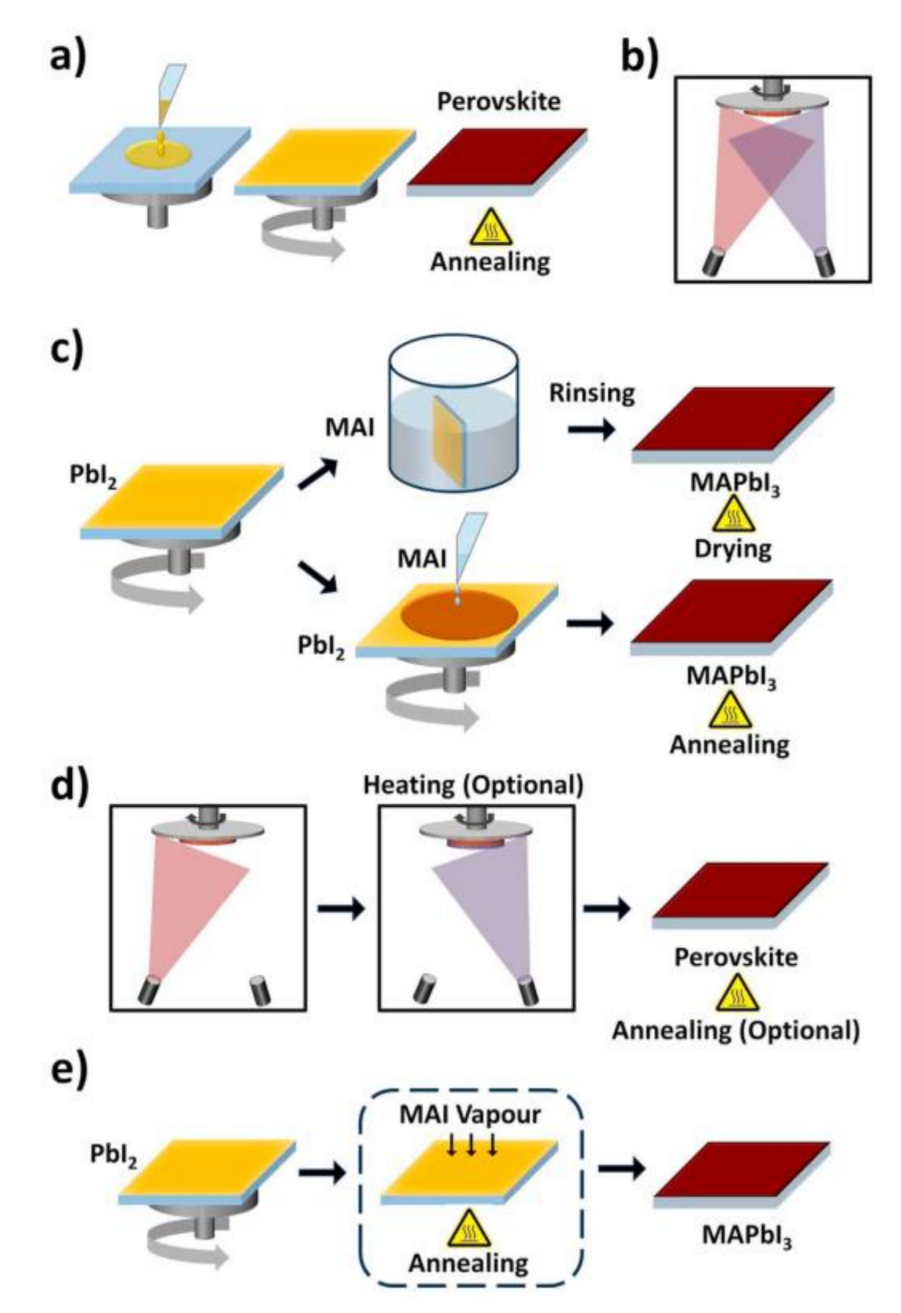
Different perovskite deposition methods (**a**) solution-based one-step method (**b**) co-evaporation; (**c**) solution-based two-step method; (**d**) sequential evaporation; (**e**) vapor-assisted solution process (VASP). Reprinted from Progr. Quantum Electron. 2017, 53, 1–37, Djurišić, A. et al., Perovskite solar cells–An overview of critical issues. [46] Copyright (2017), with permission from Elsevier.

The deposition methods of perovskite to build the PSC (Figure 2) are divided into two classes: (i) solution deposition and (ii) vapor deposition [46]. The solution deposition, on its own, can be divided into two categories: (i) one-step method, and (ii) two-step method. The one-step method is based on the preparation of a solution of all the reactants which is deposited onto the conductive glass and spin-coated. The two-step method requires the deposition of one component, normally the lead halide, followed by further deposition of the second component which will react on the surface, producing the perovskite. While the two-step method was initially preferred to better control the process, in most cases both methods afford excellent results [47]. Starting from this, a lot of modifications can be made by using additives and promoters to control the nucleation and other aspects of the perovskite crystal growth, which is still far from being fully comprehended. These aspects can be found in an excellent dedicated review [46] along with other ways to improve the perovskite film formation, such as the optimization of precursor solvents [48] and antisolvents [49], and additive engineering [50].

Vacuum deposition is an interesting process, which however requires a high vacuum apparatus and the related cost. This method can be performed in two ways: (i) co-deposition, and (ii) sequential deposition. In every case, the instrumentation is complicated by the need to check the thickness rate of the deposition for the co-deposition and of annealing and processes for the sequential deposition. A modified method is the vapor-assisted solution process (VASP) which starts from solutions. The metal halide is deposited first onto the electrode with a solution process and the organic halide is simply evaporated and deposited onto it [51]. Another method used is chemical vapor deposition (CVD), in which the evaporation is thermally assisted [52].

### 2.3. Perovskite Solar Cells—PSCs

Classical PSCs can be conceived as a series of layers of different materials, sandwiched between a TCO (Transparent Conductive Oxide) glass (anode) and a metal electrode (cathode), that can be combined in different ways but that need to be finely tuned in their properties to work properly. Basically, the perovskite combines in itself both the light-harvesting and the charge transport properties, showing excellent diffusion length for both holes and electrons. Yet, these charges are prone to recombine among them. Thus, to avoid charge recombination the perovskite needs to be packed in between two different layers, the ETL (Electron Transporting Layer) which extracts preferentially the electrons and blocks the hole transfer and the HTL (Hole Transporting Layer) which performs in the opposite way, extracting holes efficiently and blocking electrons. This happens in an ideal way, but in practice, it depends on the charge diffusion length of these materials and on their correct alignment of energy levels with the perovskite [53,54].

The disposition of the different layers among the electrodes can give different configurations [55]. The so-called conventional cells show, starting from the bottom, first the conductive glass (or polymeric in flexible PSCs [56]) substrate, then the ETL (an n-type semiconductor, ETM), followed by the perovskite (an intrinsic ambipolar semiconductor) and finally by the HTL (a p-type semiconductor, HTM): these cells are known as belonging to the n-i-p type. In the so-called “inverted” configuration, HTL and ETL are swapped and are referred to as belonging to the p-i-n type [57,58].

In theory, since the perovskite is an ambivalent semiconductor with both fast electrons and holes diffusion, one can think to conceive configuration avoiding the ETL, HTL or even both but this simplification showed limited efficiency results up to now [57].

**Figure 3:**
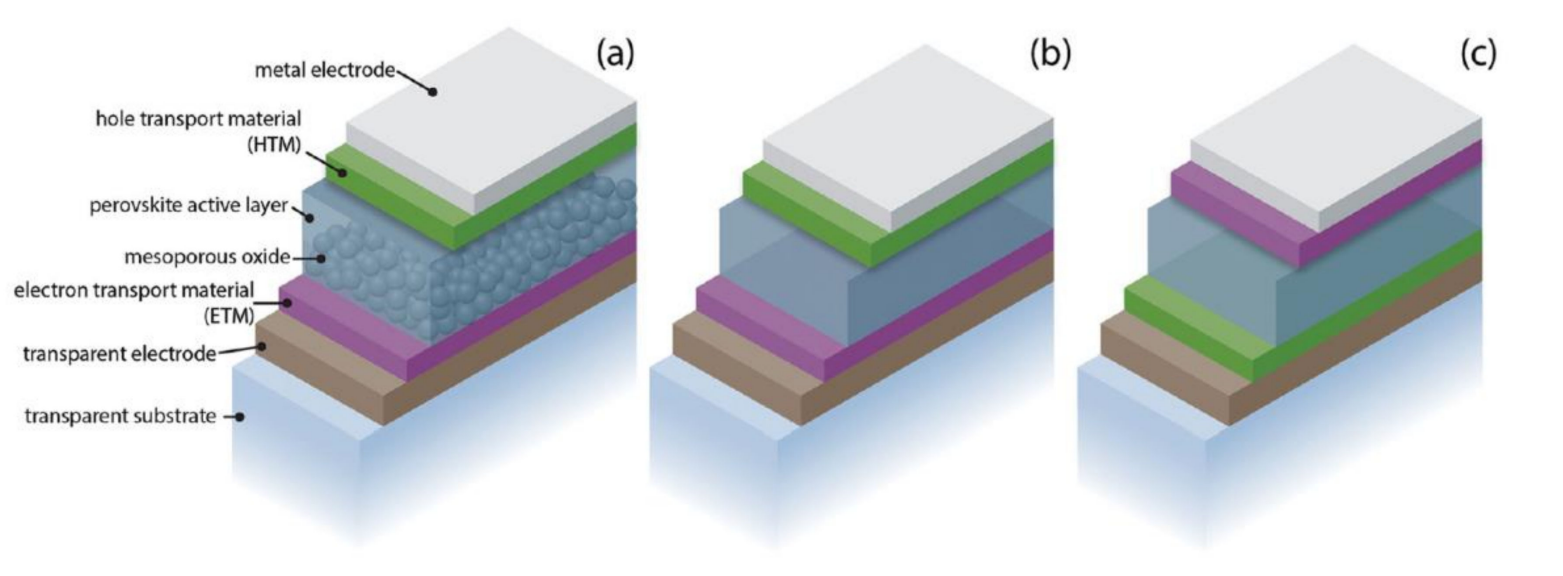
Schematic representation of different PSC device architectures with the (**a**) mesoporous n–i–p configuration, (**b**) planar n–i–p configuration, and (**c**) planar-inverted (p–i–n) device stacks. Reprinted with permission from ref. [55] Copyright 2015 John Wiley and Sons.

From the practical point of view, some main configurations were developed. The first one is derived from the DSCs and is known as the mesoporous configuration (Figure 3a). This requires a conductive glass electrode on which the ETL layer is made of the first compact titania (TiO_2_) layer followed by a second mesoporous titania layer. A further perovskite layer was deposited, which was finally covered by an HTL, which can be made of an organic or inorganic HTM [59,60]. A metal (Ag, Au) electrode is deposited onto the upper HTL.

A second configuration is known as the planar configuration (Figure 3b) and can be conceived as a simpler configuration with respect to the mesoporous one. In practice, the mesoporous titania layer is not present in this configuration while the other elements are still part of the cell and are placed in the same order as for the mesoporous cells. This configuration required a relatively low temperature (<150 °C) for the whole process, opening the doors to the employment of polymeric substrates and leading to flexible PSCs [56].

A third configuration is the inverted one in which, starting from the bottom, the conductive glass is covered by the HTM on which the perovskite is deposited. An ETL is thus layered onto the perovskite, followed by a metal electrode (often Al). The solar cells are referred to as inverted-planar p-i-n (Figure 3c).

## 3. Organic HTMs

### 3.1. Organic HTMs, Doping and Dopant-Free HTMs

As shown before, the topic of this review is focusing on dopant-free organic HTM polymers, since their scientific interest and their application in the last generation of photovoltaic devices [12,30,31]. It was just shown above that the HTMs are a crucial element to build efficient PSCs. The added value of organic HTMs is due to the tunability of organic molecules by synthesis, aiming at meeting the desired requirements for optimized performance. Organic HTMs, both small molecules and polymers, can be tailored towards better performances of photovoltaic devices. [12,30,31] Ideally speaking, they should extract holes and transport them to the metal electrode at the same rate at which they are produced into the perovskite, which is a condition hard to reach. Organic molecules/polymers usually show a limited conductivity and hole mobility which, since the first conductive polymer [15], were sustained by adding doping chemicals. An organic HTM can be considered as a relatively good p-type semiconductor, with hole mobility normally in the range 10^−4^–10^−6^ cm^2^ V^−1^ s^−1^ [7]. Yet, a desirable value for application in PSCs should be higher than 10^−3^ cm^2^ V^−1^ s^−1^. The doping process often increases the hole mobility even of orders of magnitude [53]. The doping phenomenon consists in the introduction of one or more chemicals which can give or extract electrons from the conductive material, so that it becomes negatively or positively charged, i.e., it behaves as an n-doped or a p-doped material, respectively. Here we will give some general info about doping of conductive polymers, leaving the reader to deepen those topics if interested [9,53,61,62].

**Scheme 1:**
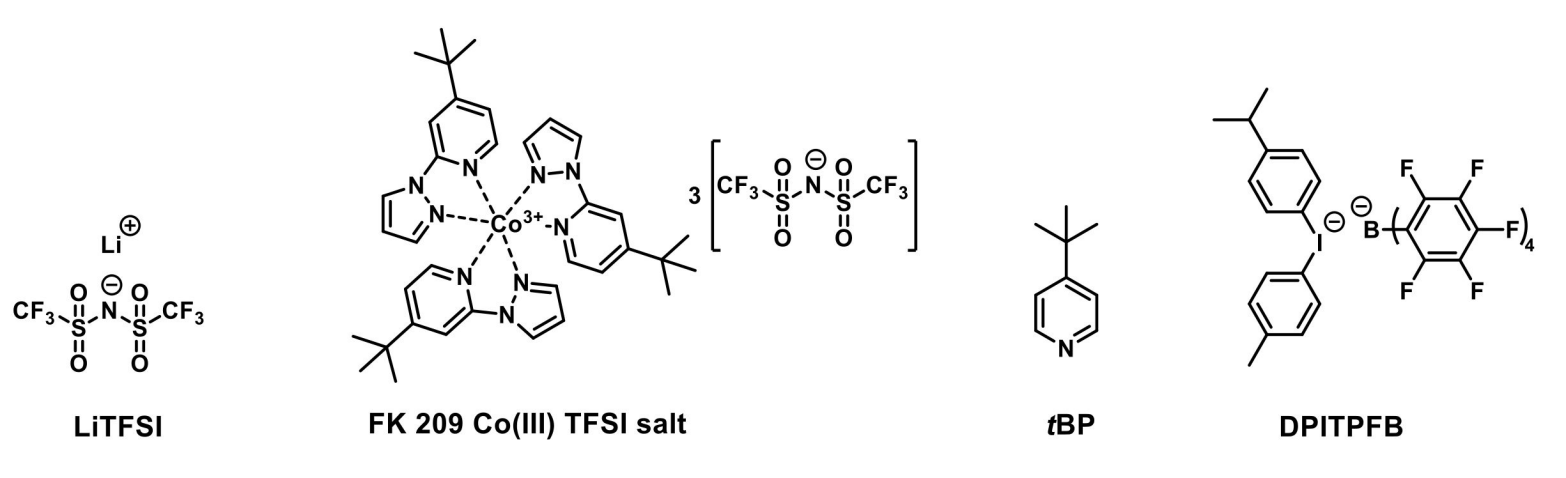
Main dopants/additive used in the doping process of organic Hole Transporting Materials (HTMs).

Usually, the organic HTMs are deposited on perovskite (or different materials for other organic electronic applications) starting from liquid solutions, mainly by spin coating, sometimes by drop-casting techniques mainly for inorganic HTMs [63]. The doping is normally achieved by adding an oxidant compound to the organic HTM (Scheme 1). The quantity of chemicals needed to achieve doping is normally at the level of impurities and by analogy with silicon technology this modification took the name “doping” [9,53]. Since the oxidation process requires that an anion should compensate the positive charge left into the polymer, this anion is usually called “dopant”. The first doping agent for conductive polymers was iodine [15] but now the most known (and used) is the lithium bis(trifluomethylsulfonyl)imide (LiTFSI), which can extract electrons from the organic molecule/polymer. In the last years, also the FK 209 (tris(2-(1H-pyrazol-1-yl)-4-tert-butylpyridine)cobalt(III) tri[hexafluorophosphate]), an organic cobalt complex salt was used as an alternative doping agent. Very recently, the use of AgTFSI was also proposed, to directly oxidize the HTM. Since the silver can be eliminated by precipitation and centrifugation, the HTM salt with TFSI can be produced and used to control the doping into the HTMs mixtures. We will discuss this below in this section [64]. Recently, 4-isopropyl-4′-methyldiphenyliodonium tetrakis(pentafluorophenylborate) (DPITPFB) was proposed as a further dopant agent, since it showed improved hole mobility of a cross-linked polymer of 3–4 orders of magnitude [65].

When the HTM is working as a solid-state film in an electric circuit, it is in between two electrodes that give electrons to the HTM (cathode) and extract electrons from it (anode). In the film, the doped molecule (cation) receives an electron from a closer neutral molecule which takes the positive charge (hole), so while electrons flow in one direction, the holes flow in the opposite direction.

Other additives are often used to improve conductivity and other aspects related to the HTM performance. The tert-butylpyridine (tBP) was used in nearly all doped HTM formulations but its role is still unclear, at least in part. For sure, it can avoid phase segregation between the LiTFSI and the HTM, helping the solubilization of LiTFSI. The tBP improves the smoothness of the HTL film avoiding pinhole formation. It also seems to enhance the conductivity, by acting at the interface with the perovskite, by reducing the trap surface states and charge recombination [66].

However, the additives used in doping formulations are far to be innocent. They decrease substantially the cell performance and stability over a long time, compromising the cell stability. The LiTFSI is problematic since it is a highly hygroscopic salt that induces the absorption of water into the HTM film or can leave pinholes in the film. Humidity can reach the perovskite, leading to the formation of Lead Iodide destroying the perovskite structure [67]. On the other hand, lithium ions are also known to migrate to the ETL layer causing a further decrease in the efficiency and stability of the PSCs [68]. The tBP, on its own, can dissolve the perovskite-making complexes with PbI_2_ and, differently from the external humidity uptake, this phenomenon is intrinsic to the cell materials [69]. As a matter of fact, organic polymers, can make better films than small molecules, often pinhole-free and since their high hydrophobic nature they often overcome the small molecules also in term of perovskite protection from both liquid water and humidity. Moreover, the extension of conjugation in the polymers and the probability of contacts among the different polymer chains, often can help to obtain high hole mobility and to improve whole PCE performances. As it will be shown later, the dopant-free behavior was observed first on small molecules, which are essentials in the attempt to derive some structure–property general relationships.

### 3.2. Dopant-Free Organic HTM Polymers—Why?

**Scheme 2:**
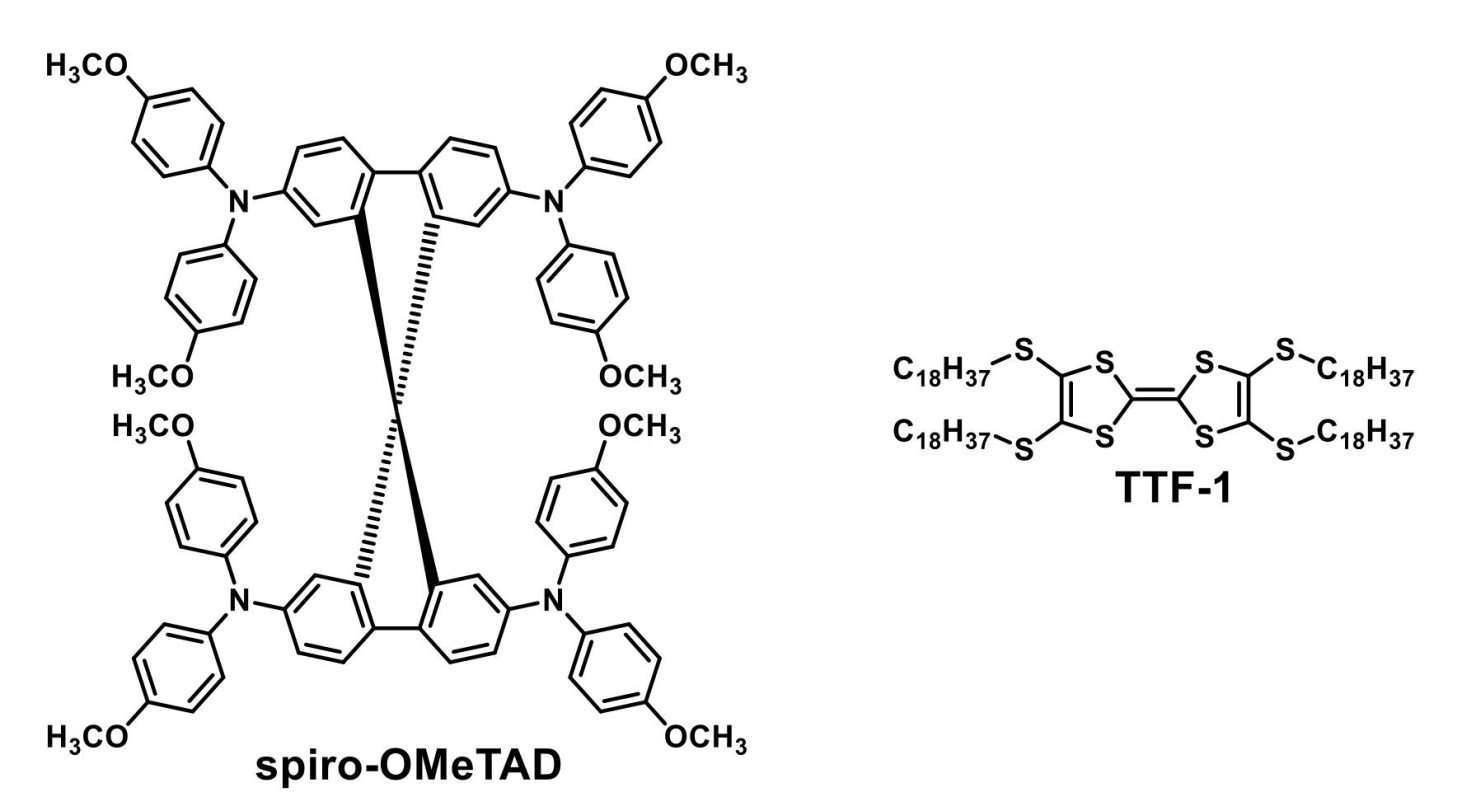
The HTM reference small molecules, **spiro-OMeTAD** and first dopant-free HTM, **TTF-1**.

The above considerations pushed the researchers towards the concept of dopant-free HTMs and forced them to try to prepare and exploit them. The dopant-free organic HTMs are materials showing higher conductivity with respect to most classic HTMs, also in the undoped state. Sometimes, their conductivity is higher in the undoped than in the doped one, and this makes them very attractive to avoid the use of dopants in view of practical applications [12,16]. The main concept to build a dopant-free HTM is to assemble moieties with different (acceptor/donor) characters from each other so that the final structure is highly polarized and can, in principle, receive electrons on one side and release them on the other side. Several organic groups were built to play the role of donors and acceptors and their combinations are almost infinite. Moreover, also the assembly and packing of molecules in the solid-state are important factors to give enough fast hole transfer, since close contacts among the electroactive centers of molecules are crucial to transfer charges efficiently. In this respect, the state-of-the-art small molecules HTM, **spiro-OMeTAD** (*N*^2^,*N*^2^,*N*^2′^,*N*^2′^,*N*^7^,*N*^7^,*N*^7′^,*N*^7′^-octakis(4-methoxyphenyl)-9,9′-spirobi[fluorene]-2,2′,7,7′-tetraamine, see Scheme 2) needs a thick layer (around 140 nm) to work properly and thus the doping is essential to overcome its low inherent conductivity [70]. HTMs showing in their pristine state higher hole mobility and conductivity should afford very thin HTM layers that will properly work without any doping. All compounds whose data are reported in Tables and structures in Schemes, will be reported in bold throughout the review to help the reader.

The first occurrence of an organic dopant-free HTM was found in 2014, with the simple small molecule **TTF-1** (Scheme 2) [71], which gave a respectable efficiency of 11.1%. In 2015 the PCE of dopant-free organic HTMs approached the 15% limit with the DOR3T-TBDT HTM (14.9%) [72] and after that, the efficiency of dopant-free PSCs increased abruptly, up to the actual 21.17% for MPA-BTTI [73], as a result of an enormous and thoughtful research effort. While the small molecules are easier to be prepared and controlled in their structure with respect to polymers, the latter play an essential role for PSCs, since their filming ability can help to achieve better protection of the perovskite layer and their long conjugated main skeleton can give longer charge-diffusion length. The dopant-free polymeric HTMs topic showed an impressive growth in these years since several findings showed that a reasonable control of the properties can be achieved by tuning the structure and polymer characteristics. With this in mind, we approached to review them as peculiar materials for PSCs.

### 3.3. Organic HTM Polymer Classification

Polymers were largely exploited in organic electronics, such as OPV [74], OFET [75], OLED [16,76], etc. Since the appearance of PSCs, they quickly appeared onto the scene [77]. Polymers used as HTMs can be classified as homopolymers, copolymers and D-A (donor–acceptor) copolymers. Necessarily, this classification is a large approximation. The homopolymers were widely studied since their availability and relatively easy synthesis, mainly in the doped state since the polarization, as for small molecules, is important to increase the hole transport ability. Notwithstanding, the two main homopolymers used as HTMs were **P3HT** and **PTAA**, the last one being considered, undoubtedly, the main reference standard (PCE 22.6% in its doped state) [5]. A **PTAA** (doped with tBP and LiTFSI), in fact, was used in normal planar cells, based on FTO/c-TiO_2_/MAPbI_3_/PTAA:tBP + Li–TFSI/Au architecture, showing a behavior with little hysteresis between the forward and back scan [78]. The efficiency was quite good (17.2%) and it showed to be quite stable. The same **PTAA** was used in PSCs where the perovskite was prepared by modulating an excess of iodide ions added to the formulation [5]. This gave very high-quality perovskites, for which the PCE reached outstanding values, such as 22.1% in small cells (22.6% for the best cell) and 19.7% in large cells. Stability tests (ISO 170025) demonstrated that the devices maintained at least 93% of the original PCE after storing at ambient conditions and 40% relative humidity for 13 months. However, no dopant-free applications of **PTAA** were found to approach these excellent PCE results, while, on the other hand, **P3HT** showed great improvements.

In fact, very recently, **P3HT** reached an impressive efficiency (average: 22.7%, best cell: 23%) in its undoped state, thanks to a specifically engineered perovskite, which was passivated by the deposition of a very thin layer (a so-called 2D layer) of a wide bandgap perovskite over the main perovskite layer, separated by a double layer of a surfactant hexyltrimethylammonium bromide [79]. This is the best result ever obtained in the domain of dopant-free HTMs. This particularly engineered system can open a new way to improve the PSC efficiency and could revolutionize the use of polymers as HTMs.

#### 3.3.1. Homopolymers

**Scheme 3:**
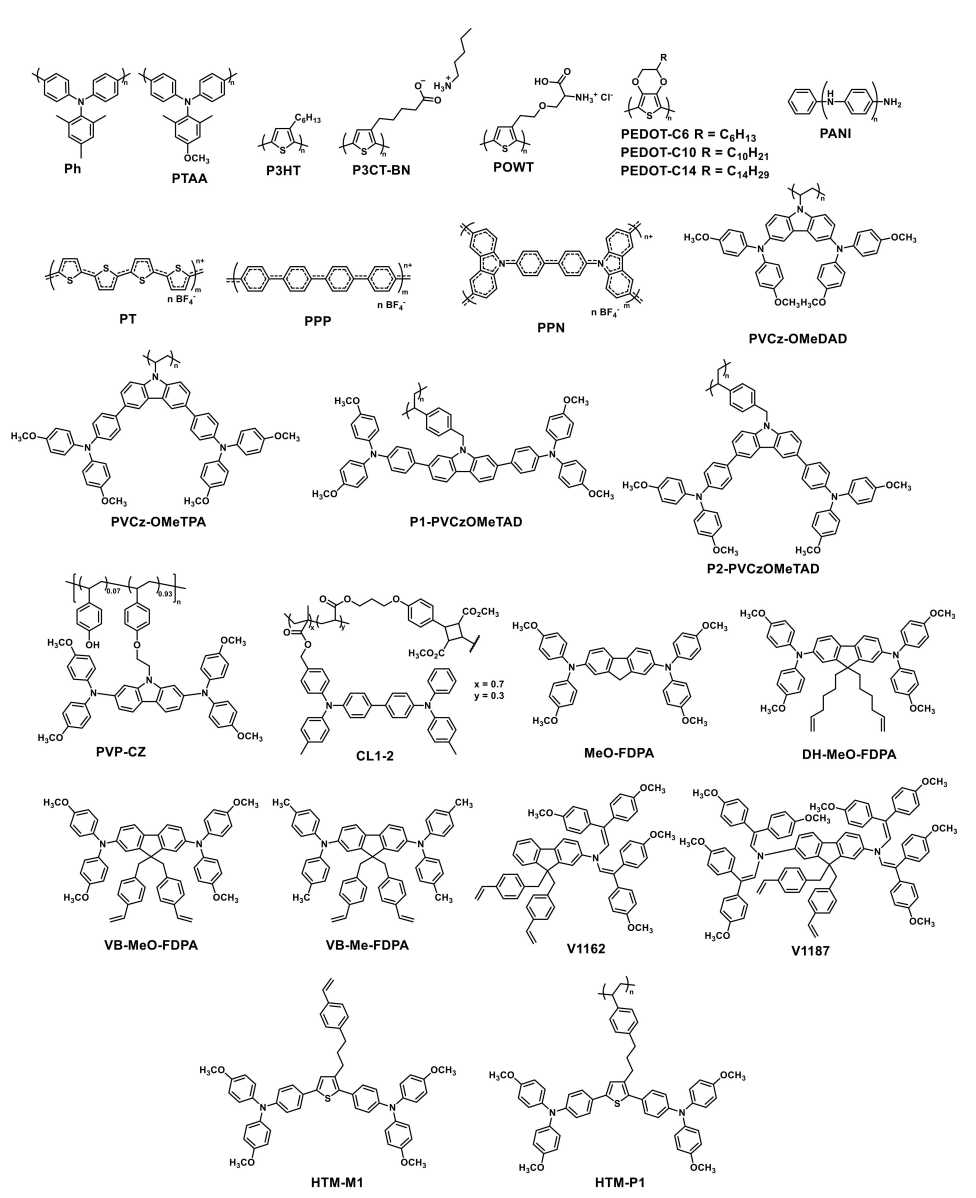
Classical homopolymeric HTMs and some dopant-free polymeric HTMs (or their monomers).

Apart from the already cited **PTAA** [5], and **P3HT** [79], the latter showing an impressive PCE in the undoped state, few homopolymers were produced and tested in PSC (Scheme 3 and Table 1).

The only synthesis, present in literature, of the common **PTAA** was recently published and involves the Suzuki coupling. Two different batches were produced using different catalytic systems, showing a 10.7 kDa and 20.8 kDa molecular weight respectively [80]. It was shown that the highest molecular weights are beneficial for the photovoltaic behavior, greatly enhancing the fill factor. Into a planar PSC, with the MAPbI_3_ perovskite, the best material gave a PCE of 17.7% thus overcoming the commercial reference sample (16.7%).

A modified PTAA polymer was produced, **CH_3_O-PTAA**, and the difference between the presence of a methyl (common **PTAA**) or methoxy group in para position of the pendant phenyl group (**CH_3_O-PTAA**) was explored [81]. In PSC made with the (FAPbI_3_)_0.85_(MAPbBr_3_)_0.15_ perovskite, the **CH_3_O-PTAA** HTM showed an outstanding PCE value of 18.75%, while **PTAA** gave only 14%, essentially due to a higher fill factor. The whole behavior was attributed to the ability of the methoxy groups to stabilize the radical cation form on the HTM during the hole transport.

**P3HT** was also used in an undoped state into a PSC based on a more classical perovskite solar cell, with the FTO/ALD–TiO_2_/CH_3_NH_3_PbI_3-x_Cl_x_/P3HT/Ag architecture. The crucial effect of the deposition method for TiO_2_ was shown: the atomic layer deposition method (ALD) was superior to a commonly spin-coating deposited TiO_2_ layer, giving a higher PCE (13.6% vs. 8.7%) [82].

The dopant-free **P3HT** was also used in all-inorganic CsPbI_2_Br perovskite, in inverted PSCs geometry [83]. The cells were built using a very thin interlayer of poly[(9,9-dioctylfluorenyl-2,7-diyl)-co-(4,4′-(*N*-(4-sec-butylphenyl)diphenylamine)] (TFB) that was deposited between CsPbI_2_Br and **P3HT** since its deeper HOMO helps to regulate the energy level alignment of the different layers. In those conditions, the PCE reached 15.50% while the stability at room temperature and under 25% of relative humidity was very high, by maintaining 98% of the pristine PCE over 1 month.

With **P3HT** properties in mind, poly[3-(4-carboxybutyl)thiophene] (**P3CT**) was designed by Zhang and co-workers, transformed into its butylammonium salt (**P3CT-BN**) to improve its solubility in organic solvents, and finally used in PSC. [84] The presence of the carboxylic acid oxygens helped to passivate the uncoordinated Pb^2+^ surface traps at the perovskite/HTM interface. The **P3CT-BnN** HTM achieved a PCE of 19.05% vs. 18.26% for doped **spiro-OMeTAD**. The **P3CT-BN** cells maintained 80% of PCE after 1800 h at 25 °C, in the dark and at 40% of relative humidity.

The **P3HT** performances were also improved by introducing a polyelectrolyte interlayer based on **P3CT-BN** between the perovskite active layer and the **P3HT** [85]. The thin film of **P3CT-BN** passivated the perovskite surface traps and helped **P3HT** to achieve a better deposition onto perovskite. As a matter of fact, the PCE of undoped **P3HT** grew by 13.13% with no interlayer, up to 19.23% when the interlayer was present. Finally, this treatment also improved the device stability, which retained 80% of the original PCE after 2300 h, at room temperature and 50% relative humidity conditions. The use of polyelectrolytes as HTMs and especially as a thin layer to passivate the perovskite surface Pb traps and to help the perovskite wetting by the HTM increased considerably in the last two years. Several examples will be found during this review.

A modified **P3HT**, coded **POWT,** was built from a monomer containing a lateral chain with an amino acid in the fully protonated state [86]. The synthesis of the polyelectrolyte was performed starting from the 2-(3-thiophene)acetic acid, which was reduced to an alcohol and activated as tosylate, which was finally reacted with *N*-t-Boc-L-serine. The final monomer was simply polymerized by the oxidant FeCl_3_, which at once performed the polymerization and the amino acid deprotection. This polymer showed the HOMO at −5.48 eV and high hole mobility (5.74 × 10^−3^ cm^2^ V^−1^ s^−1^) and could form very good and homogeneous films. In the inverted PSC architecture, **POWT** delivered a good PCE, 17.50%, overcoming the reference **PEDOT:PSS** and showing promising stability, over 54d for non-encapsulated cells under nitrogen at room temperature.

Always structurally related to the **P3HT** polymer, the PEDOT was modified by adding a lateral hydrophobic chain of different lengths, obtaining **PEDOT-C6**, **PEDOT-C10** and **PEDOT-C14** which were characterized and used as HTM for mesoscopic PSCs with (FAPbI_3_)_0.85_(MAPbBr_3_)_0.15_ perovskite [87]. These polymers were prepared from the dibrominated monomer which was reacted with further bromine in CHCl_3_. The HOMO, bandgap, stability (around 250 °C) and hole mobility (around 1 × 10^−4^ cm^2^ V^−1^ s^−1^) were very similar for all those polymers. Most of the photovoltaic behavior was influenced by the morphology of the solid-state film. AFM measurements showed that **PEDOT-C6** was unable to form good compact films, while **PEDOT-C10** and **PEDOT-C14**, being more hydrophobic, showed good homogeneous films. The latter were discriminated by their hole mobility (1.25 × 10^−4^ and 0.92 × 10^−4^ cm^2^ V^−1^ s^−1^). The final PCE was 12.1% for **PEDOT-C6**, 16.2% for **PEDOT-C10** and 14.8% for **PEDOT-C14**, respectively. The stability was measured only for the best performing **PEDOT-C10**, showing the retention of 75% of the pristine PCE after 120 h at rt and 80% of relative humidity).

Xiao and co-workers developed a PSC embodying the homopolymer polyaniline HTM (**PANI**), deposited by electropolymerization with a two-step Cyclic Voltammetry (CV) process [88]. As a particularity of this PSC architecture, the **PANI** was electropolymerized onto m-TiO_2_ obtaining a “brachyplast”, porous, structure in which, finally the perovskite was deposited by infiltration. Due to this specific architecture, this material was shown to behave both as a sensitizer and as an HTM. Different types of devices were prepared. One was doped with a Li^+^ salt, LiTFSI, and tBP to improve the conductivity of the hole transporting layer. This final kind of device showed a PCE of 7.34%. Noteworthy, during a stability test (conditions not specified), those devices maintained more than 90% of their PCE after 1000 h.

Some other electrochemically synthesized polymers, polythiophene (**PT**), polyphenylene (**PPP**) and poly(4,4′-bis(*N*-carbazolyl)-1,10-biphenyl) (**PPN**), were applied to PSCs, with an ITO/HTM/CH_3_NH_3_PbI_3_/C_60_/BCP/Ag architecture (where BCP is bathocuproine, used as a buffer layer) [89]. The polymers were prepared by electrodeposition on the ITO electrode. The HOMO of the polymers are lying at −5.18 eV (**PT**), −5.26 eV (PPN) and −5.31 eV (**PPP**). The last one is better matching the perovskite valence band. The PCE was 14.70, 12.80, 15.80% for **PT**, **PPN** and **PPP**, respectively (**PPP** best cell PCE 16.5%). All these HTMs outperformed the classic PEDOT:PSS (11.0%) initially used as a reference by those authors.

A peculiar case, in dopant-free polymers, is represented by polyvinyl or polystyrene-based polymers, which bear a pendant arm containing an isolated, not conjugated, HTM moiety. Normally, the conductive polymers are always conjugated polymers since they should accommodate charges and transfer them through the conjugated chain by resonance, and the transfer among different chains is obtained by charge hopping or other mechanisms [9,25,27]. In principle, the simple not conjugated polymeric chain with pendant groups is not, per se, a conductive polymer. The crowding of the packed small pendant molecules should maintain them at a very short distance, enhancing π–π stacking interactions and helping the charge transfer. This kind of polymers was also used recently in the field of Organic Photovoltaic Cells (OPV) by crosslinking divinyl triarylamino-based small molecules to give solvent stability to its layer, enabling a layer-by-layer OPV fabrication [65].

The **PVCz-OMeDAD** is a polyvinyl polymer with a pendant “small molecule” HTM residue, as the first example of a series of polyvinyl polymers with a pendant carbazole scaffold that will be reviewed just below [90]. The monomer is a carbazole where the nitrogen is substituted by a vinyl moiety and two bis(4-methoxyphenil)amine are bound at its 3 and 6 positions. The monomer was easily polymerized giving a material showing excellent film-forming ability, proper energy levels and high hole mobility (3.44 × 10^−4^ cm^2^ V^−1^ s^−1^). The PSCs based on **PVCz-OMeDAD** worked with a 30 nm thick HTM thin film, obtaining a PCE of 16.09% [90]. This approach has been taken into account for PSC applications, especially looking towards possible flexible devices.

As a further example of pendant carbazole-based polyvinyl polymers, the **PVCz-OMeTPA** was produced and used along with **PVCz-OMeDAD**, in inverted PSCs with a quasi-2D perovskite [91]. The HOMO levels of **PVCz-OMeDAD** and **PVCz-OMeTPA** were −5.24 and −5.38 eV respectively and the authors mentioned that the last one matching better with the perovskite valence band. Into PSC cells with structure as ITO/HTM/quasi-2D perovskites/PC_61_BM/Cr/gold, the **PVCz-OMeDAD** and **PVCz-OMeTPA** showed efficiencies as high as 14.76% and 17.22%, with the **PVCz-OMeTPA** slightly overcoming the doped **PTAA** (17.10%). These efficiencies complied with the hole mobility values.

In the series of the side-chain polymers, two strictly related analogs of **PVCz-OMeDAD** were prepared by changing the substituents from DPA to TPA. When substituents are in 2.7 position the molecule (**P1-PVCzOMeTAD**) is more conjugated than for 3.6 positions (**P2-PVCzOMeTAD**), as witnessed by the band-gap and l_max_ (B_g_ = 2.95 vs. 3.19 eV; l_max_ = 377 vs. 333 nm) [92].The molecular weight of 9.4 kDa for **P1-PVCzOMeTAD** and 12.8 kDa for **P2-PVCzOMeTAD** were obtained. Excellent thermal properties were obtained and T_g_ was found at 188 °C and 191 °C for **P1-PVCzOMeTAD** and **P2-PVCzOMeTAD**, respectively. The hole mobility was 1.12 × 10^−4^ cm^2^ V^−1^ s^−1^ for **P1-PVCzOMeTAD** and 1.32 × 10^−4^ cm^2^ V^−1^ s^−1^ for **P2-PVCzOMeTAD**. When those HTMs were applied by spin coating with chlorobenzene onto the Cs-FAMA perovskite, the best PCE obtained was 16.78% for **P1-PVCzOMeTAD** and 18.45% for **P2-PVCzOMeTAD**. For **P2-PVCzOMeTAD**. Very interestingly, the authors exploited the substitution of chlorobenzene with non-chlorinated solvent (i.e., 2-methylanisole) aiming at an increase in the greenness of the deposition process. In this case, the best PCE was just a bit lower, 16.42%. The usual doping increased the performances to 18.77% for **P1-PVCzOMeTAD** and 19.67% for **P2-PVCzOMeTAD**. The non-encapsulated devices, maintained over 80% of their pristine PCE for both HTMs kept for 30 days at ambient temperature and 30% of relative humidity.

Another polymer, **PVP-CZ** was prepared as a not conjugated polymer with active pendant groups [93]. The key point was that the molecular HTM was prepared apart and linked to a preformed polymer, poly(4-vinylphenol) by the Mitsunobu reaction. This material was transparent, showed relatively low hole mobility (3.1 × 10^−5^) but attained a promising PCE of 17.75% a bit lower than undoped **PTAA** (18.85%) with an excellent fill factor (81.07). This interesting preparation method showed that it can be possible to rely on preformed polymer and make attracting to access to the enormous number of small molecule HTMs and to link them to a good quality polymer. All these characteristics make possible a fine-tuning of both polymeric and HTM properties.

The cross-linkable polymer P1-2 was prepared by polymerizing a TPD methacrylate monomer with a cinnamate-acrylate monomer, which was crosslinked onto the ITO electrode in the PSC device at 130°C after the deposition, giving **CL1-2** [94]. The HTM was transparent and was ideally exploitable for inverted PSCs. The HTM film on ITO was smooth and homogeneous and promoted the growth of larger grains of perovskite with respect to the standard polyTPD. When used in based PSCs, the PCE of **CL1-2** was 18.7% substantially better than the 16.0% shown by polyTPD.

Starting from the **MeO-FDPA**, a small molecule that powered a PCE close to 14%, Zhang et al. prepared similar molecules by appending different cross-linkable residues [95]. A thermal polymerization method without the requirement of additives or initiators ensured the absence of further not predictable impurities and promoted the formation of solid 3D polymeric networks. The **DH-MeO-FDPA** contained a chain with a terminal alkene, while the other two molecules **VB-MeO-FDPA** and **VB-Me-FDPA** had styryl derivatives. It was shown that the alkyl chain gave more conformational freedom with respect to the styryl derivatives, but the latter led to a lowering of the HOMO. The styryl molecules showed a hole mobility one order of magnitude higher than **VB-MeO-FDPA** and **DH-MeO-FDPA**, similar to **spiroOMeTAD**. Finally, in the preparation of planar cells, **VB-MeO-FDPA** helped to obtain larger perovskite grain thus reducing the charge recombination. As a result, the PCE was 15.9% for **DH-MeO-FDPA**, 18.7% for **VB-MeO-FDPA** and 17.9% for **VB-Me-FDPA**. This remarked the importance of the alkoxy oxygen as a donor group on the DPA structure. These molecules are relatively easy to prepare and can be quickly used for the preparation of HTM films.

Other crosslinkable polymers were prepared on the fluorene scaffold, to which two vinyl groups were attached, obtaining **V1162** and **V1887** [96]. The **V1162** and **V1887** HTMs were synthesized by coupling a fluorene diamine with the 2,2-bis(4-methoxyphenyl)acetaldehyde, forming an enamine and the final compounds were prepared by reacting the 4-vinylbenzyl chloride with the fluorene intermediates, benzyltriethylammonium chloride and sodium hydroxide. This way of synthesis was attractive since the materials are considerably cheap (price under 17 euro per gram). The DSC measurements showed that just above the fusion temperature (228 °C for **V1162**) an exothermic process takes place, which was identified as polymerization. The **V1187** material was amorphous (T_g_ at 139 °C) and its polymerization started at 190°, with a peak at 239 °C. The full crosslinking was achieved in about 15 °C of heating. In the PSC based on the Cs_0.05_(FA_0.83_MA_0.17_)_0.95_Pb(I_0.83_Br_0.17_)_3_ perovskite, **V1162** and **V1887** achieved PCEs of 14.49% and 15.51% as small molecules and of 14.71% and 16.77% as crosslinked polymers, respectively. An optimization study of **V1187**, trying to choose the best concentration for the deposition showed that at 1.5 mg/mL, a PCE of 18.14% was obtained after crosslinking.

Polystyrene containing a pendant triarylamine small molecule was prepared by Wu and coworkers [97]. The monomer was obtained by coupling two *N*,*N*’-bis(4-methoxyphenyl)-*N*’’-phenylamine moieties with thiophene, to which styrene was connected in a not conjugated way. This monomer was polymerized, giving the polymer **HTM-P1**. It showed the HOMO at −5.18 eV and LUMO at −2.35 eV, both slightly lower than that of the monomer. The thermal stability was good (317 °C) and the T_g_ was relatively high (132 °C), avoiding any HTM modification induced by the heating of the device in operating conditions. The hole mobility was remarkably high, 1.6 × 10^−4^ cm^2^ V^−1^ s^−1^, higher than its monomer (6.1 × 10^−5^ cm^2^ V^−1^ s^−1^): in cells based on triple cation perovskite (Cs_0.05_FA_0.81_MA_0.14_PbI_2.55_Br_0.45_) the **HTM-P1** gave a 16.8% PCE (best cell 17.2%) while its monomer showed only 9.1%. The stability of **HTM-P1** based cells was evaluated on not encapsulated devices, showing a PCE retention >80% after 20 days [97].

These two last examples showed the importance of this approach. The not-conductive polymers can keep a lot of pendant “small molecule” HTMs close to each other so that the contact for ensuring hole transport is assured. The efficiencies obtained with **PVCz-OMeDAD** and **HTM-P1** account for a possible evolution towards better efficiencies, by modifying both the kind of polymeric skeleton and the small molecule HTM pendant arm.

#### 3.3.2. D-A Copolymers

Throughout this section, we will discuss the D-A polymeric HTMs used in PSC, relating the polymer performances to its structural building blocks. The main way to approach this discussion is to evidence a building block and to collect all the polymers containing it, to discuss the differences in structure, properties, morphological behavior, and photovoltaic performance.

We can remark that starting in 2014, just after the beginning of the PSC “story” (in 2012), also the dopant-free HTMs are emerging as an important and necessary component of the solar cell, even because of the strict requirements for HTM and PSC stability [71].

##### 3.3.2.1. DPP (2,5-Dihydropyrrolo[3,4]Pyrrole-1,4-Dione)

**Scheme 4:**
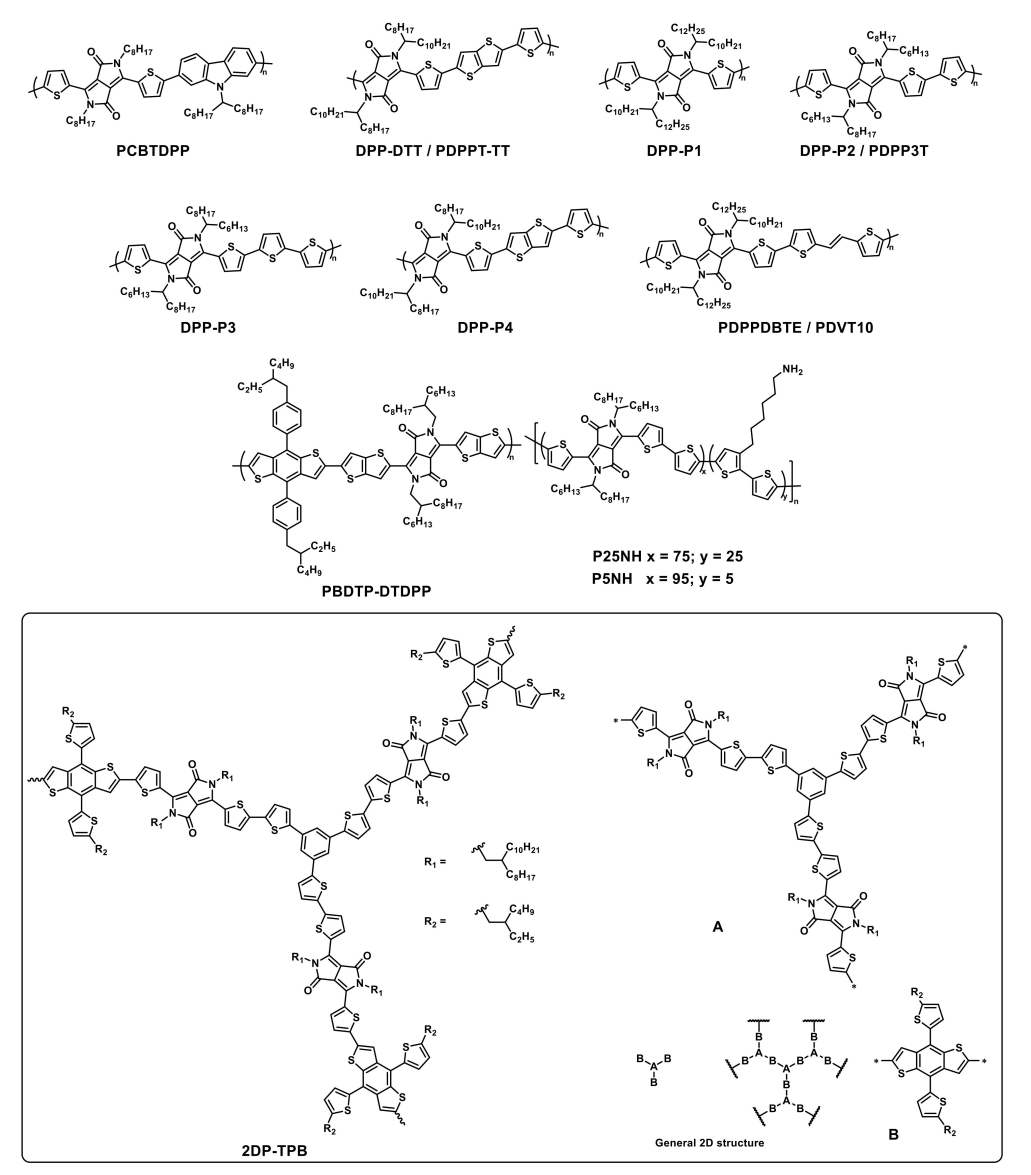
Polymers containing the 2,5-Dihydropyrrolo[3,4]Pyrrole-1,4-Dione (DDP) group.

In 2013, the first report of dopant-free polymeric HTM used in a perovskite solar cell was a copolymer, **PCBTDPP**, made by carbazole, thiophene and 2,5-dihydropyrrolo[3,4]pyrrole-1,4-dione units (DPP) bearing alkyl chains to increase its synthetic processability (for all polymer of the DPP serie please see Table 2 and Scheme 4) [77]. This material showed a correctly aligned HOMO level (−5.34 eV), and very high hole mobility (0.02 cm^2^ V^−1^ s^−1^). The application in both MAPBr_3_ and MAPbI_3_ gave 3.3% and 5.5% PCE, respectively. While being far from the best results, the ability of polymeric HTMs to work in PSC cells was demonstrated and was greatly improved during the years. The polymer **PCDTBT** (see below the Section 3.3.2., related to benzothiadiazole) is an analog of **PCBTDPP** in which the acceptor DPP was substituted by a benzothiadiazole (BTZ) [98]. The substitution of DPP with BTZ did not improve the efficiency of MAPbI_3_ perovskite solar cells, which remained low (2.4%).

Conversely, when **PCDTBT** and **DPP-DTT** were used to modify the surface of an ITO/PEDOT:PSS interface, they helped to prepare very good crystalline perovskite by vacuum deposition. In these inverted PSC, the **PCDTBT** polymer (PCE: 16.5%) outperformed **DPP-DTT** (8%, not optimized), and the other polymers were taken into consideration, namely PCPDTBT and **P3HT** [99]. The higher performances were ascribed to the better alignment of energy levels of **PCDTBT** with the perovskite, the higher crystallinity of perovskite and its optimized contact with the polymeric substrate. In this case, the polymer layer was thinner enough (less than the tunneling distance, about 10 nm) so that the polymer could not limit the charge transport. This was a different condition with respect to most PSCs studied until now, where the HTM are consistently thicker. This was the first excellent result achieved by polymers in PSCs.

Several polymers, in which the DPP was alternated to a few thiophenes, linked in different ways, were prepared.

As an example, the already cited **DPP-DTT** was made by connecting a DDP unit with the 2,5-di(thiophen-2-yl)thieno[3,2-b]thiophene. The **PDPPT-TT** polymer, which has the same structure as **DPP-DTT** was used on normal PSC, in which PCBM was interposed between TiO_2_ and the perovskite (MA_0.85_FA_0.15_PbI_0.85_Cl_0.15_) layer. After having created an HTM layer onto the perovskite, the film was exposed to solvent vapor, i.e., the solvent vapor annealing process. This process is an alternative to thermal annealing and can be applied at room temperature and, can save energy and make the production processes easier. The pristine polymer showed an average PCE of 14.3% and the solvent annealing allowed to increase it up to 16.2%, overcoming the doped **spiro-OMeTAD** which reached 15.8%. The best device obtained after solvent vapor annealing reached a PCE of 17.8% [100]. This was an outstanding result that showed that each polymer can behave very differently, depending on several conditions: (i) the kind of cell in which it is applied, (ii) the morphology it adopts at the surface, (iii) post-treatment or post-processing of the deposited film, etc. It is worth mentioning that in several cases it is difficult to relate the photovoltaic properties to some molecular properties since often not all the characteristics of the polymers are disclosed, such as molecular weight, regioregularity, if any, sometimes simply because they are commercial materials. Indeed, it is well known to polymer chemists that it is difficult to maintain exactly the same characteristic on different batches, so the disclosing of the exact molecular properties has great importance to establish structure–performance relationships.

To build D-A alternated polymers, the DPP was coupled with bisthiophene (**DPP-P1**), terthiophene (**DPP-P2**, also reported as **PDPP3T**), tetrathiophene (**DPP-P3**) and the 2,5-di(2-thienyl)thieno[3,2-b]thiophene (**DPP-P4**, analog of **PDPPT-TT**, with different chain length) [26]. These materials showed HOMO energy level lying at −5.35 eV (**DPP-P1**)), −5.20 eV (**DPP-P2**), −5.25 eV, (**DPP-P3**), −5.19 eV (**DPP-P4**) high thermal stability, a lamellar packing obtained by π-stacking with π–π contacts as short as 3.6 Å (**DPP-P4**). In the conditions used in this study, the **DPP-P2** and **DPP-P4** showed very high hole mobility (4.4 and 3.7 × 10^−2^ cm^2^ V^−1^ s^−1^). The **DPP-P2** and **DPP-P4** showed a reasonable PCE (7,1% and 9.3% respectively) on MAPbI_3_ perovskite which only for **DPP-P4** (**PDPPT-TT** with different chain length) became very good on Cs (14.2%) and Rb (16.3%) based perovskite, respectively [26]. It is worth mentioning that the **DPP-P3**, containing three thiophenes, showed a lower PCE, probably due to a shorter conjugation length, ascribable to the repetition of small aromatic units. The **DPP-P4** which contains one thienothiophene can probably extend better the conjugation and promote p–p stacking.

In another paper, the polymer **PDPP3T**, reported above as **DPP-P2** and containing three thiophene rings, was prepared and used within mesoscopic PSCs. Its HOMO was lying at −5.3 eV, properly aligned with the perovskite MAPbI_3_ [101]. Very interestingly, in its pristine state, it showed the same PCE as doped **spiro-OMeTAD** (12.3%). The **PDPP3T** can transport charges faster than **spiro-OMeTAD**, and since the charge transport is faster than the charge lifetime, both HTMs are working proficiently. Stability tests on non-encapsulated devices at room temperature and 20% of relative humidity showed a better endurance of the **PDPP3T** over **spiro-OMeTAD**, retaining 40% of the starting efficiency after 172 h, vs. the 17% of the **spiro-OMeTAD**.

It is clear that the use of a different number of thiophenes as the spacer between DDP units can tune the properties of the polymer, but it is also evident that when too much conformational ability is given to the different rings, there is a high proneness to tilt them and to reduce the p–p stacking ability of the polymer in the solid-state. This negatively influences photovoltaic performances. The general outcome is that by assembling large fused ring systems and limiting the number of single bonds in the “monomeric unit”, one should achieve better morphological characteristics of the film and thus higher performances.

A polymer based on a diketopyrrolopyrrole and thiophene, **PDPPDBTE**, was prepared by surrounding DPP on each side with two thiophenes and the polymeric connections were performed with an ethylene group [102,103]. The material was originally implemented in OFET, showing very high hole mobility (0.32 cm^2^ V^−1^ s^−1^) in undoped state and conductivity (10^−2^ S cm^−1^), substantially better than **spiro-OMeTAD** and **P3HT**, thus paving the way to a **PDPPDBTE** application in PSCs. The use in MAPbI_3_ based mesoscopic PSCs gave a good PCE (9.2%), exceeding the values obtained by both **spiro-OMeTAD** (7.6%) and **P3HT** (6.3%) in the same configuration. Excellent stability was shown by the **PDPPDBTE** based PSCs, which maintained >80% of the pristine PCE over 1000 h, while **spiro-OMeTAD** maintained >70%, for non-encapsulated devices, at rt and 20% of relative humidity.

The **PDVT-10** polymer is the same material as **PDPPDBTE**. It was used by Liu and co-workers in mesoscopic MAPbI_3_ PSCs, and showed a great film-forming ability after annealing 5 min at 150 °C. This characteristic could give good protection of perovskite and increase stability [104]. The material showed suitable HOMO, located at −5.28 eV. The hole mobility of **PDVT-10** (doped) was exceptionally high (8.2 cm^2^ V^−1^ s^−1^). The PCE (12.97%, best cell: 13.4%) was close to that of a commercial **PTAA** (11.28%, best cell: 14.60%), but with improved reproducibility. The authors attributed the good results to the high coplanarity of the DPP with its two thiophenes on both sides, due to hydrogen bonding of the DPP carbonyl groups with the b-hydrogens of the thiophenes. No other information could be found in the related papers [102,103,104], to establish the different performances of **PDPPDBTE** and **PDVT-10**. The stability test evidenced the better performance of **PDVT-10**, which survived to 20% humidity for 20 days, at room temperature and under the light, without appreciable degradation. On the contrary, **PTAA** was heavily degraded after 5 days and fully degraded after 20 days.

The polymer **PBDTP-DTDPP** contains both DPP and benzodithiophene (BDT). For more information about BDT-based HTMs please see paragraph 3.4.2.2 on (BDT, Benzo[1,2-b:4,5-b’]dithiophene) cores spaced by the bithiophene was prepared and applied on planar PSCs [105]. The presence of two bithiophenes around the DPP core was enhancing the proneness to obtain highly ordered solid-state films, by p–p stacking. Photophysical measurements showed an extremely fast separation charge process, in the range of picoseconds. Solar cells based on the triple cation perovskite Cs_0.05_(MA_0.17_FA_0.83_)_0.95_Pb(I_0.83_Br_0.17_)_3_ showing a PCE 14.73% slightly lower than **spiro-OMeTAD**. Notably, this material did not show any positive or negative effect ascribable to doping (PCE 14.00%).

The DPP based polymer **P25NH** was produced by reacting the 3,6-bis(5-bromothiophen-2-yl)-2,5-bis(2-hexyldecyl)pyrrolo[3,4-c]pyrrole-1,4(2H,5H)-dione monomer with a mixture 4: 1 of 2,5-bis(trimethylstannyl)thiophene and 2-(6-(2,5-Dibromothiophen-3-yl)hexyl)isoindoline-1,3-dione, to obtain a polymer in which the amino hexyl pendant chain was present in a average ratio of 3:1 [106]. The skeleton of the **P25NH** is very similar to the **DPP-P3** and the only difference is the presence of further thiophene in **P25NH** than in **DPP-P3**; the presence of the aminohexyl chain which is used as a passivating agent for perovskite surface traps is the only other difference. This material gave lamellar films which enabled excellent hole mobility (0.021 cm^2^ V^−1^ s^−1^). The amino pendant group was able to passivate surface perovskite hole traps thus increasing the PCE of **P25NH**, which was 18.1%, higher than the 15.5% obtained for the standard **P3HT**.

As an evolution of this work, the authors tried to further improve the hole mobility of this kind of compound, by modulating the ratio between the two monomers, obtaining excellent results with the 95:5 ratio [107]. The increase in hole mobility of **P5NH** with respect to **P25NH** can afford thicker films to better protecting the perovskite without sacrificing the performances. The XRD analysis showed weaker reflections for the perovskite crystals, due to the passivating effect of the amino group at the surface Pb atoms, but no reflections were found for the HTM, showing that it is amorphous in the solid-state. The polymer **P5NH** which contains only 5% of the 3-aminohexylthiophene monomer showed a hole mobility increase of about 150% with respect to the polymer **P25NH**. The final PCE was 18.1%, with a remarkably high fill factor (83.1%).

A tripodal 2D polymeric HTM, **2DP-TDB**, was produced by Fu et al. on the 1,3,5-tri(thiophen-2-yl)benzene(TB) scaffold [108]. On this scaffold, the arms were constituted by a DPP monomer with one thiophene at each side. The resulting small molecule (**TB-DPP**) was thus brominated on each terminal thiophene unit and polymerized by the Stille protocol with a BDT monomer. This material showed excellent properties, being prone to p–p stacking and organizing as a face-on layer on the perovskite (from GIWAXS measurements and temperature-dependent aggregation from UV spectra) and showing reasonable hole mobility. The 2D arrangement of this polymer is derived from a concept now largely used for 2D star-shaped small molecules, such a those based on truxenes [23]. The 2D star-shaped polymers can enforce the molecular interactions making the hole transport easier. As evidence, the monomer **TB-DPP** was substantially less active in PSCs (PCE: 11.61%) than **2DP-TDB** (PCE: 21.53%), also due to the better passivation ability of the polymer over the related small molecule. When the perovskite was improved by adding the 4-fluorobenzamide in small quantities to the precursor’s mixture, the resulting material was able to push the final PCE of **2DP-TDB** up to the record value (22.17%) obtained up to now for planar PSCs.

##### 3.3.2.2. BDT (Benzo[1,2-b:4,5-b’]Dithiophene)

The benzodithiophene core (BDT) is an inherent electron donor group. It was inserted into molecules and polymer with simple synthetic approaches. The starting material is normally the benzo[1,2-b:4,5-b’]dithiophene-4,8-dione, which can be modified to insert alkyl [109,110] or alkoxy chains [109,111,112] or further thiophenes bearing alkyl chains [113,114]. We will analyze several HTMs structures bearing this group since it was one of the most promising donors used in dopant-free polymers [115,116].

**Scheme 5:**
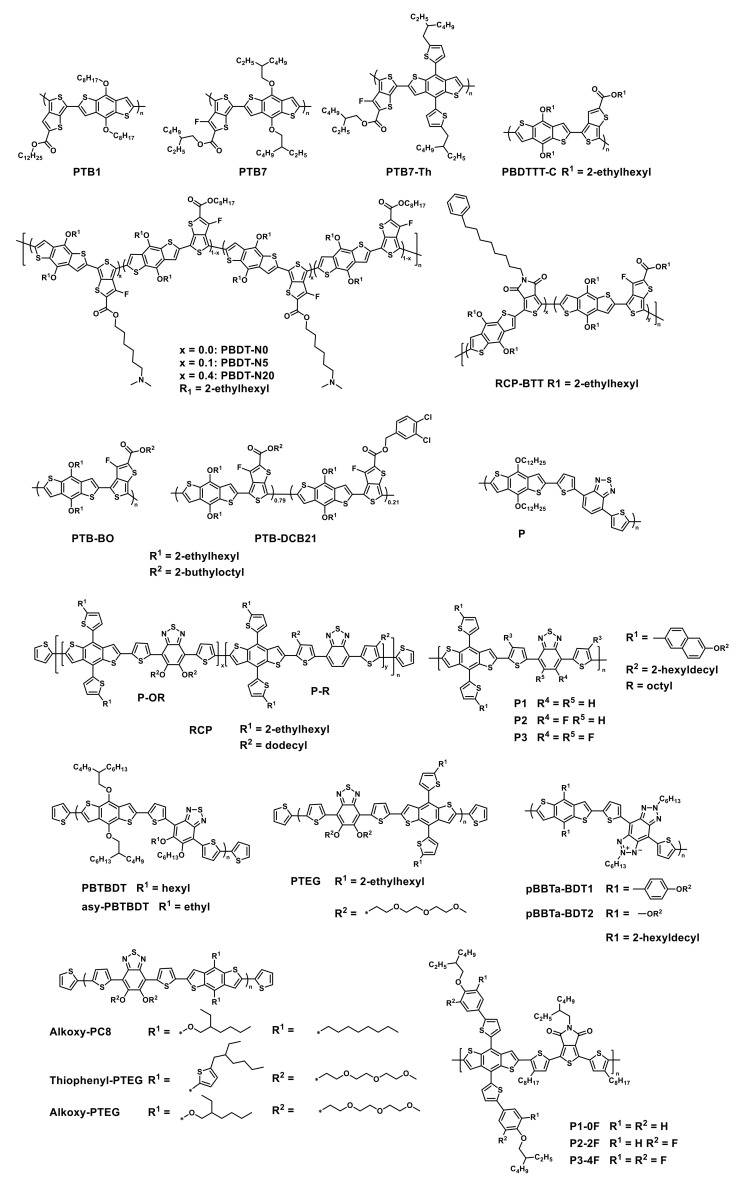

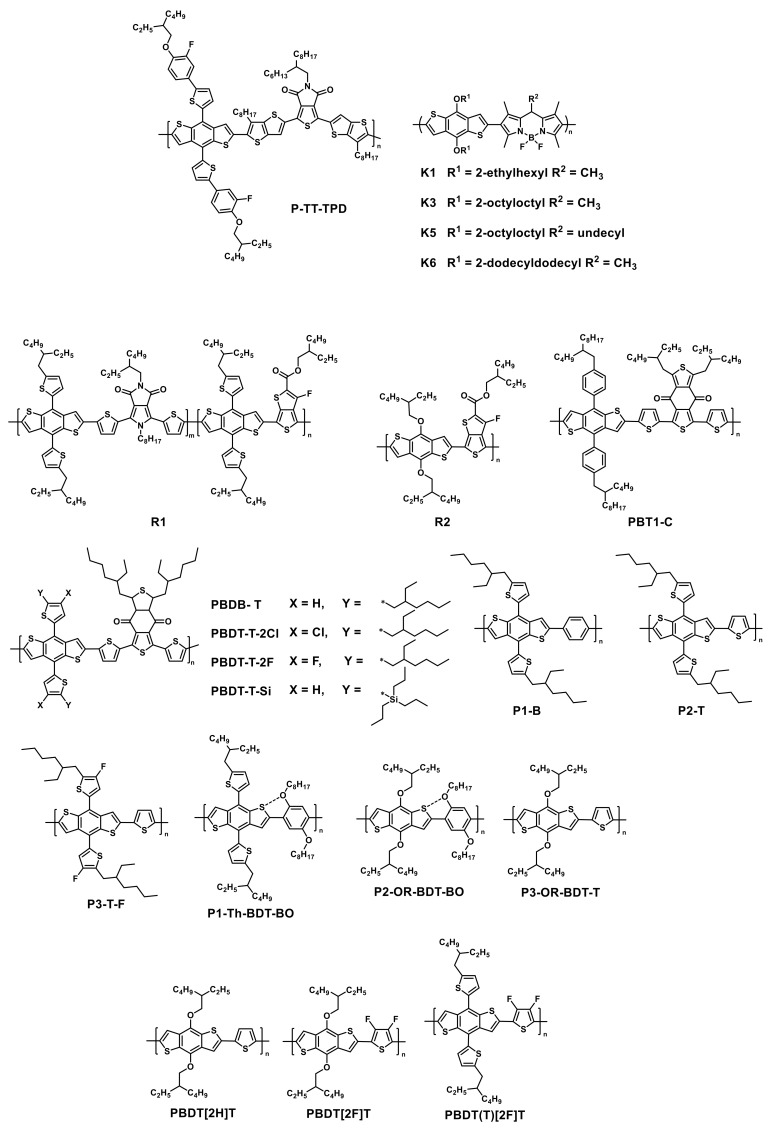
HTMs containing the BDT group.

The first BDT containing HTM in the PSC field appeared in 2014 (for all BDT-based polymers please see Table 3 and Scheme 5). The general characteristic of the **PTB7** and related polymers is to have a BDT nucleus, bearing two alkoxy chains, coupled with a thieno[3,4-b]thiophene, the 3-fluorothieno[3,4-b]thiophene-2-carboxylic acid, where the carboxylic acid is normally transformed into an ester group.

The **PTB1** and **PTB7** polymers were based on BDT and on the fluorinated thieno[3,4]thiophene (TT) [117]. The **PTB1** bears octyloxy chains on BDT and a dodecyl ester onto the thieno[3,4]thiophene group, while **PTB7** bears 2-ehtylhexyloxy chain and 2-thienylhexyl ester groups. They were used on mesoscopic PSCs based on MAPI_3-x_Cl_x_, and compared with **PCDTBT**, **PCPDTBT** (PCE for **PCDTBT** was up to 16.5%, for other data on them, see Section 3.3.2.3 on BTZ (Benzothiadiazole) and Cbz (carbazole), and references cited therein). The undoped **spiro-OMeTAD**, taken as a standard, gave a PCE of 10.9%, as a result of a lower back electron transfer with respect to the polymers, since the HOMO of **spiro-OMeTAD** is higher than that of low band-gap polymers. The **PTB7** was, however, performing well, with a PCE of 7.5% higher than the **PTB1**-based counterpart (6.0%). The **PTB7-Th** polymer was used to study the effect of the HTM-perovskite interfacial layer [118]. This HTM belongs to the family of **PTB7** and related polymers, and it is an analog of **PTB7**, where the oxygen of the alkoxy chain moieties on the BDT nucleus is replaced by thiophene. This polymer showed very good hole mobility, and a HOMO with the correct energy to align with the perovskite valence band. The interface between a sol–gel prepared ZnO (as an electron transport layer, ETL) and the MAPbI_3_ perovskite was treated with a PCBM layer. The PCBM helped to modify the interface with ZnO, helping to improve the V_oc_ of the device. As a consequence, the best PCE obtained was 11.04% (average PCE: 10.83%), higher than the value obtained without the PCBM layer, 8.37% (average PCE: 7.65%). This demonstrated the better behavior of **PTB7-Th** when the ETL material was different from the TiO_2_.

The polymers **PTB7** and **PTB7-Th** were tested recently (along with **P3HT** and PCDTBT) in MAPbI_3_ solar cells showing the best PCE for **PTB7** (6.30%) and **PTB7-Th** (3.48%) [98]. During a stability test (dark, room temperature, 55–70% humidity, 66 h) **PTB7-Th** showed a 60% increase of its J_sc_ and of 40% of its PCE performance (final PCE around 4.87% after 66 h.), which was not clarified by the authors, while for **PTB7** the PCE decreased by 40% (final PCE around 3.78% after 66 h.). While not being as active as the previously cited works, it seems that **PTB7-Th** can be more interesting from a stability point of view. Further studies in strictly controlled conditions would be needed to establish what material is the best one.

Three BDT-based polymers, **PBDT-N0**, **PBDT-N5** and **PDBT-N20**, with pendant amino-terminated alkyl chains [119,120]. The main goal was to exploit the amino pendant groups to passivate the perovskite and to link the polymers to the perovskite. Those polymers are based on the skeleton of **PTB7** and were obtained by reaction of the BDT monomer with a mixture of octyl 3-fluorothieno[3,4-b]thiophene-2-carboxylate and 8-(dimethylamino)octyl 3-fluorothieno[3,4-b]thiophene-2-carboxylate in different proportions [120]. Their energy levels were very similar since the percentage content of amino groups did not influence the HOMO, while the hole mobility increased. These polymers were used in mesoporous PSCs with the (FAPbI_3_)_0.85_(MAPbBr_3_)_0.15_ perovskite. The PCE increased by following the content of amino groups, being 12.4%, 15.5% and 18.9% for **PBDT-N0**, **PBDT-N5** and **PDBT-N20**, respectively. This kind of example explains well that the perovskite needs to be passivated in order to perform better and that an HTM should be built carefully, not only taking into consideration its main intrinsic HTM properties.

A random copolymer based on BDT, **RCP-BTT**, was synthesized by the Stille protocol by coupling the stannyl derivative of the BDT partner with a mixture of the 1,3-dibromo-5-(8-phenyloctyl)-4H-thieno[3,4-c]pyrrole-4,6(5H)-dione and the 1-(4,6-dibromo-3-fluorothieno[3,4-b]thiophen-2-yl)-3-ethylheptan-1-one in a ratio 5:95. [121] The HOMO was found at -5.28 eV and the hole mobility was high (0.0023 cm^2^ V^−1^ s^−1^). In a mesoporous PSC based on the Cs_0.05_(FA_0.85_MA_0.15_)_0.95_Pb(I_0.85_Br_0.15_)_3_ perovskite, **RCP-BTT** gave a PCE of 8.45%, higher than the reference **PTB7** (6.91%). Under doped conditions, the **RCP-BTT** obtained a PCE of 14.91%, mainly due to a huge increase of the J_sc_ and fill factor (from 53% to 69%) as supported by AFM analysis which showed a better and homogeneous film formation.

Very recently a fully inorganic quantum dot αCsPbI_3_ perovskite was used with the **PTB7** and **PTB7-Th** polymers, obtaining a minimal V_oc_ energy loss [122]. The record PCE 12.55% was obtained by **PTB7** with this quantum dot (QD) perovskite. In the same conditions, the **P3HT** reached 9.82% and **PTB7-Th** gave 10.60%. The **PTB7** aligns nearly perfectly with the valence band of the QD-perovskite [122]. Strictly related to the **PTB7** polymer family, **PTB-BO** and **PTB-DCB21** were synthetized by Lee and co-workers. Three monomers, a BDT and two different fluorinated thieno[3,4]thiophenes (TT), were used in different combinations, to prepare two similar polymers, to be implemented as dopant-free HTMs, namely **PTB-BO** and **PTB-DCB21** [123]. The BDT nucleus is the same as **PTB7.** The two fluorinated thieno[3,4]thiophene moieties are functionalized with either an alkyl chain (2-butyloctyl) or a dichlorobenzene. The **PTB-DCB21** obtained a PCE of 8.7% while the **PTB-BO** PCE was a little lower, 7.4%, because of general good hole mobilities. It seems that the dichlorobenzene group gives a faster electron transport and retarded charge recombination, due to possible p interaction with the component of the perovskite. It is worth noting that the PCE increased upon aging of the devices probably due to better intermolecular interactions between the HTM and the perovskite.

The **PBDTTT-C** polymer, based on BDT and thieno[3,4]thiophene (TT) moieties, was used to prepare planar PSCs without an ETL material (namely TiO_2_) [124]. This HTM is different from **PTB7** only due to the substituent on the moiety. Instead of a 2-ethyhexyl ester, the 2-ethylhexanoyl group was present. The new material showed higher hole mobility (2.4 × 10^−4^ cm^2^ V^−1^ s^−1^) than **P3HT** (5.1 × 10^−5^ cm^2^ V^−1^ s^−1^), used as a reference, and better alignment of its HOMO with the perovskite valence band level. In the planar PSC configuration, a good PCE (9.95% best cell PCE, 9.32% average PCE) was obtained.

After having thoroughly explored this main backbone for the **PTB7**/**PTB7-Th** family, further combinations of BDT were approached. A noticeably series of results came from the substitution of the thieno[3,4]thiophene unit with benzothiadiazole, which was always bearing two thiophenes at both sides.

A polymer named **P** was obtained as a D-π-A polymer, by coupling a dodecyloxy-substituted BDT monomer and a bis(thienyl)benzothiadiazole. The polymer was applied in cells with FTO/bl-TiO_2_/TiO_2_/CH_3_NH_3_PbI_3_/HTM/Au architecture. It showed a deeper HOMO, as a cause of the strong acceptor character of BTZ. The general parameters, such as increased hole mobility, V_oc_, J_sc_, Fill Factor (FF) and PCE, were better than **P3HT** ones, but far from those needed to obtain a high PCE [125].

A huge breakthrough was obtained by introducing substituents on the BTZ unit and/or on the thiophenes at its sides. An impressive PCE (17.3%) was obtained by Kim et al. who produced a random copolymer (**RCP**) by assembling a donor BDT monomer with two acceptor BTZ-based monomers (that here, for homology, will define with the acronyms: M-R and M-OR) [126].

Small differences are present on the monomers. The BDT monomer contains two thiophenes with a 2-ethylhexyl chain. One BTZ monomer possesses two 2-ethylhexyl chains on the two thiophenes present at both sides of BTZ (M-R) and the other BTZ monomer has the dodecyloxy chains positioned on the BTZ, (M-OR). The random copolymerization of the two monomers by Stille coupling yielded the RCP polymer. Both M-R and M-OR were polymerized with the BDT monomer, giving their respective “homopolymers” **P-R** and **P-OR**. These two homopolymers were reasonably similar to the molecular weight of the **RCP**, since the synthetic methods requested for every polymer a few microwave heating steps (120 °C, 5 min, 140 °C 5 min, 170 °C, 1 h) and two end-capping steps with 2-tributylstannylthiophene at 140 °C 20 min and with 2-bromothiophene for 140°, 20 min). Microwave heating applied to organic reactions normally improves the repeatability and homogeneity of products obtained in different batches and often reduces byproducts [140]. It is however a problem to use this method for industrial production since, for technical reasons, one cannot scale-up easily the system [141]. **P-OR** has the HOMO (−5.51 eV) lying below the MAPbI_3_ perovskite valence band (−5.43 eV), while the **P-R** HOMO (−5.40 eV) is better aligned with the perovskite. As a result, the **RCP** HTM has the HOMO at −5.41 eV, still correctly aligned with the perovskite VB. The hole mobility of **RCP** was higher than the **P-R** and **P-OR** homopolymers. It is worth and essential mentioning that while the **P-OR** homopolymer shows a face-on organization of the polymer onto the perovskite, **P-R** does not show this ability. The **RCP** copolymer improved this property and the ability to order itself at the surface in a face-on way, which is well known to promote an easy hole transfer in the vertical direction, from the perovskite to the metal electrode. All these facts evidence that the **RCP** polymer is beneficial for the PSC application and, consequently, that a possible random copolymerization strategy can be used fruitfully to improve polymer properties. While the PCE of the single homopolymers was low (1.9% for **P-R** and 2.6% for **P-OR**), the combination of the two monomers was strongly synergistic (PCE 17.3%), also overcoming the doped **spiro-OMeTAD** (15.3%). The doped **RCP**-based PSCs showed a PCE of 16.5%, denoting the better performance for **RCP** in the undoped state. Stability tests on not encapsulated devices (over 1400 h at room temperature and 75% relative humidity) demonstrated the absolute stability of the cells containing only **RCP** while the doped **RCP** decreased their performances (maintaining around 40% of the pristine PCE) still being however higher than **spiro-OMeTAD**, which fully decomposed and deactivated after just less than 900 h. A stable PCE of 17.3% maintained for longtime exposure to high humidity showed that PSC can be improved to be amenable to the market, in the next future. Starting from these excellent results, the research further explored the application of polymers based on BDT and BTZ.

Three polymers, named **P1**, **P2** and **P3**, were based on a BDT nucleus monomer, substituted with a 2-(6-((2-hexyldecyl)oxy)naphthalen-2-yl)thienyl group and on a benzothiadiazole (BTZ) surrounded by two 3-octylthiophenes [127]. The only difference among the polymers is the presence of a variable number of fluorine substituents on the BTZ moiety, absent for **P1**, 1 for **P2** and 2 for **P3**. The absorption of the polymers covers the range between 300 and 800 nm. Their stability was above 400 °C. The fluorine content progressively lowered the HOMO energy level for **P1**, **P2** and **P3** to −5.18 eV, −5.23 eV and −5.30 eV (calculated values), respectively. The number of fluorine atoms added to the BTZ counterparts increases the acceptor properties of the monomer, enhancing the difference with the BDT monomer and this was fruitful for the photovoltaic properties. The hole mobility was high (up to 2.01 × 10^−3^ cm^2^ V^−1^ s^−1^) and followed the same order, **P1** < **P2** < **P3**. The average PCE for the polymers **P1**–**P3** was 11.05%, 12.22%, 17.24%, respectively. The stability of the **P3**-based devices was evaluated over 30 days, in which it maintained a PCE of about 16.0%, with more than 92% of PCE retention.

The polymers **PBTBDT** and **asy-PBTBDT** contain the same structural motifs and were prepared by assembling a BDT and a BTZ moiety surrounded by two thiophenes. The polymers are made on the basis of a BDT bearing two 2-butyloctyloxy chains, and in **PBTBDT** the BTZ monomer contains two hexyloxy substituents, whereas in **asy-PBTBDT** an ethoxy and a decyloxy substituents, being thus asymmetric [128]. This asymmetry has an essential influence on the solubility of the polymers. The **PBTBDT** shows a reduced solubility in organic solvents, while **asy-PBTBDT** is highly soluble and easily processable. This asymmetric feature became further important based on the choice of 2-methylanisole (2-MA) as a deposition solvent, which is also certified for food use and also considered a green solvent. The solubility of the asymmetric polymer into this solvent was very high, while **PBTBDT** is not, leading to obtaining an optimized deposition of the HTM film. The hole mobility was high (1.34 × 10^−3^ cm^2^ V^−1^ s^−1^) and the HOMO energy level is at −5.36 eV, properly aligned with the perovskite. The PCE reached the 18.3% outstanding value in the pristine state and 20.0% in the doped state. This was one the best results ever obtained until now, and it opened the way to a greener production of PSCs, avoiding, in turn, the use of chlorinated solvents.

An exceptional result in terms of PCE was obtained again by Kim et al. with another BDT-BTZ based polymer, named **PTEG** [115]. They produced two polymers containing the same BDT monomer, bearing 2-(2-ethylhexyl)thienyl substituents, and a BTZ monomer, in which BTZ is further surrounded by two thiophenes. The difference between the two polymers is related to the two alkoxy substituents on the BTZ ring. One polymer brings two dodecyloxy chains, and the other, named **PTEG**, contains two triethylene glycol monomethyl ether chains (TEG). The **PTEG** showed a high proneness to give films with a face-on orientation on the perovskite surface, probably due to the presence of triethylene glycol chains, which avoid the backbone twisting and, at the same time, ensure good interaction with the perovskite surface due to the higher energy of the TEG chains with respect to the classic alkyl chains [115]. Moreover, the TEG chains gave **PTEG** higher solubility (39.87 mg/mL, in chlorobenzene) than the alkyl-substituted polymer (2.73 mg/mL), improving its processability and reducing the amount of solvent required for the deposition. The presence of the TEG chain helps also to obtain high hole mobility, and very good contact with the perovskite as proved by the higher surface energy of the TEG group. In a PSC with FTO/TiO_2_/m-TiO_2_/Cs-perovskite/HTL/Au structure, the **PTEG** gave a PCE of 18.6% while in a planar architecture (FTO/SnO_2_/Cs-perovskite/HTL/Au) the PCE was even higher (19.8%, a really outstanding value). Upon addition of tBP and LiTFSI, the PCE increased in the first case to 19.1% but decreased in the second case to 19.0%. Further papers showed that one of the more important features of the TEG group is to act as a complexing agent for Pb^2+^ and that this was beneficial since the TEG chain can interact directly with lead in the perovskite [116,142].

An evolution of the **PTEG** polymer was the topic of an extremely interesting paper from Lee et al. [116]. Differently from the original **PTEG**, the authors replaced the substituent on the BDT monomer, from the 5-(2-ethylhexyl)thiophene to the 2-ethylhexyloxy one. A few polymers were obtained, named **alkoxy-PC8** (M_n_ = 25 kDa), **thiophenyl-PTEG** (M_n_ = 57 kDa) and **alkoxy-PTEG** (M_n_ = 50 kDa). The use of green solvent alternative to chlorobenzene (CB) provided an improvement to both the efficiency and the sustainability of the deposition process. In particular, the 2-methyl anisole (MA) and the 3-methylcycloesanone (MC) were not able to dissolve well the original **PTEG** while they dissolved very well the **alkoxy-PTEG**. The films given by MA and MC were thinner than those produced by CB and, in particular, MA gave more uniform films. GIWAXS analysis showed a face-on arrangement at the surface for both **alkoxy-PC8** and **alkoxy-PTEG**, but the former was less soluble in MA and MC. The final PSCs were thus prepared with **alkoxy-PTEG** only. In planar PSC, with Cs_0.06_FA_0.78_MA_0.16_Pb_0.94_I_2.4_Br_0.48_ perovskite, the **alkoxy-PTEG** gave a 20.6% efficiency when deposited from CB, 21.2% from MA and 19.9% from MC. The long-term stability was assessed for 20 non-encapsulated devices at room temperature and 40–50% relative humidity for 30 days. The **alkoxy-PTEG** showed retention of 88% of PCE. Moreover, encapsulated devices in the same conditions fully saved the PCE. Thermal stability at 100 °C was excellent for 4 days (94% PCE retention) but it decreased at 29% retention after 30 days. However, this was due mainly to the perovskite thermal stability issues in the trial conditions more than to HTM.

Further modifications in the polymeric skeleton were recently exploited by using different acceptor groups other than BTZ.

The polymers **pBBTa-BDT1** and **pBBTa-BDT2** were prepared and used with success in PSCs [129]. The two moieties coupled to form the polymers were the 4,8-dithien-2-yl-benzo[1,2-d;4,5-d′]bistriazole with two hexyl chains and the benzo[1,2-b:4,5-b′]dithiophene bearing two 2-hexyldexyloxy chains. These polymers show a great interchain interaction and, besides, both form films with a face-on orientation on the deposition surface. The polymer **pBBTa-BDT2** is face-on oriented for a large extent, greatly helping the charge transfer, as shown by a higher vertical conductivity and the hole mobility. This could explain the difference in PCE (7.0% for **pBBTa-BDT1** and 14.5% for **pBBTa-BDT2)**. The devices were also tested for stability: **pBBTa-BDT2** retained about 30% of the initial PCE after 18h, while **spiro-OMeTAD**, employed as a reference, lost any activity in 5h [129].

The polymers **P1-0F**, **P2-2F** and **P3-4F** were prepared by coupling a BDT monomer containing phenylthiophene moieties with two 2-ethylhexyloxy chains and a thieno[3,4-c]pyrrole-4,6(5H)-dione central ring bearing one 2-ethylhexyl chain and surrounded by two thiophenes [130]. In these polymers, the degree of fluorination in the side chain benzene rings was tuned. The HOMO of the **P-0F**, **P2-2F** and **P3-4F** polymers (−5.41 eV, −5.43 eV and −5.50 eV, respectively) was tuned by the introduction of the fluorine atoms and the LUMO behaved accordingly (−3.59 eV, −3.61 eV and −3.68 eV). The fluorination degree seemed to alter the energy levels without altering the band gap (1.82 eV). The thermal stability showed to be higher than 400 °C for all the polymers. The dipole moment was higher for the fluorinated polymers with respect to the nonfluorinated one, **P1-0F** (1.44 D, 4.44 D and 2.75 D for **P1-0F**, **P2-2F** and **P3-4F** respectively), and this was also a measure of a higher molecular packing and order. In this paper, the **P1-0F**, **P2-2F** and **P3-4F** polymers showed hole mobility of 2.51 × 10^−5^, 4.06 × 10^−5^ and 9.32 × 10^−5^ cm^2^ V^−1^ s^−1^ respectively, while flash photolysis time-resolved microwave conductivity measurements showed that the hole transfer yield (from perovskite to the HTM) decreased according to the fluorination degree, 0.88, 0.80 and 0.54 for **P1-0F**, **P2-2F** and **P3-4F** respectively. The hole transfer yield (0.88% for P1-0F, 0.80% for P2-2F and 0.54% for P3-4F) as measured by flash photolysis and time-resolved microwave conductivity is mainly governed by thermodynamic factors, i.e., by the energy offset between the HTM HOMO and the perovskite valence band and thus the higher the HOMO energy level the higher the transfer yield. This helps to focus on the fact that the best PCE and, in general, the best performance is a balance between the different phenomena, such as charge separation, hole transfer and hole mobility. Into cells with an FTO/c-TiO_2_/m-TiO_2_/perovskite/HTM/Ag architecture, the PCEs obtained for **P1-0F**, **P2-2F** and **P3-4F** were 9.80%, 14.94% and 10.35%, with minimal hysteresis [130].

The **P-TT-TPD** copolymer was produced by Kranthiraja et al. coupling a donor unit (based on benzodithiophene, BDT) with an acceptor building block (composed by thieno[3,2-b]thiophene and 4H-thieno[3,4-c]pyrrole-4,6(5H)-dione) [131]. The structure is quite complex, but it closely resembles the already cited **P2-2F** [130], in which the thiophene spacer was substituted by a thieno[3,2-b]thiophene. The HOMO was estimated to lie at −5.46 eV and a reduced band-gap of 1.79 eV was found. For the monomer and dimer, the DFT calculations showed that the dipole moment is very high and this probably is inducing the building blocks to pack closely also in the polymer. The **P-TT-TPD** was used as dopant-free HTM onto a mesoporous (FAPbI_3_)_0.85_(MAPbBr_3_)_0.15_ based PSC and gave a PCE of 17.0%. The simple substitution of the spacer from thiophene to thieno[3,2-b]thiophene caused an efficiency increase of more than 3%. By using the tris(pentafluorophenyl)borane (BCF) as an additive, which enhances the quantity of induced holes in the polymer, the PCE of 17.56% was obtained. Remarkably, also this HTM allowed to obtain devices showing a minimal hysteresis.

A series of polymers based on BDT and BODIPY units was prepared by Kyeong and co-workers, **K1**–**K6** [132]. The modification of the alkyl chains on the BDT moiety and of the meso substituent on the BODIPY unit helped to tune HOMO and LUMO, respectively. Previous studies demonstrated that both the energy levels of molecular orbitals in the HTMs were changed simultaneously by chemical modifications of polymer structure [143,144,145]. Since the LUMO of the HTM should be ideally well higher in energy than the conduction band of the perovskite, to minimize the recombination probability at the perovskite/HTM interface (i.e., the back-transfer of an electron in the HTM from the PSK), chemical modification of polymers was applied to tune the energy levels. The connection between the BDT and BODIPY is away from planarity and this helps the HTM to better cover the perovskite. The HOMO and LUMO energy level range is in between −5.29 and −5.42 eVand −3.33 and −3.37 eV, respectively. The modification of HOMO is ascribable to the different alkyl chains on BDT moiety, whose effect is however quite limited. Those HTMs were used for the preparation of inverted p-i-n PSCs. The presence of a meso CF_3_ containing BODIPY deepens (too much) the LUMO energy level and, in turns, sensibly reduced the PCE, while the presence of a meso CH_3_ BODIPY lead to the opposite behaviour (more energetic LUMO and increased PCE increased up to 15.19% for **K1**, with the best cell showing 16.02%). Some perovskite ionic precursors were incompatible with the hydrophobic surface of the HTM and thus the polymers showed unwetting behavior towards perovskite; thus, the conjugated polyelectrolyte poly[(9,9-bis(3′-(*N*,*N*-dimethylamino)propyl)-2,7-fluorene)-alt-2,7-(9,9-dioctylfluorene)] (PFN) was used as a wetting conductive polymer to help the deposition of the **K1**-**K6** polymers. The PCBM was used as ETL layer and zirconium acetylacetonate as the cathode interface layer. **K1** reached the best PCE result since it has higher hole mobility (2.96 × 10^−5^ cm^2^ V^−1^ cm^−1^). Stability tests under open air, showed that **K1** based devices maintained 80% of the pristine PCE over 10 days [132].

A few different acceptor groups were coupled with BDT, the pyrrolo[3,4-c]pyrrole-1,3-dione (DPPD) and the 4H,8H-benzo[1,2-c:4,5-c’]dithiophene-4,8-dione (BDD).

Two polymers **R1** and **R2** were prepared by Rana and co-workers. **R2** was a typical D-A polymer, just the already cited **PTB-BO**, while **R1** was made of two D-A monomers [133]. In **R1**, the donor portion in both monomers was the 4,8-bis(5-(2-ethylhexyl)thiophen-2-yl)benzo[1,2-b:4,5-b′]dithiophene (BDTT), which was coupled either with pyrrolo[3,4-c]pyrrole-1,3-dione (DPPD) and [3-fluoro-2-[(2-ethylhexyl)carbonyl]thieno[3,4-b]thiophene] (TT) as the two acceptor units. **R2** is simply built on BDTT and TT. The HOMO levels lie at −5.42 eV and −5.37 eV for **R1** and **R2** respectively, nearly well aligned with the MAPbI_3_ perovskite’s valence band. The hole mobility was higher for **R1** (3.7 × 10^−3^ cm^2^ V^−1^ s^−1^) than for **R2** (5.8 × 10^−4^ cm^2^ V^−1^ s^−1^). The polymer **R1** showed the best efficiency in the PSCs, approaching 16%, while **R2** achieved 13.6%. The lower performance of **R2** was mainly ascribed to its shallower HOMO which reduces the V_oc._ Once more, it appears evident that the mixing of different D-A monomers can help to better tune the polymer characteristics, e.g., the hole mobility, improving its behavior towards efficiency. The PSCs were fully stable (they saved 100% of the original PCE) at 25 °C, 75% of relative humidity, for 500 h.The **PBT1-C** D-A copolymer was obtained from the copoylmerization of benzodithiophene (BDT) with 1,3-bis(4-(2-ethylhexyl)thiophen-2-yl)-5,7-bis(2-alkyl) benzo[1,2-c:4,5-c′]dithiophene-4,8-dione (BDD) [134]. This last moiety showed to be an efficient Lewis base that can work to passivate the surface traps, often occurring at the PSK surface due to uncoordinated Pb surface sites. This coupling led to a polymer whose hole mobility and properties were undoubtedly promising. When it was applied by spin coating onto perovskite, it mostly self-organized in a face-on way thus passivating the uncoordinated Pb^2+^ centers. On the other side, the preferred orientation of **PBT1-C** is in an edge-on way and gave smoother film than **P3HT**, used as a reference. On mesoporous PSCs **PBT1-C** showed a very high PCE (19.06%) while the **P3HT** reached a notably 14.96%, in its undoped state. As a final remark, the devices retained 80% of their PCE after storage at 25 °C under inert gas for 300 h.

Four BDT based polymers were obtained by coupling with benzo-[1,2-c:4,5-c0]dithiophene-4,8-dione (BDD), **PBDB-T**, **PBDB-T-2Cl**, **PBDB-T-2F**, **PBDB-T-Si**, that were very similar to **PBT1-C**. [135] The introduction of chlorine, fluorine atoms or an organosilyl substituent tried to modulate the properties. The molecular distance for the p–p stacking in the crystals diminishes in the order **PBDB-T** > **PBDB-T-2Cl** > **PBDB-T-2F** > **PBDB-T-Si**, and this is in agreement with the increase of the resistance to the recombination and with the PCE order.

Three new polymers (originally named P1, P2 and P3, here renamed **P1-B**, **P2-T** and **P3-T-F** respectively, to avoid misunderstanding and homonymy with other polymers reported in this review) were prepared by Zhang et al. exploiting a bis(2-EH-Th)BDT as the central core [136]. The latter was copolymerized with a phenyl (**P1-B**) or thiophene (**P2-T**) group. Moreover, as a slight but substantial modification, a fluorine atom was inserted on the lateral thiophenes on the BDT (**P3-T-F**). The thiophene spacer helped to planarize the polymeric skeleton, as demonstrated by DFT calculations and the appearance of a red-shifted shoulder in the UV spectra, which also accounts for promoted aggregation phenomena in solution. The different structures among the three polymers helped to finely tune the HOMO energy level: −5.16 eV for **P1-B**, −5.27 eV for **P2-T** and −5.38 eV for **P3-T-F**. GIWAXS study showed that **P1-B** organizes at the PSK’s surface in an edge-on way while both **P2-T** and **P3-T-F**, which are more planar, adopt a face-on orientation. The latter outperformed **P1-B** (PCE = 14.9%) in PSCs (FTO/mp-SnO_2_/MAPbI_3_/HTM/Au), leading to an efficiency of 19.6% (**P2-T**) and 20.3% (**P3-T-F**). This shows that the insertion of fluorine on the side chain portion of the BDT still improved the performance of the BDT-T polymer by just less than 1% (+4%). When the non-encapsulated PSCs were kept at 25 °C in 20–70% relative humidity conditions, those made with **P2-T** and **P3-T-F** HTMs survived for 40 days, maintaining 97% of their pristine PSC whereas **P1-B** based devices were not tested for stability. Very interestingly, the most efficient HTM (**P3-T-F**) was also implemented in a flexible PSC with a PET/ITO/mp-SnO_2_/MAPbI_3_/HTM/Au architecture, providing an excellent PCE: 16.2%, thus envisaging potential application in view of a “roll-to-roll” PSC production. This really elegant screening clarified that sometimes it is not mandatory to build an HTM by coupling “very strong” complex donor and acceptor monomers, but simpler structures can give excellent results and can greatly simplify the access to highly performant HTMs, reducing the cost and improve the sustainability of the material.

In a recent paper, the **P1-Th-BDT-BO**, **P2-OR-BDT-BO** and **P3-OR-BDT-T** BDT based polymers were prepared to exploit the sulfur–oxygen interaction in order to force a planarity on the main conjugated chain [137]. They showed good thermal stability with decomposition temperature over 300 °C. **P1-Th-BDT-BO** and **P2-OR-BDT-BO**, which only differ for the substitution on the central ring of BDT (thiophene vs. alkoxy substituent, respectively) showed remarkable differences for the HOMO energy level (−5.43 vs. -5.76 eV respectively). On the basis of the excellent efficiencies of **P1-Th-BDT-BO**, one can imagine that due to the bad alignment with the perovskite (−5.48 eV) used in this work, **P2-OR-BDT-BO** could be effectively employed with a deeper valence band perovskite. In the planar cell arrangement, **P1-Th-BDT-BO** gave a PCE of 18.30%, slightly lower than doped **spiro-OMeTAD**. At 20 °C and 80% of relative humidity, the **P1-Th-BDT-BO** based PSCs saved 89% of its pristine PCE after 96 h.

The BDT was copolymerized with thiophene and/or the 3,4-difluorothiophene to obtain some polymeric HTMs named **PBDT[2H]T**, **PBDT[2F]T** and **PBDT(T)[2F]T** [138]. The energy levels were around −5.29 eV and the hole mobility was not so high [138,139]. GIWAXS studies showed a high proneness to adopt a face-on arrangement at the surface for **PBDT[2F]T** and lower for **PBDT(T)[2F]T**, while no clear evidence was found for **PBDT[2H]T**. The face-on arrangement is the reason behind the overcoming of the low hole mobility since the **PBDT[2F]T** reached an efficiency of 17.25% in a planar PSC. It is noteworthy that the best performing polymer **PBDT[2F]T** was also the lightest one (28 vs. 48–49 kDa), which often is uncommon. When exposed to a 30% relative humidity and 30 °C the **PBDT[2F]T** cell retained 91% of PCE after 16 days.

##### 3.3.2.3. BTZ (Benzothiadiazole) and Cbz (Carbazole)

Often, the carbazole (Cbz) was used as a donor [146,147] and the benzothiadiazole (BTZ) was used as an acceptor in solar cells [11,148,149]. They were combined with other co-monomers with different approaches in PSCs. By limiting the discussion to the dopant-free topic and to 15% PCE cut-off limit, we can recall that several polymers were already shown in this review, containing benzothiadiazole (**P** [125], **RCP** [126], **P1**, **P2**, **P3** [127], **PBTBDT** [128], **asy-PBTBDT** [128], **PTEG** [115], **Alkoxy-PTEG**,[116], **Thiophenyl-PTEG** [116], **Alkoxy-PC8** [116]) and carbazole (**PPN** [89], **PVCz-OMeDAD** [90], **PVCz-OMeTPA** [91], **P1-PVCz-OMeTAD** [92], **P2-PVCz-OMeTAD** [92], **PVP-CZ** [93], **PCBTDPP** [77]).

Those benzothiadiazole (BTZ) based polymers achieved excellent photovoltaic efficiencies, up to 21.2% for the **Alkoxy-PTEG** polymer [116]. This comes out from a series of improvements, such as the combination of donors and acceptors, modification of BDT with alkoxy chains or alkyl thienyl moieties, insertion on the BTZ residue of the triethylene glycol chains as passivator for Pb^2+^ surface traps. The BTZ acceptor was thus tuned in its acceptor strength due to the alkoxy/TEG chains and this contributed to the optimization of the photovoltaic performances.

**Scheme 6:**
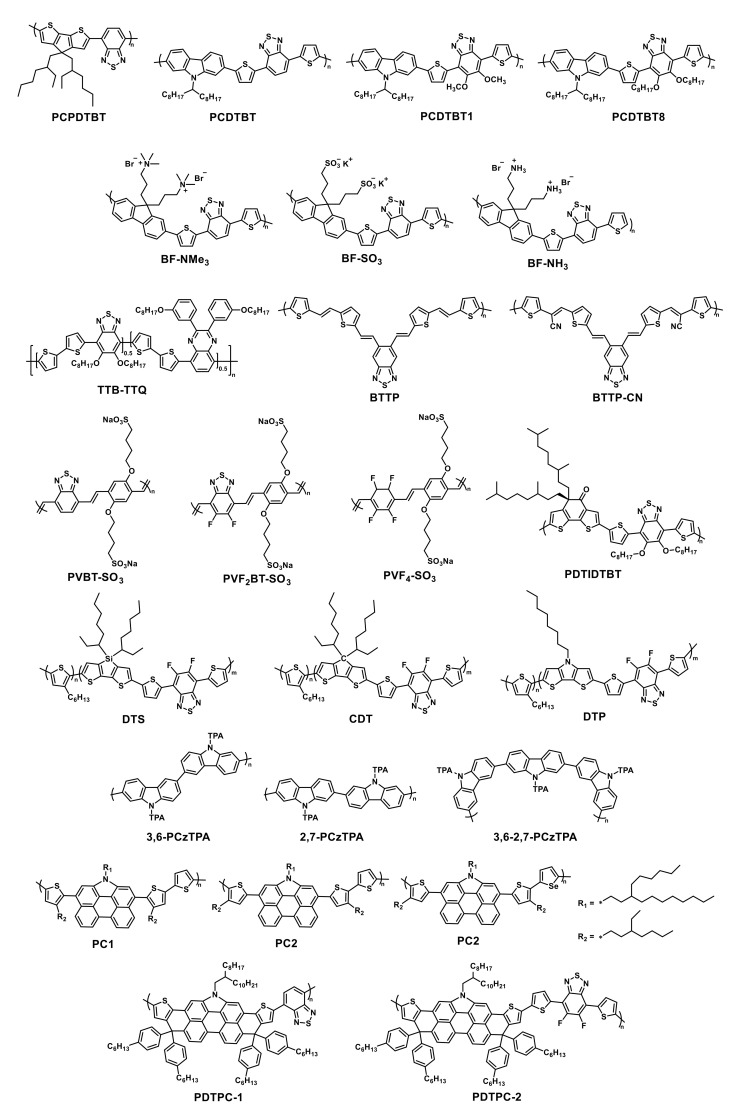
HTMs containing the benzothiadiazole (BTZ) and or the Carbazole (Cbz) group.

**Figure 4:**
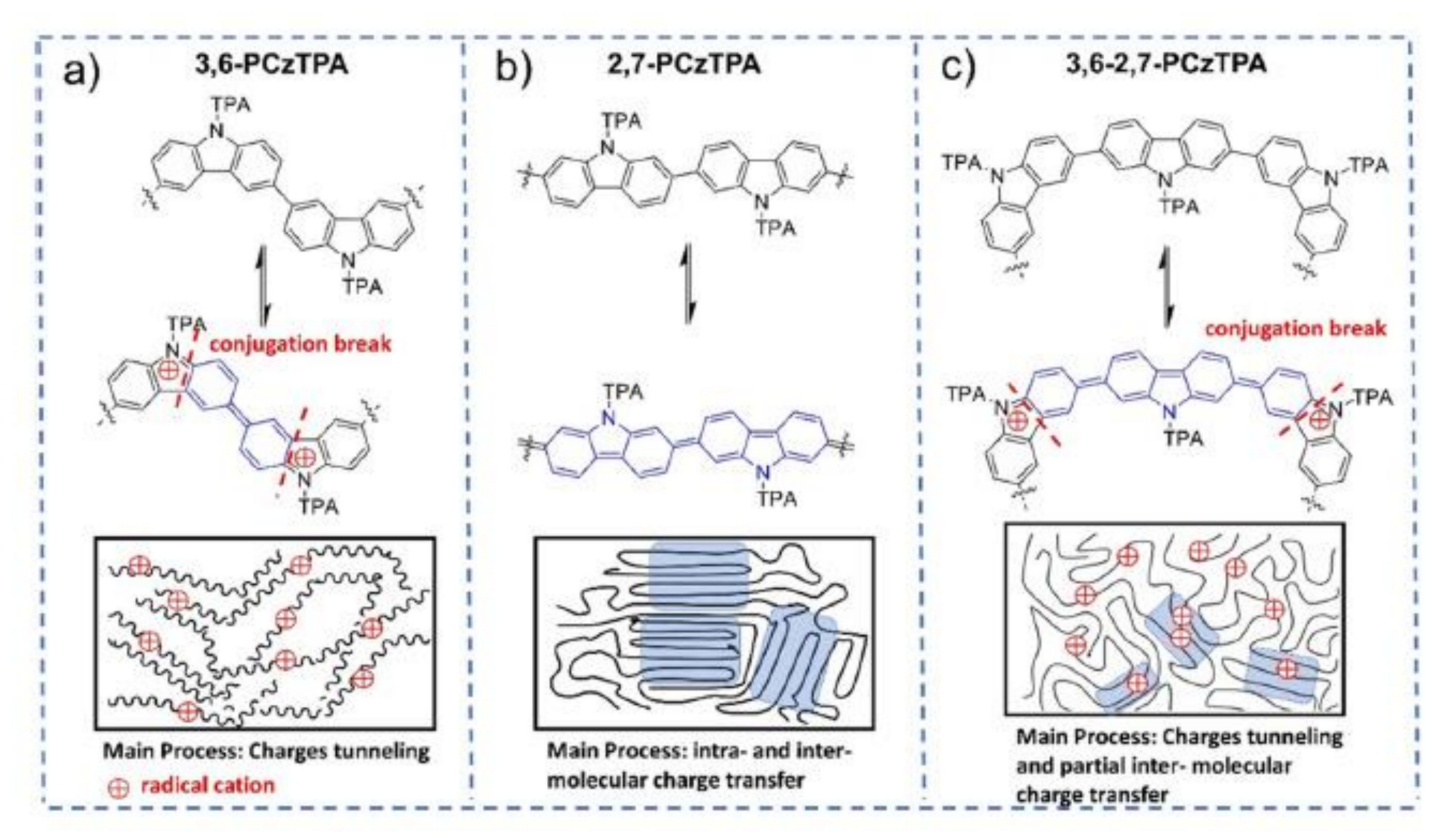
Possibilities of Quinoid States for Three Polycarbazoles and Their Main Charge Transport Processes. Reprinted with permission from (Xie, Y.et al. Macromolecules 2019, 52 (12), 4757–4764) [158]. Copyright (2019) American Chemical Society.

Interestingly, carbazole and benzothiadiazole were coupled together to give the **PCDTBT** family of polymers (for all benzothiadiazole-based and carbazole-based polymers please see Table 4, Figure 4 and Scheme 6).

A few polymers were used to modify the surface of an ITO/PEDOT:PSS interface. Among **PCDTBT**, **PCPDTBT**, **P3HT** and **DPP-DTT**, the **PCDTBT** polymer outperformed the others [99]. Since the perovskite was grown on a very thin polymer layer, it was demonstrated that the more crystalline perovskite was obtained with **PCDTBT**, and the higher PCE performances (up to 16.5%) were ascribable to this feature [99]. However, since the tunneling distance for hole transport is larger than the polymer layer thickness, the polymer did not mediate the hole transport. This was a different condition with respect to most PSCs studied until now.

The effect on the alkylation of the benzothiadiazole moiety in the **PCDTBT** polymers was studied in different papers. The polymers **PCDTBT1** and **PCDTBT8** were synthetized by inserting methoxy and octyloxy substituents on the BTZ scaffold [150]. The HOMO energy levels were −5.35 eV, −5.38 eV and −5.40 eV for **PCDTBT**, **PCDTBT1** and **PCDTBT8**, respectively. The **PCDTBT** was already shown to easily pack with π–π stacking and adopting a face-on orientation towards the perovskite surface [151]. This stacking is severely modified by the presence of further chains like the alkoxy one, which can compromise the coplanarity of rings. This effect was less severe for the very short methoxy group. Based on this effect, the **PCDTBT1** showed a PCE improvement with respect to **PCDTBT** while the **PCDTBT8** long chains gave a hindrance that negatively affected the PCE. This evidences that the ability to transport charges is not the only parameter determining the device’s efficiency. The **PCDTBT1** showed an excellently fast charge transport with respect to the other two polymers (proved by the very low dark current). The PCE were as follows: 16.43% for **PCDTBT** (best cell: 17.1%), 18.67% for **PCDTBT1** (best cell: 19.1%) and 14.81% for **PCDTBT8** (best cell: 15.4%) [150].

Three conjugated polyelectrolytes, **BF-NMe_3_**, **BF SO_3_** and **BF-NH_3_** were prepared on the basis of a fluorene monomer, which contained two electrolyte moieties and a bis(thieno)thiazole (4,7-di(thiophen-2-yl)benzo[c][1,2,5]thiadiazole) monomer by the Stille method [152]. These HTMs are very similar to **PCDTBT8**, **PCDTBT1 and PCDTBT8** and were applied in inverted PSCs with MAPbI_3_ perovskite. With respect to **PEDOT:PSS** they improved the preparation of the perovskite, obtaining larger grains (in the order **BF-NMe_3_** < **BF SO_3_** < **BF-NH_3_**) and the related charge mobility, which was reflected onto the final J_sc_ in the PSCs. Moreover, the recombination resistance showed the reverse order. The PCE obtained in inverted PSCs based on the MAPbI_3_ perovskite were 14.92%, 17.14% and 17.71% for **BF-NMe_3_**, **BF SO_3_** and **BF-NH_3_** respectively.

The BTZ core was also used in a D-π-A polymer, **TTB-TTQ**, that was prepared by assembling two monomers, TTB and TTQ, in which two thiophene rings are coupled with either alkoxylated benzothiadiazole (BTZ) or Quinoxaline (Q) [153]. Since the homopolymer of TTB is highly crystalline and gives bad and inefficient HTM films on perovskite, the incorporation of TTQ broke the molecular symmetry, reducing the π–π strong interactions, and the resulting polymer showed an excellent solubility in organic solvents and reasonable (6.2%) to good (14.1%) performance in PSC in its pristine and doped state, respectively. While both homopolymers obtained for TTB and TTQ have too deep HOMOs, to be correctly aligned with perovskite valence band level, the copolymer possesses a more energetic HOMO that matches well the perovskite energy level. Very interestingly, the hole mobility of **TTB-TTQ** in its pristine state was close to that of **spiro-OMeTAD**.

As a further example of BTZ based polymers, the **BTTP** and **BTTP-CN** polymers were prepared by Wittig reaction of a benzothiadiazole diphosphonate monomer and a dialdehyde (1,2-bis(2-formylthienyl)ethylene) monomer [154]. **BTTP-CN** contained a cyano group. The HOMO energy levels were −5.40 and −5.25 eV respectively, while the LUMO levels were −3.65 eV and −3.5 eV. Since the MAPbI_3_ perovskite’s conduction band is lying at −3.9 eV, those LUMO levels do not block efficiently the back electron transfer and this should negatively impact the efficiency. Indeed, the PCE were 3.80% for **BTTP** and 3.48% for **BTTP-C**, while the **spiro-OMeTAD** gave 7.55% and 12.53% in its undoped and doped state, respectively.

Three conjugated polyelectrolytes were prepared on a skeleton made by BTZ and a p-dialkoxybenzene, where the alkyl chains contain the salt moieties [155]. The **PVBT-SO_3_**, **PVF2BT-SO_3_** and **PVF4-SO_3_** polymers were used to explore and understand the hole transfer in inverted PSCs. The difference in the aryl vinylene component helped to modulate the HOMO level. In the inverted PSCs based on MAPbI_3_, the PCE of those HTMs was 17.57% 13.16% and 6.69% (with champion cells exhibiting values as high as 18.4%, 13.6% and 7.3%), for **PVBT-SO_3_**, **PVF2BT-SO_3_** and **PVF4-SO_3_**, respectively, which was consistent with the HOMO level and the alignment with perovskite valence band.

The **PDTIDTBT** polymer was described by Tavakoli et al. and used in mesoporous PSCs [156]. The stannyl derivative of dithienylbenzodthiadiazole was coupled with the 2,7-dibromo-(5,5-bis(30,50-dimethyloctyl)-5H-1,8-dithia-as-indacenone) by the Stille protocol. The production of dithiaindacenone derivatives was attractive from the economic point of view and was environmentally attractive, with E factor values of lower than 400. The **PDTIDTBT** HOMO was found at −5.35 eV and showed good hole mobility, higher than **spiro-OMeTAD**. The thermal stability was very good, up to 324 °C and the fluorescence measurements on the perovskite/**PDTIDTBT** film showed a very strong quenching, accounting for an excellent hole transfer and transport. The PCE of **PDTIDTBT** on a mesoporous cell based on a CsFAMA perovskite was 17.9%. The introduction of a very thin layer of poly(methyl methacrylate), PMMA, which is an insulating material helped to reduce the interfacial recombination of electrons from the perovskite with the hole in the HOMO of the HTM. This results in an increase in PCE, up to 19.89%. Excellent stability was found since more than 93% of the pristine PCE was maintained for 42 days at 42% of relative humidity and room temperature.

Three complex polymers were prepared by Ma et al. by starting from a **P3HT** skeleton which was further polymerized to include a BTZ monomer and either 4,4-bis(2-ethylhexyl)-4H-silolo[3,2-b:4,5-b’]dithiophene (obtaining **DTS**), 4,4-bis(2-ethylhexyl)-4H-cyclopenta[2,1-b:3,4-b’]dithiophene (obtaining **CDT**), or 4-n-octyldithieno[3,2-b:2′,3′-d]pyrrole (obtaining **DTP**)[157]. The **P3HT** was preliminary prepared as a reasonably high regioregular polymer by the KCTP/GRIM method which maintained a Br atom at one end [24]. The synthesis of the final polymer was performed by the Stille reaction involving the 5,6-difluoro-4,7-bis(5-(trimethylstannyl)thiophen-2-yl)benzo[c][1,2,5]thiadiazole the brominated **P3HT** and either the brominated silolodithiophene, cyclopentadithiophene and dithienopyrrole derivatives. The thermal stability was at least 200 °C showing that the polymers should survive to the conditions used for PSC preparation. The HOMO energy levels were modulated following the order **DTP** > **CDT** > **DTS**. The PCE for dopant-free **DTS**, **CDT** and **DTP** in inverted PSC with MAPbI_3_(Cl) perovskite were 17.27%, 17.02% and 14.87%, following the hole mobility order. When the zwitterionic polymer poly(sulfobetaine methacrylate) (PSBMA) was used to better match the wettability of the HTM with the perovskite, the interface was substantially better and free from pinholes. After the deposition of the perovskite, the devices showed a very good performance, overcoming 20%: 20.16%, 20.05% and 17.60% for **DTS**, **CDT** and **DTP**, respectively.

In this review, carbazole HTM polymers were mainly reported as side-chain vinyl polymers, namely **PVCz-OMeDAD** [90], **P1-PVCz-OMeTAD** [92], **P2-PVCz-OMeTAD** [92], with PCEs over 16% and **P2-PVCz-OMeTAD** reaching 18.4%. This series showed that the approach to the polymeric HTMs can be simplified by producing small molecule monomers, whose polymerization can give optimized polymers. Moreover, the vinyl polymerization processes are well known and can be finely tuned to obtain the material with the desired performance. This is an excellent starting point, towards the industrial production of HTMs (and devices).

Polycarbazoles were studied to use as HTMs in PSCs by Xie and co-workers [158]. They synthetized three polycarbazoles based on the N-TPA-carbazole which was polymerized with the Stille protocol obtaining various polymers by connecting the units at different linking positions, **3,6-PCz-TPA**, **2,7-PCz-TPA** and **3,6-2,7-PCz-TPA**. The latter behaves like a copolymer with perfect alternation of carbazoles which are linked at 2,7- and 3,6- positions. The HOMO energy for **3,6-PCz-TPA**, **2,7-PCz-TPA** and **3,6-2,7-PCz-TPA** were −5.18eV, −5.23 eV, −5.22 eV in solution while they were -5.22 eV, −5.37 eV and 5.34 eV in solid films. This demonstrated the urge for measuring the energy levels both in solution and in solid-state, to better comprehend the behavior of HTMs in PSCs. Normally, the charge transport in conjugated polymers is mainly determined by (i) intramolecular conducting along the polymer main chain, which can be found for **2,7-PCzTPA**, (ii) intermolecular charge hopping across chains owing to π−π stacking, and (iii) charge tunneling between conducting units separated by less conducting segments, usually observed in doped polymers, to which one can ascribe the conductivity of **3,6-PCz-TPA** [158,161] (See Figure 4). The electric impedance study and also the following PSC characterization demonstrated that the two processes (i) and (iii) are the most probable to be involved in the case of **3,6-2,7-PCs-TPA**. In fact, in **2,7-PCzTPA**, the charge probably migrates effectively through the polymer backbone, while the conjugation breaking appearing when the 3,6-link is present can help the charge to be transferred by the tunneling process. Cyclic voltammetry (CV) measurements showed the **3,6-PCzTPA** and **3,6-2,7-PCs-TPA** to give reversible oxidation waves, which accounts for the formation of radical cations. While the better delocalization of the quinonoid structure of **2,7-PCs-TPA** can help the charge transfer, probably it confines the charge into the polymer backbone and the conjugation breaking give chances for the tunneling process to operate, thus improving the hole mobility in the solid. Very interestingly, these polymers were fully transparent, and this makes them ideal for inverted PSCs (where the HTM should not show parasitic light absorption), leading to efficiencies as high as 16.4%, 17.1% and 18.4% for **3,6-PCz-TPA**, **2,7-PCz-TPA** and **3,6-2,7-PCz-TPA**, respectively. While these results are a bit lower than the actual record of 20–21% for inverted PSCs, it is noteworthy that these HTMs can be produced by acting on only one monomer, which makes them more affordable than most copolymers.

More complex scaffolds containing the carbazole were introduced in HTMs. Here we will only report on the last introduced scaffold: phenanthrocarbazole (PC) and dithienopicenocarbazole (DTPC). Three polymers were derived from a more complex scaffold based on carbazole. The polymers, **PC1**, **PC2** and **PC3** were built onto the N-(2-hexyldecyl)phenanthrocarbazole scaffold, bearing different substituents [159]. **PC1** contains at each side two 4-(2-ethylhexyl)-2,2′-bithiophene moieties, **PC2** a 3-(2-ethylhexyl)-2,2’-bithiophene and **PC3** a 3-(2-ethylhexyl)-2-(selenophen-2-yl)thiophene. The difference between **PC1** and **PC2** is related to the position of the 2-ethylhexyl chain on the thiophene directly linked to the phenanthrocarbazole. In **PC1** the chain points toward the scaffold system and in **PC2** it points away from it. **PC3** is an evolution of **PC2** in which the terminal thiophene is substituted by selenophene. The positional change of the chain modifies the steric requirements giving the **PC2** HTMs more chances to achieve planarity than in **PC1**, which facilitates the p–p stacking. This aspect was also confirmed by theoretical calculations and by a redshift into the absorption spectra of **PC2** and **PC3** with respect to **PC1**. The substitution of sulfur with selenium in the external heteroaromatic ring, improves the interaction with perovskite surface Pb, and due to its larger polarizability, it should improve the hole mobility. The synthetic path involved several steps, mainly based on Suzuki and Stille coupling. **PC1** showed edge-on arrangement onto the perovskite, higher molecular distance in the solid-state film and lower hole mobility, while **PC2** and **PC3** arranged face-on onto the perovskite and were packed more densely in the solid-state, achieving also higher hole mobility. The HOMOs energy levels were well aligned with perovskite ones and the thermal stability was excellent. All the previously discussed details were confirmed by the PCE obtained in mesoporous SnO_2_ cells, where **PC1** showed a PCE of 8.8%, **PC2** 18.3% and **PC3** 20.8%. The stability for the **PC3** cells tested in ambient air and under illumination at RT showed complete retention of PCE.

The dithienopicenocarbazole is a further highly planar and complex scaffold containing carbazole. Two polymers, **PDTPC-1** and **PDTPC-2**, were obtained by coupling them with the benzothiadiazole and the dithienylbenzothiadiazole [160]. Those HTMs showed high thermal stability (over 380 °C) and lower-lying HOMO for **PDTPC-1**, which accounted for higher photovoltaic efficiency since it matched better the perovskite valence band. This HTM also showed higher hole mobility. As a final result, **PDTPC-1** showed a higher hole extraction ability which enhanced both the V_oc_ and the fill factor, giving a PCE of 16.96%, while **PDTPC-2** only achieved 12.45%.

##### 3.3.2.4. Other Polymers

**Scheme 7:**
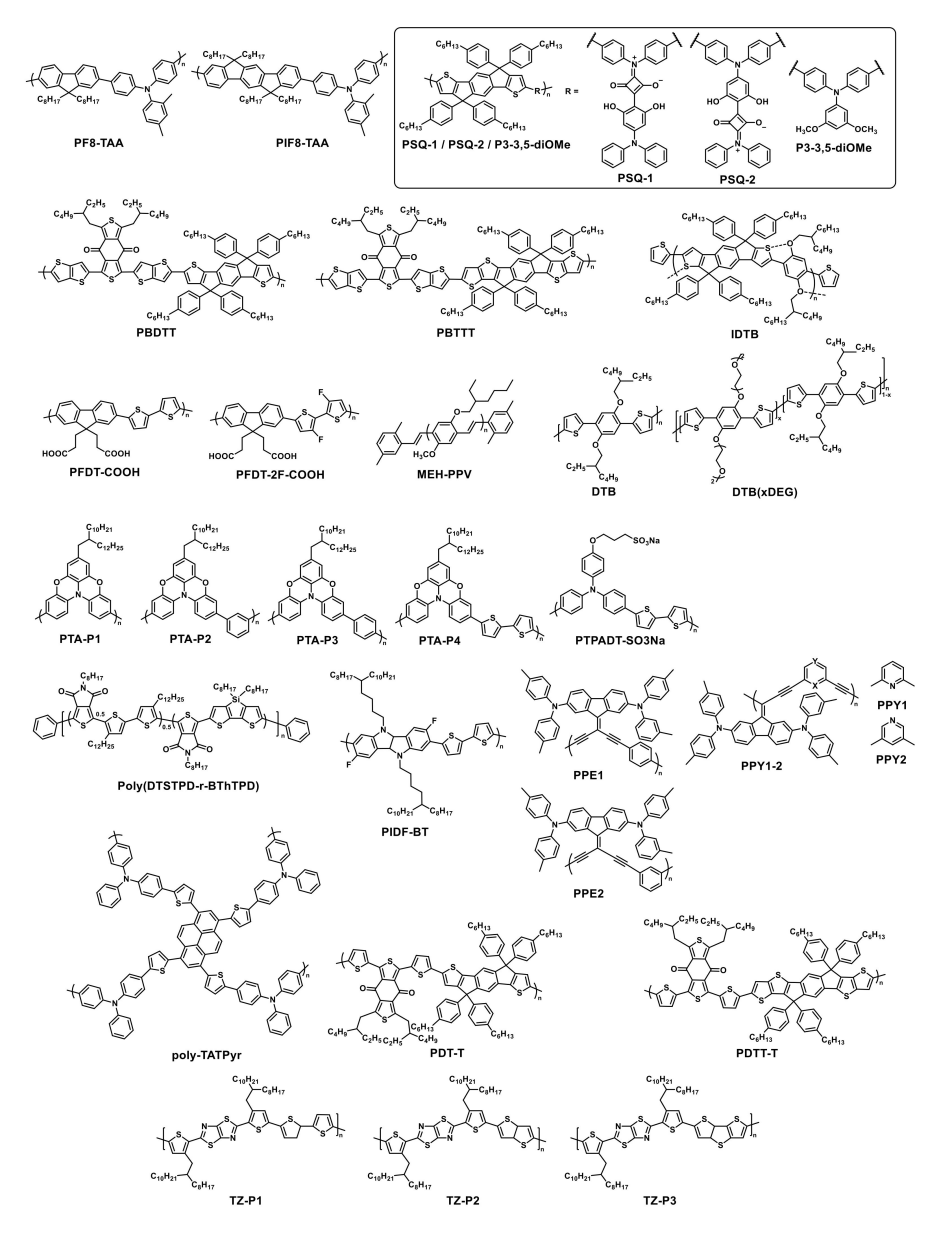

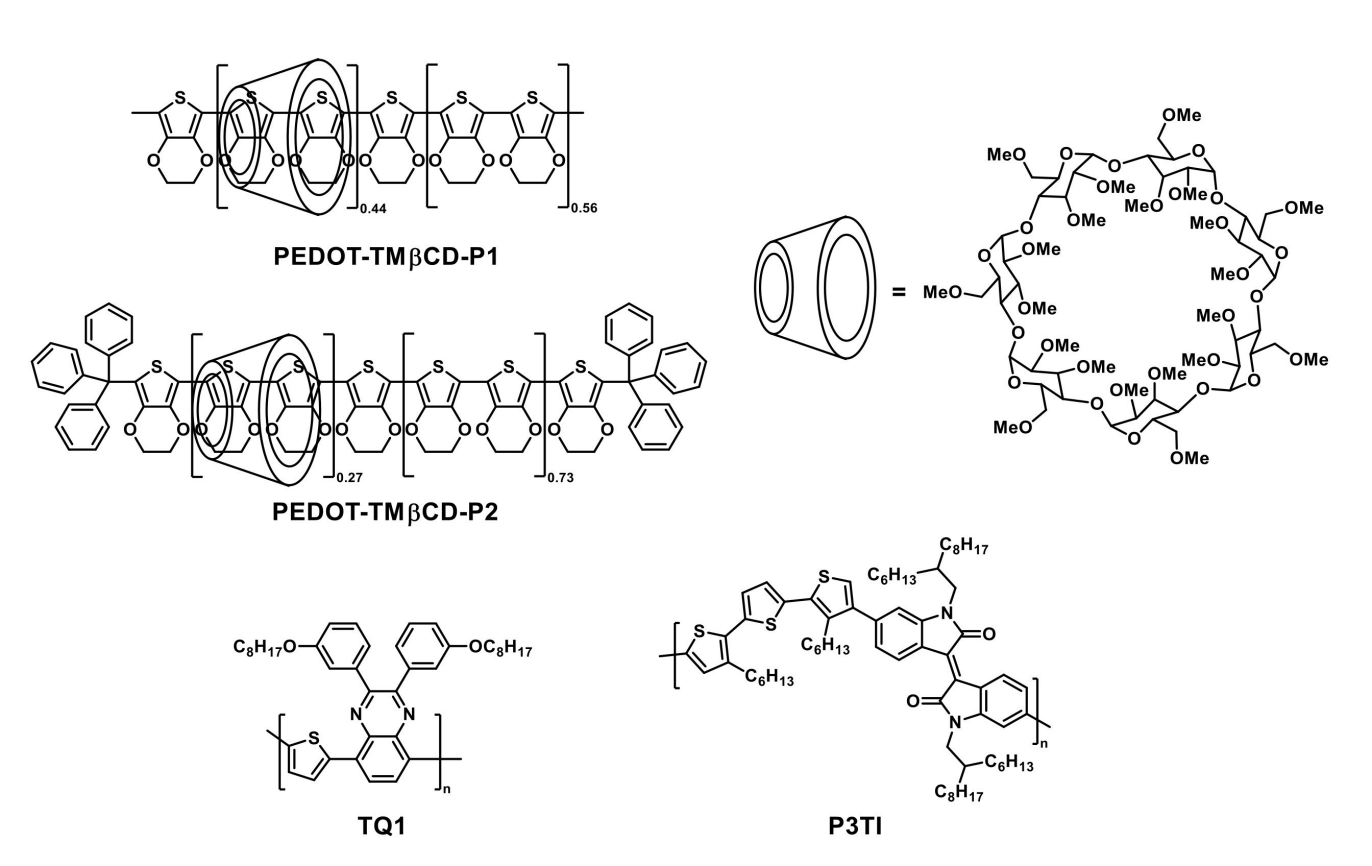
Miscellaneous of dopant-free HTM polymers.

In the literature, one can find some other HTM polymers that cannot completely be considered as belonging to the previously reported chapters (for all not specifically classified polymer please see Table 5 and Scheme 7). Some of them pertain mainly to the class of polytriaylamines, some others to benzothiadiazole and some can be considered as simplification of already shown more complex polymers. In some cases, the photovoltaic efficiencies were low but sometimes they got results of absolute relevance. This means that, far to be concluded, the research on the HTMs is still highly promising of relevant results and improvements. Here we will give some description of these HTM, trying to avoid it being a mere list of results.

Some polytriarylamine polymers were synthezsized based on anilines connected by fluorene, diphenylfluorene or indacenodithiophene/indacenodithieno[3,2-b]thiophene spacers, with some excellent remarkable results.

Polytriarylamine polymers based on the 2,4-dimethylanilino moiety were used in conjunction with both MAPbI_3_ and MAPbBr_3_. The three PTAApolymers contained different linkers: a biphenyl (**PTAA**), a diphenylfluorene (**PF8-TAA**) and a diphenyl fluorene-fluorene **PIF8-TAA** [162]. The hole mobilities for **PIF8-TAA** (0.04 cm^2^ V^−1^ s^−1^) are higher than **PF8-TAA** (0.02 cm^2^ V^−1^ s^−1^) and **PTAA** (4 × 10^−3^ cm^2^ V^−1^ s^−1^). The **PIF8-TAA** gave the highest PCE ever obtained with the MAPbBr_3_ perovskite (6.7%), due to its better-aligned HOMO, which effectively matches with the perovskite valence band, giving also a higher V_oc_. Moreover, the **PIF8-TAA** also shows a PCE of 9.1% with the MAPbI_3_ perovskite. It is worth noting that the HOMO of **PIF8-TAA** is lower than MAPbI_3_ perovskite valence band and so it should constitute a barrier towards the hole injection. The J-V plot shows a “roll-over” effect which could not be fully explained yet by the authors.

A series of three polymers was prepared by Xiao et al., based on an Indacenodithiophene (IDT) and an asymmetric squaraine (DPASQ) or the N,N-diphenyl-3,5-dimethoxyaniline, obtaining **PSQ1**, **PSQ2** and **P3-3,5-diOMe** [163]. The IDT co-monomer was chosen since it can give high intermolecular interactions as occurring in Organic Solar Cells. The copolymerization with the DPASQ moiety, occurring closer (**PSQ1**) or farther (**PSQ2**) from the cyclobutenedione ring, gave the title compounds. The HOMO energy levels were in the range −5.2/−5.3 eV, while the hole mobility was good for **PSQ1**, very good for **PSQ2** and well lower for **P3-3,5-diOMe**. The interaction of the ketone of the cyclobutenedione with Pb^2+^ ion was studied and it was assessed that all the three polymers can interact with the PSK and thus passivate the Pb^2+^ exposed sites, giving smooth and homogeneous films. In planar PSCs based on a-CsPbI_2_Br perovskite, these polymers showed efficiencies as high as 13.6% for **PSQ1**, 15.5% for **PSQ2** and 11.6% for **P3-3,5-diOMe**, when **spiro-OMeTAD** powered a PCE of 14.4%. The results were specifically interesting because, at the time of the paper (i.e., 2019), they approached the record PCE for similar fully inorganic perovskite (α-CsPbI_2_Br) that was 17.1% [164]. The stability under continuous illumination was monitored only for **PSQ2**, maintaining 83% of the PCE after 300 h.

The same scaffold, Indacenodithiophene (IDT) and its analog, indacenodithieno[3,2-b]thiophene (IDTT) were used by You et al. to synthesize novel polymers, **PBDTT** and **PBTTT**, in which they were coupled to bis(2-ethylhexyl)benzo[1,2-c:4,5-c′]dithiophene-4,8-dione (BDD), using a thienothiophene as a spacer [165]. The IDT or IDTT are playing the donor role while BDD is the acceptor. The two polymers showed similar M_n_ (13 and 14 kDa) and quite large PDI, (2.30–4.36) for **PBDTT** and **PBTTT**, respectively, and were thermally stable over 400 °C. The HOMO energy levels matched quite well the perovskite valence band. By DFT calculation, the structures of the dimers appeared almost planar, demonstrating a high ability to promote stacking. The hole mobility of those polymers was well higher than that of **P3HT**, probably due to the higher conjugation caused by fused rings and the quinoid structure. Contact angle measurements showed that they were more hydrophobic than **spiro-OMeTAD** and thus they were acting to better protect the perovskite layer. In the planar PSC devices, those HTMs were implemented as thinner layers (50–55 nm) than **spiro-OMeTAD** (around 200 nm), giving also excellent PCE: 20.28% for **PBDTT** and 19.48% for **PBTTT**, while the **spiro-OMeTAD** achieved a lower value (18.32%). The devices built with **PBDTT** and **PBTTT** were also reasonably stable, by maintaining 80% for their pristine PCE after 720 h of storage at 30% relative humidity at room temperature.

Additionally, the **IDTB** polymer was built on the indaceno[1,2-b:5,6-b’]dithiophene scaffold, but coupled with the bis(alkoxybenzene) co-monomers [166]. The polymer had a HOMO energy level at −5.20 eV and hole mobility slightly lower than **spiro-OMeTAD**. The proneness to p–p stacking was demonstrated by the UV spectra in the solid-state, while the XPS showed that S-O interaction between the sulphur of the indacenothiophene and the oxygen of the alkoxy groups improved the planarity of the polymeric skeleton. The **IDTB** HTM was used in PSCs (FTO/c-TiO_2_/SnO_2_/Cs_0.05_FA_0.8_MA_0.15_PbI_2.55_Br_0.45_/HTM/Au) showed a PCE of 19.38%, overcoming the **spiro-OMeTAD** (18.22%). Hysteresis was shown by **spiro-OMeTAD**, but not by **IDTB**. The stability was assessed over 60 days at 30 °C and 30–40% of relative humidity.

The fluorene core, with pendant chains, terminated by carboxylic groups was used as a spacer in novel polymers, which was coupled with a bithiophene and a fluorinated bithiophene. The two polymers **PFDT-COOH** and **PFDT-2F-COOH** were prepared easily, showed that their HOMO energy levels were lowered by the fluorination and attained excellent efficiencies in inverted FA_1-x_MA_x_PbI_3_ PSCs, reaching 20.64% and 21.68% for **PFDT-COOH** and **PFDT-2F-COOH**, respectively [167]. These impressive results can be the results of well-ordered and pinhole-free perovskite films grown on those HTM layers. The stability at 80 °C, under dark in N_2_, was measured at 720 h, revealing that those HTMs retained 80% of their PCE. Once more, this showed that more should be known about HTMs and that the D-p-A, the 2D-star shaped, the triphenylamine paradigms are only a few of the possible conditions that promote an efficient hole transport. A bright future for this research field still has to come and the unexplored space is in front of us. The **MEH-PPV** polymer was employed in the preparation of PSC showing an effect of the solvent used for the polymer deposition on the PCE. The use of chlorobenzene, dichlorobenzene and toluene slightly improved the PCE in the same order: 7.21%, 8.06% and 8.87%, while **P3HT** showed a PCE of 6.74%. By improving the PSC preparation procedure (TiO_2_ treatment with TiCl_4_, one-step preparation of perovskite) the PCE of **MEH-PPV** approached 10% [168].

The polymer **DTB** was prepared by polymerization of the monomeric 1,4-dibromo-2,5-bis((2-ethylhexyl)oxy)benzene with the 5,5’-bis(trimethylstannyl)-2,2’-bithiophene, in a Stille coupling reaction.

This polymeric skeleton can be considered as a great simplification of the polymeric skeleton seen above (Section 3.3.2.2) for **RCP** [126], **asy-PBTBDT** [128] and **PTEG** [115]. This “simplified” polymer maintained substantially the excellent results of its parent polymers but with a sensibly lower synthetic effort: indeed, only two steps were needed to accomplish the final synthesis of **DTB**. This polymer showed lower hole mobility than **spiro-OMeTAD**, a HOMO energy level lying at −4.96 eV and an edge-on orientation of its film on PSK, as demonstrated GIWAXS experiments. Albeit this last feature does not help to increase the hole mobility in the vertical direction, the PCE in FAMA perovskite cells was excellent, 19.19% (best value 19.68%), with a certified value of 19.5%, showing again that dopant-free polymers have enormous potential. [169] During stability tests performed at room temperature and 20% relative humidity, **DTB** retained 86% PCE over 30 days. The easy synthesis, high efficiency and good stability promote this kind of structure for further studies and large-scale application.

**DTB** was also used with the inorganic CuSCN HTM, attaining 22.0% of PCE [170]. The stability at room temperature was checked at 60% of relative humidity for non-encapsulated cells, which survived for 1000h maintaining more than 90% of the pristine PCE.

**Figure 5:**
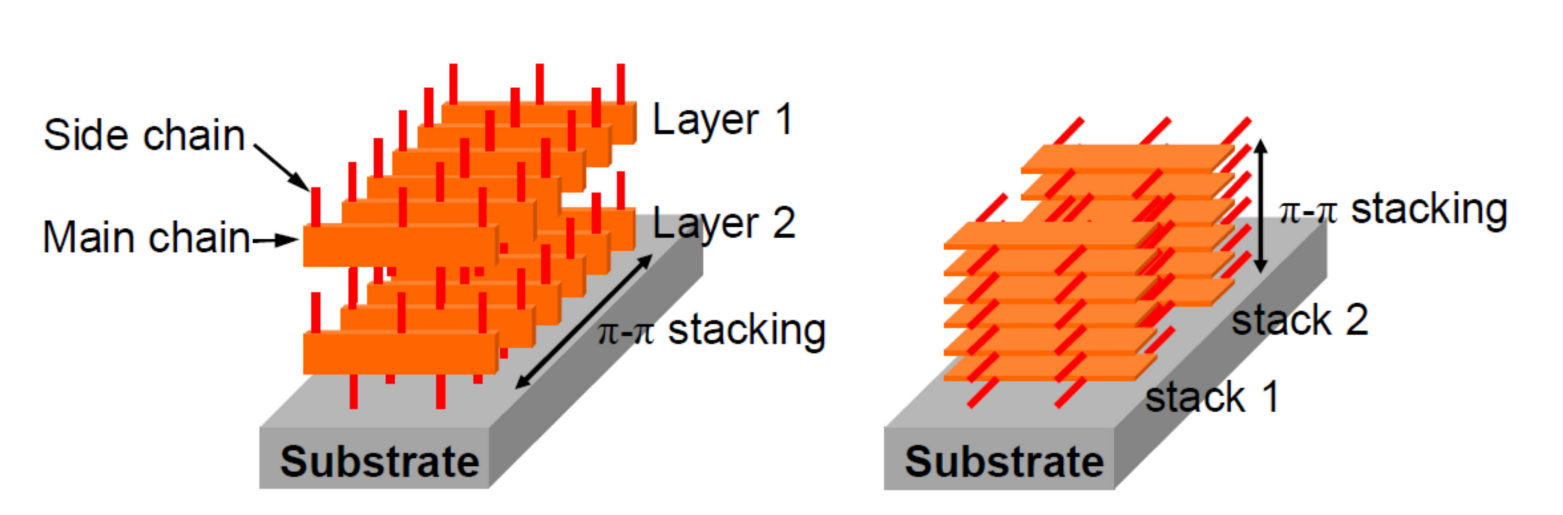
Different packing of polymers: edge-on (**left**); face-on (**right**). Reprinted with permission from ref. [171] Copyright 2015 John Wiley and Sons.

As further improvements starting from the DTB, the **DTB(xDEG)** polymers were prepared by copolymerizing a small fraction of the PhDEG monomer [171]. This is an easy approach to passivate the uncoordinated Pb^2+^ surface traps, due to the presence of the diethylene glycol chain whereas the HOMO level was just slightly upshifted. The GIWAXS measurements showed that the introduction of 3% of PhDEG did not modify the molecular packing but increased the crystallinity considerably, in both edge-on and face-on orientations (Figure 5). When the content of PhDEG was brought to 10% the crystallinity was decreased in both orientations, loosening lamellar and p–p stacking. As a result, both the hole mobility and the PCE followed the crystallinity degree. In mesoporous PSCs, with Cs_0.05_FA_0.81_MA_0.14_PbI_2.55_Br_0.45_, the remarkable efficiencies of 19.65% for the **DTB(0%DEG)** polymer and 20.19% for the **DTB(3%DEG)** were obtained.

Four polymers, namely **PTA-P1**, **PTA-P2**, **PTA-P3** and **PTA-P4,** derived from an oxygen bridged triphenylamine were prepared and tested in mesoporous PSCs based on perovskite [172]. These materials showed high thermal stability (over 400 °C) but only **PTA-P1** and **PTA-P4** showed reasonably high hole mobility and this was also reflected onto the final photovoltaic behavior. When doped, their PCE was in the range 4.9–7.6% with **PTAA** at 17.6%, but when in the undoped state the PCE was 12.1%, 1.3%, 9.1% and 11.1% respectively, with **PTAA** attaining only 4.8%.

The conjugated polyelectrolyte **PTPADT-SO3Na** was prepared by assembling a bisthiophene with a triphenylamine moiety, to which a sulphonic residue was tethered [173]. This material showed a conductivity of 2.68 × 10^−1^ S cm^−1^ conductivity and it gave smooth and homogenous film when deposited onto ITO, which was essential for potential use in inverted PSC. The photovoltaic behavior was excellent, giving a PCE of 18.15%, about 30% higher than the PEDOT:PSS (13.86%). Stability tests showed that **PTPADT-SO3Na** maintained 81% of the initial PCE for not-encapsulated cells at 50% of relative humidity and rt, while in the same conditions they saved 79% of the pristine PCE after heating at 85% for 200 h.

The **Poly(DTSTPD-r-BThTPD)** polymer was obtained as a complex copolymer, prepared by reacting the 1,3-dibromo-5-octyl-4H-thieno[3,4-c]pyrrole-4,6(5H)-dione (TPD) with an equimolar mixture of the (4,4′-didodecyl-[2,2′-bithiophene]-5,5′-diyl)bis(trimethylstannane) (bithiophene, BTh) and 4,4-dioctyl-2,6-bis(trimethylstannyl)-4H-silolo[3,2-b:4,5-b’]dithiophene (Dithienosilole, DTS) [174]. The CsPbI_2_Br perovskite was treated with 1% of lead propionate to passivate the lead surface traps. This kind of perovskite was used to build mesoporous devices in which the **Poly(DTSTPD-r-BThTPD)** gave a PCE of 14.58%.

Starting from the concept of the OMe-TATPyr 2D star-shaped small molecule, which obtained a PCE of 20.6% a polymer was derived, coded **poly-TATPyr** [175]. It was conceived as pyrene as a scaffold bearing four triphenylamines, through thiophene as a spacer. The final coupling to obtain the monomer was performed through a Suzuki reaction of the 1,3,6,8-tetra(5-bromothiophen-2-yl)pyrene with the 4-(diphenylamino)phenylboronic acid. The polymer **poly-TATPyr** was prepared from the monomer by electropolymerization, giving a n amorphous polymer. This kind of polymer showed low solubility, and this suggested using it for inverted architectures. Its low wettability towards the DMF was thought to suppress the heterogeneous nucleation and in turn, promoted the production of very large perovskite grains. The HOMO energy level was lying at −5.4 eV, very close to the perovskite valence band, −5.5 eV. Into a PSC with the ITO/HTM/(FAPbI_3_)_x_(MAPbBr_3_)_(1-x)_/PC_61_BM/BCP/Ag, the PCE of **poly-TATPyr** attained 16.5%, similar to **PTAA** (16.38%).

A polymer prepared by the Stille reaction on the basis of the isoindoloindole (IDID), **PIDF-BT**, to which bithiophene, was linked, attained interesting results. All these polymers were studied mainly to check the charge transfer with the dopant F4-TCNQ [176]. The dopant-free **PIDF-BT** obtained some good results about PCE, which was around 17.45% for PIDF-BT and was increased to 20.21% by F4-TCNQ doping. Unfortunately, no other data were shown on the dopant-free performances of the other polymeric materials.

Two polymers belonging to the poly(phenylene enylene) class were prepared, with pendant groups constitute by fluorene moieties decorated with two bis(4-methylphenyl)amines [177]. The synthetic path followed the transformation of the 2,7-bis(di-4-methylphenylamino)-9H-fluoren-9-one into the 9-(dibromomethylene)-*N*^2^,*N*^2^,*N*^7^,*N*^7^-tetra-p-tolyl-9H-fluorene-2,7-diamine. This intermediate was reacted with both 1,4-diethynylbenzene and 1,3-diethynylbenzene by the Sonogashira protocol to give the **PPE1** and **PPE2**. Those HTMs were used in inverted perovskites, where the **PPE2** film helped to grow larger and better-aligned perovskite crystals than **PPE1**. This gave an account for a better and outstanding PCE, 21.31% for **PPE2** (doped **PTAA** 21.56%) while **PPE1** performance was lower, 11.13%.

As evolution in the class of poly(phenylene enylene), the **PPY1** and **PPY2** polymers were prepared, embedding in the polymer skeleton a pyridine moiety to help to passivate the perovskite Pb surface traps [178]. The synthesis was accomplished by the Sonogashira method. The difference between the two polymers is related to the two pyridines involved in the reaction, the 2,6-diethynylpiridine (**PPY1**) and the 3,5-diethynylpiridine (**PPY2**). The **PPY1** HTM was shown to be an oligomer (2.2 kDa) and this can in part explain its lower photovoltaic results with respect to **PPY2**. The reason is probably due to the electron-withdrawing effect of the pyridine nitrogen which reduces the electron density to the ethynyl groups, also reducing their reactivity. On the contrary, **PPY2** was a polymer (7.6 kDa). In the (FA_0.92_MA_0.08_)_0.9_Cs_0.1_Pb(I_0.92_Br_0.08_)_3_ perovskite based PSCs, the two HTMs obtained a PCE of 15.08% and 22.41% for **PPY1** and **PPY2** respectively, with **PPY2** overcoming the **PTAA** by 1.4%.

Two polymers, **PDT-T** and **PDTT-T** were prepared by the Stille coupling of a benzo[1,2-c:4,5-c’]dithiophene-4, 8-dione (BDD) monomer and 4,9-dihydro-s-indaceno[1,2-b:5,6-b’]dithiophene (IDT) monomer for **PDT-T**, and 6,12-dihydro-dithieno[2,3-d:2’,3’-d’]-s-indaceno[1,2- b:5,6-b’]dithiophene monomer for **PDTT-T** [179]. The polymers were slightly soluble in THF and this fact demonstrated a proneness to π–π stacking. The HOMO energy levels of the two HTMs were at −5.45 and 5.44 eV for **PDT-T** and **PDTT-T**, respectively and the hole mobility was quite good and comparable between the two polymers. The thermal stability of these polymers was very good, over 400 °C. The polymers gave very good and smooth films that were used to build planar PSCs. The PCE was 18.63% for **PDT-T** and 19.02% for **PDTT-T**, also thanks to a fill factor approaching 80% for both polymers.

Thiazolothiazole-based HTMs, **P1**, **P2** and **P3** were prepared by Li and coworkers, who showed that the introduction of the thiazolothiazole moiety gave important results, showing excellent hole mobilities [180]. In order to avoid misunderstanding due to eponymous polymers already cited, here we will name these polymers as **TZ-P1**, **TZ-P2** and **TZ-P3**. The main building block was assembled with bithiophene (**TZ-P1**), thieno[3,2-b]thiophene (**TZ-P2**), and dithieno[3,2-b:2′,3′-d]thiophene (**TZ-P3**). The HOMO energy level for **TZ-P1**, **TZ-P2** and **TZ-P3** were very similar (−5.21 eV, −5.19 eV and -5.20 eV, respectively) with LUMO being −3.33, −3.36 and −3.36 eV. Thus, the difference of PCE among the three polymers (see below) could not be ascribed to a different level alignment. The XRD measurements showed that **TZ-P2** was more crystalline and this helped a better hole extraction from the perovskite, also confirmed by Time-Resolved Fluorescence measurements. On a mesoporous PSC containing a FA_0.81_MA_0.15_PbI_2.55_Br_0.45_ perovskite, **TZ-P1**, **TZ-P2** and **TZ-P3** gave 10.26%, 13.21% and 8.47% PCE respectively, with the best cell, showing 14.02% for **TZ-P2**. In the same conditions, **spiro-OMeTAD** gave a 13.02% PCE. Under N_2_, in a glovebox, the **TZ-P2** retained 80% of the initial PCE over 113 days, while for **spiro-OMeTAD** only 40% PCE was maintained over 97 days. Notably, **TZ-P2** maintained 97% of the pristine Fill Factor over 113 days, which demonstrated that the charge extraction ability was preserved.

**PEDOT-TMβCD** polymers were prepared and tested in perovskite solar cells [181]. They were prepared by polymerization of EDOT in the presence of 2,3,6-tri-O-methyl β-cyclodextrin (TMβCD) as a complexing agent. The TMβCD and EDOT were complexed and after they were polymerized by an oxidation reaction with FeCl_3_. The addition of a minimal quantity of the triphenylmethane-EDOT stopper was needed to obtain **PEDOT-TMβCD-P2**. These polymers were applied in MAPbI_3_ perovskite-based PSCs with a structure like glass/FTO/c-TiO_2_/m-TiO_2_/perovskite/HTM/Au). The HOMO of **PEDOT-TMβCD-P1** and **PEDOT-TMβCD-P2** were −4.32 and −4.20 eV, respectively. The polymers showed high melting point and crystallinity and powered a PCE as high as 5.54% for **PEDOT-TMβCD-P1**, 3.80% for **PEDOT-TMβCD-P2** and 6.27% for **spiro-OMeTAD**, without dopants. The reasons for the relatively low performance of PCE should be further studied, but as first evidence, the presence of a stopper in these “rotaxane” polymers. probably stabilizes the polymer inducing a better photovoltaic behaviour, with a 65% PCE increase for **PEDOT-TMβCD-P2** over **PEDOT-TMβCD-P1**, which was not capped with the stopper.

The development of PSC is also involving the use of fully inorganic perovskites to overcome the inherent instability of organo-inorganic perovskites [183]. In this respect, while already reaching remarkable efficiencies, the dopant-free HTMs used with fully inorganic perovskite are just a few.

PSC cells using a new lead-free (i.e., CsBi_3_I_10_) perovskite were built with three HTMs, **TQ1**, **P3HT** and **P3TI**[182]. The HOMO was −5.10 eV for **P3HT**, −5.69 eV for **P3TI** and −5.70 eV for **TQ1** and LUMO stands at −3.21 eV, −3.97 eV and −3.92 eV respectively. Albeit the PCE obtained in this case is relatively low, 0.36% for **P3HT**, 0.47% for **P3TI** and 0.51% for **TQ1**, this approach is really meaningful evidencing the issues toward a lead-free PSC technology. The recrystallization of the perovskite with tBP showed an increase in the perovskite crystals size which, however, did not fully cover the TiO_2_. On recrystallized perovskite, the **TQ1** was the only one HTM showing an increase of the PCE to 0.55% with the best cell reaching 0.77%[182]. These are the first results ever obtained on this new perovskite, which does not contain lead and should be less toxic and harmful.

## 4. Conclusions

The dopant-free polymers are relatively novel Hole Transporting Materials that appeared for the first time in 2013, showing high hole mobility without the use of any dopant that is partially responsible for the low stability of this technology. In this review, we collected all the dopant-free polymers reported in the literature implemented as HTMs in Perovskite Solar Cells. HTMs were considered not only from the photovoltaic efficiency point of view but we looked also at the structural features of the polymers. They were classified as homopolymers and donor–acceptor copolymers. Most of the highly efficient dopant-free polymers belong to the latter category. As a matter of fact, the continuous alternation of donor and acceptor moieties is one of the most important characteristics to achieve high performances along with a fully conjugated skeleton, in which the hole transfer is promoted. However, some well-performing polymers were not fully conjugated and sometimes they were homopolymers, e.g., polytriarylamines, or polyvinyl structures embodying pendants small molecule HTMs. Another critical point to obtain very efficient devices is the close contact among polymeric chains, which can help the hole transfer by hopping mechanism. It was clearly shown that polymers that can arrange face-on on the perovskite surface can show very high hole mobility in the vertical direction, improving the PSC efficiency, while often those that show an edge-on packing show lower performances. The polymers were studied from their structural point of view, classifying them based on the few largely occurring different donors or acceptor groups, such as diketopyrrolopyrrole (DPP), benzodithiophene (BDT), Benzothiadiazole (BTZ), Carbazole (Cbz). It was shown that minimal structural differences can cause a reasonably large increase of the photovoltaic efficiency in PSC. In the case of DPP core, for example, the simple insertion of a thienothiophene moiety increased the PCE from 7.1% of DPP-P1 to 17.8% of PDPPT-TT, also named DPP-DTT). Moreover, DPP-P4 (PCE 9.3%) that differs from PDPPT-TT only for the lateral chain length evidenced that the main conjugated chain analogy is not enough to obtain the same efficiency but that the lateral chains have great importance too. The same concept applies in the case of the BDT donor core in which very simple thiophene was used as a spacer among BDT groups, showing that it was sufficient to increase the planarity of the polymer and to improve packing and close contact, to achieve outstanding performances (19.6% for P2-T and 20.3% for P3-T-F). Very remarkably, most of the complex acceptor groups coupled with BDT did not achieve the results obtained by using a simple thiophene spacer. A further interesting aspect was that some HTMs which did not give high performances with a specific perovskite (DPP-P4), e.g., the classical MAPbI_3_, can greatly improve their performance in presence of other perovskites with which they are showing a better energy level alignment. These point toward the need for thoughtful design of HTMs to be employed with specific PSKs. Last but not least, the insertion of structural elements that can improve the contact between the perovskite and the HTM was found to be essential in improving the device’s efficiency. As an example, a few polymers were functionalized with methoxytriethylenoxy chains, which were found to interact with the lead cations exposed at the perovskite surface, strongly reducing the trap surface states and giving a better wetting and adhesion of the polymer onto the perovskite surface. This improved the contact and the hole transfer, giving outstanding efficiencies (19.8% for PTEG, 21.2% for Alkoxy-PTEG and 20.19% for DTB(3%DEG)). Finally, it was shown that all the different PSC structures reached efficiencies even higher than 20%. The mesoporous PSCs reached 20.3% with P3-T-F (and a certified PCE of 20.10% with DTB(3%DEG)), the planar PSCs 21.2% with Alkoxy-PTEG and the inverted PSCs 21.68% with PFDT-2F-COOH. Additionally, other groups were shown to act as passivating groups/agents for the perovskite Pb surface traps, such as (but not exclusively!) the trialylamino pendant groups, as for PBDT-N0, PBDT-N5 and PBDT-N20. In the absence of the passivating group, PBDT-N0 showed a decent PCE of 12.4%, but when the passivating groups were introduced in the polymer, up to 20% for PBDT-N20, the PCE grew to 18.9% showing that almost every HTM structure should be engineered for trap passivation. Alternative approaches are now followed, such as the use of polyectrolytes or passivating polymers directly during the perovskite preparation process, to improve the charge mobility throughout the perovskite material, which seems to be an element that limits the performance. This is also witnessed by several cases in which the surface traps passivation is performed by using very thin interlayers of passivant polyelectrolytesor conductive polymers, on which the HTM is finally deposited, improving its performance, e.g., the common P3HT, which increased its PCE from about 13% up to 19% by depositing first an interlayer of P3CT-BN. The same polymer attained a record PCE of 23% when deposited onto a classical perovskite capped with a thin layer of a 2D perovskite, which was passivating surface traps. In the last year, several polymers overcame the PCE barrier of 21% (21.31% for PPE2, 21.2% for Alkoxy-PTEG, 21.53% for 2DP-TPB, 21.68% for PFDT-2F-COOH) and some of them achieved PCE of 22% and more (22.0% for a DTB composite with CuSCN, and 22.41% for PPY2). The last case of PPY2 showed that a phenylene–vinylene polymer with a simple HTM as a pendant group can achieve outstanding results. All these cases show that there is plenty of space for playing with organic chemistry to build more performant molecules. As an example, the 2D star-shaped concept, well applied with success in the small molecules HTM field was applied to the synthesis of a branched polymer, 2DP-TPB, which attained an excellent PCE (21.53%) which rose up to 22.17% with an optimized perovskite. These impressive results were obtained in just a few years and the urge for efficient and stable photovoltaic systems will push the research to obtain better efficiencies. As a final remark, we hope that this journey onto the dopant-free HTM structures can be more than just a collection of interesting data but can give some insights towards a fruitful innovation in HTM design.

## Figures and Tables

**Table 1:** Dopant-free homo-polymeric HTM performances. (see legend for details).

HTM	PSK	Cell ^b^	PCE (%)	Std PCE (%)	V_oc_ (mV)	J_sc_ (mA cm^−2^)	FF (%)	HOMO (eV)	B_g_ (eV)	HM (cm^2^ V^−1^ S^−1^)	Stab. (h)	Ref.
**PTAA ^a^** (commercial)	((FAPbI_3_)_0.95_ (MAPBr_3_)_0.5_	M	22.6 ^a^	-	1.119	25.0	81.7	-	-	-	>93%, 13 months, NE, (amb. air, rt)	[5]
**PTAA** (synth. published)	MAPbI_3_	p	17.6	**16.7 ^c^**	1.06	22.1	75.0	-	-	-	-	[80]
**CH_3_O-PTAA**	(FAPbI_3_)_0.85_ (MAPbBr_3_)_0.15_	M	18.79	**16.34 ^c^**	1.08	23.25	75.2	−5.17	2.95	5.59 × 10^−5^	97%, 1000 h (NE, 85% RH, 85 °C)	[81]
**P3HT**	MAPbI_3-x_Cl_x_	P	13.6	-	0.979	24.3	57.2	-	-	-	-	[82]
**P3HT**	CsPbI_2_Br	P	15.50	-	1.28	15.62	78.7	-	-	-	98%, 30d, (NE, 25% RH, rt)	[83]
**P3CT-BN**			19.05	**18.26 ^d^** 10.40	1.10	23.08	74.8	−5.22	2.27	1.90 × 10^−5^	-	[84]
**P3HT** **(P3CT-BN** **interlayer**)	CsFAMA	P	19.23	**19.24 ^d^**	1.10	23.52	74.2	-	-	-	80%, 2300 h (NE, 50% RH, rt)	[85]
**POWT**	FA_0.2_MA_0.8_PbI_2.9_Br_0.1_	I	17.50	**14.13 ^e^**	1.02	24.11	71.5	−5.48	2.05	5.74 × 10^−3^	85%, 54d, (NE, N_2_, rt)	[86]
**PEDOT-C6**	(FAPbI_3_)_0.85_ (MAPbBr_3_)_0.15_	M	12.1 (best) 11.4 (avg)	-	0.93	22.4	58.0	−5.17	1.67	1.07 × 10^−4^	-	[87]
**PEDOT-C10**	(FAPbI_3_)_0.85_ (MAPbBr_3_)_0.15_	M	16.2 (best) 15.4(avg)	-	1.06	23.3	65.0	−5.17	1.66	1.24 × 10^−4^	75%, 120h, (NE, 80% RH, rt)	[87]
**PEDOT-C14**	(FAPbI_3_)_0.85_ (MAPbBr_3_)_0.15_	M	14.8 (best) 13.8 (avg)	-	10.7	20.8	67.0	−5.15	1.68	0.92 × 10^−4^	-	[87]
**PANI**	MAPbI_3_	M	7.34	-	0.78	14.48	65.0	−5.23	2.46	-	91%, 1000 h, NE, (amb. air, light, rt)	[88]
**PT**	MAPbI_3_	P	14.70	11.0 ^d^	0.95	21.2	72.0	−5.18	-	-	-	[89]
**PPN**	MAPbI_3_	P	12.80	11.0 ^d^	0.97	19.7	66.0	−5.26	-	-	-	[89]
**PPP**	MAPbI_3_	P	15.80	11.0 ^d^	1.02	21.0	71.0	−5.31	-	-	-	[89]
**PVCz-OMeDAD**	FA_0.85_ MA_0.15_Pb(I_0.85_Br_0.15_)_3_	M	16.09	9.62/**18.5 ^c^**	1.085	21.96	67.5	−5.24	2.75	3.44 × 10^−4^	-	[90]
**PVCz-OMeDAD**	(3BBA)_2.1_ MA_3.8_Pb_4.8_I_11.7_Cl_3.8_	I	14.76	**17.10 ^c^**	1.16	16.97	75.0	−5.24	2.69	1.53 × 10^−4^	-	[91]
**PVCz-OMeTPA**	(3BBA)_2.1_ MA_3.8_Pb_4.8_I_11.7_Cl_3.8_	I	17.22	**17.10 ^c^**	1.18	18.95	77.0	−5.38	2.82	4.34 × 10^−4^	-	[91]
**P1-** **PVCzOMeTAD**	Cs_0.05_FA_0.81_ MA_0.14_PbI_2.55_Br_0.45_	P	16.78	**20.4 ^c^**/11.21	1.11	21.58	69.5	−5.24	2.95	1.12 × 10^−4^	80%, 30d, (NE, 30% RH, rt)	[92]
**P2-** **PVCzOMeTAD**	Cs_0.05_FA_0.81_ MA_0.14_PbI_2.55_Br_0.45_	P	18.45	**20.4 ^c^**/11.21	1.13	22.34	72.7	−5.21	3.19	1.32 × 10^−4^	80%, 30d, (NE, 30% RH, rt)	[92]
**PVP-CZ**	Cs_0.05_(FA_0.17_MA_0.83_)_0.95_Pb(Br_0.17_I_0.83_)_3_	I	17.75 (best) 16.61 (avg)	18.85 ^c^	0.97	22.57	81.1	4.82	2.93	3.1 × 10^−5^	-	[93]
**CL1-2 (P1-2)**	Cs_0.05_(FA_0.85_MA_0.15_)_0.95_Pb(I_0.9_Br_0.1_)_3_	I	18.7	16.0 ^f^	1.10	22.6	80.0	−5.1	1.8	-	100%, 3000 h, (NE, N_2_, 85 °C)	[94]
**DH-MeO-FDPA**	MAPbI_3-x_Cl_x_	P	15.9 (best) 15.50 (avg)	13.7 ^d^	1.09	19.54	74.7	−5.04	2.48	5.0 × 10^−5^	-	[95]
**VB-MeO-FDPA**	MAPbI_3-x_Cl_x_	P	18.7 (best) 18.21 (avg)	13.7 ^d^	1.15	20.89	77.8	−5.18	2.27	3.0 × 10^−4^	85%, 200 h, (NE, N_2_, light, rt)	[95]
**VB-Me-FDPA**	MAPbI_3-x_Cl_x_	P	17.9 (best) 16.50 (avg)	13.7 ^d^	1.16	20.17	76.5	−5.20	2.60	1.4 × 10^−4^	-	[95]
**V1162**	Cs_0.05_(FA_0.83_MA_0.17_)_0.95_Pb(I_0.83_Br_0.17_)_3_	I	14.49	-	0.88	21.60	76.4	−5.26	-	-	-	[96]
**V1162 crosslinked**	Cs_0.05_(FA_0.83_MA_0.17_)_0.95_Pb(I_0.83_Br_0.17_)_3_	I	14.71	-	1.08	21.51	63.5	−5.11	-	-	-	[96]
**V1187**	Cs_0.05_(FA_0.83_MA_0.17_)_0.95_Pb(I_0.83_Br_0.17_)_3_	I	15.51	-	0.93	20.73	80.3	−5.26	-	-	-	[96]
**V1187 crosslinked**	Cs_0.05_(FA_0.83_MA_0.17_)_0.95_Pb(I_0.83_Br_0.17_)_3_	I	16.77	-	1.07	21.40	73.3	−5.11	-	-	-	[96]
**HTM-P1**	Cs_0.05_FA_0.81_MA_0.14_ PbI_2.55_Br_0.45_	M	16.8	-	1.07	24.3	60.5	−5.15	2.83	1.6 × 10^−4^	>80%, 30d, NE, (30%, RH rt)	[97]
**HTM-M1**	Cs_0.05_FA_0.81_MA_0.14_ PbI_2.55_Br_0.45_	M	9.1	-	0.84	20.5	48.2	−5.18	2.83	6.1 × 10^−5^	-	[97]

^a^ doped; ^b^ M: mesoporous, P: planar, I: inverted; ^c^ PTAA; ^d^ ref. std. spiro-OMeTAD; ^e^ ref. std. PEDOT:PSS; ^f^ polyTPD. PCEs for doped standards are reported in bold.

**Table 2:** Performances of dopant-free polymeric HTMs based on the DDP scaffold. (see legend for details).

HTM	PSK	Cell ^a^	PCE (%)	Std PCE (%)	V_oc_ (mV)	J_sc_ (mA cm^−2^)	FF %)	HOMO (eV)	B_g_ (eV)	HM (cm^2^ V^−1^ S^−1^)	Stab. (h)	Ref.
**PCBTDPP**	MAPbBr_3_	M	3.04	0.76 ^b^	1.16	4.47	59.0	−5.4	-	0.02	-	[77]
**PCBTDPP**	MAPbI_3_	M	5.5	6.48 ^c^	0.83	13.86	48.0	−5.4	-	0.02	5.55%, 900 h, NE, (amb. air, rt)	[77]
**PDPPT-TT** (**solv. vap. anneal.**)	MA_1-y_FA_y_PbI_3-x_Cl_x_	P	17.8	**16.5 ^c^**	1.07	22.8	72.0	-	-	-	26%, 72 h, NE, (amb. air, rt)	[100]
**DPP-P1**	MAPbI_3_	M	7.1	-	0.94	13.9	54.0	−5.35	1.35	8.8 × 10^−5^	-	[26]
**DPP-P2**	MAPbI_3_	M	7.3	-	0.94	11.5	67.0	−5.20	1.58	4.4 × 10^−2^		[26]
**DPP-P3**	MAPbI_3_	M	4.5	-	0.90	9.1	55.0	−5.25	1.40	2.7 × 10^−5^		[26]
**DPP-P4**	MAPbI_3_	M	9.1	-	0.95	13.1	74.0	−5.19	1.54	3.7 × 10^−2^		[26]
**DPP-P4**	Cs-Perovskite	M	14.2	-	1.01	19.7	72.0	−5.19	1.54	3.7 × 10^−2^		[26]
**DPP-P4**	Rb-Perovskite	M	16.3	-	1.05	20.6	75.0	−5.19	1.54	3.7 × 10^−2^	100%, 30d, NE, (dry air, rt)	[26]
**PDPP3T**	MAPbI_3_	M	12.3	**12.3 ^c^**	0.98	20.5	61.2	−5.30	1.56	-	39.4%, 172 h, NE, (20% RH, rt)	[101]
**PDPPDBTE** (**same as** **PDVT-10**)	MAPbI_3_	M	9.2 (doped)	**7.6 ^c^ 6.3 ^b^**	0.855	14.4	74.9	−5.4	1.28	0.32 2.77 ann.	91%, 1000 h, NE, (20% RH, rt)	[102,103]
**PDVT-10**	MAPbI_3_	M	13.35 (best) 12.97 (avg)	14.60 ^d^ (best) 11.28 (avg)	0.99	20.86	65.0	−5.28	1.68	8.2	100%, 20d, NE, (20% RH, rt),	[104]
**PBDTP-DTDPP**	Cs_0.05_(MA_0.17_ FA_0.83_)_0.95_Pb(I_0.83_Br_0.17_)_3_	P	14.73	**15.02 ^c^**	1.08	19.43	68.9	−5.43	1.39	-	-	[105]
**P25NH**	(MA_0.8_ FA_0.2_)Pb(I_0.93_Cl_0.07_)_3_	P	18.1	15.5 ^b^	0.998	22.18	81.9	−5.18	1.68	2.1 × 10^−2^	70%, 100h, (NE, 85% RH, rt)	[106]
**P5NH**	(MA_0.8_ FA_0.2_)Pb(I_0.93_Cl_0.07_)_3_	P	18.1	15.5 ^b^	1.04	20.95	83.1	−5.4	2.0	5.13 × 10^−2^	-	[107]
**2DP-TPB**	FA_0.85_MA_0.15_PbI_3_	P	21.53 (best) 20.53 (avg)	**21.98 ^c^**	1.16	24.13	76.9	−5.31	1.89	2.1 × 10^−4^	95%, 1000 h (NE, 30% RH, rt) 81%, 376 h, (NE, N_2_, 80 °C)	[108]
**2DP-TPB**	FA_0.85_MA_0.15_PbI_3_ (optimized with 4-fluorobenzamide hydrochloride)	P	22.17 (optimized perovskite)	**21.98 ^c^**	1.16	24.02	79.57	−5.31	1.89	2.1 × 10^−4^	-	[108]

^a^ M: mesoporous, P: planar. ^b^ ref. std. P3HT; ^c^ ref. std. spiro-OMeTAD; ^d^ ref. std. PTAA. PCEs for doped standards are reported in bold.

**Table 3:** Performances of dopant-free polymeric HTMs based on the benzodithiophene (BDT) scaffold. (see legend for details).

HTM	PSK	Cell ^a^	PCE (%)	Std PCE (%)	V_oc_ (mV)	J_sc_ (mA cm^−2^)	FF (%)	HOMO (eV)	B_g_ (eV)	HM (cm^2^ V^−1^ s^−1^)	Cond. (S cm^−1^)	Stab (h)	Ref.
**PTB1**	MAPbI_3-x_Cl_x_	M	6.0	**10.9 ^c^**	0.543	19.3	57.2	−4.90	-	4.5 × 10^−4^	-	-	[117]
**PTB7**	MAPbI_3-x_Cl_x_	M	7.5	**10.9 ^c^**	0.699	18.0	59.8	−5.15	-	5.8 × 10^−4^	-	-	[117]
**PTB7**	MAPbI_3_	M	6.92 (best) 6.30 (avg)	2.82 ^b^ (best) 2.01 (avg) 8.80 ^c^ (best) 7.79 (avg)	0.81	15.36	56.0	−5.20	-	-	-	20%, 112 h, NE, (55–70% RH, rt)	[98]
**PTB7**	(QD) α- CsPbI_3_	P	12.55 (best) 11.65 (avg)	9.82 ^b^ (best) 9.30 (avg) 8.20 ^c^ (best) 5.20 (avg)	1.27	12.39	80.0	−5.08	1.63	6.3 × 10^−4^	-	-	[122]
**PTB7-Th**	MAPbI_3_	M	3.68 (best) 3.48 (avg)	2.82 ^b^ (best) 2.01 (avg) 8.80 ^c^ (best) 7.79 (avg)	0.87	7.54	56.0	−5.12	-	-	-	Incr. 60%, 112 h, NE, (55–70% RH, rt)	[98]
**PTB7-Th**	(QD) α -CsPbI_3_	P	10.60	9.82 ^b^ (best) 9.30 (avg) **8.20 ^c^** (best) **5.20** (avg)	1.24	11.05	78.0	−5.15	1.59	1.7 × 10^−3^	-	-	[122]
**PBDT-N0**	(FAPbI_3_)_0.85_ (MAPbBr_3_)_0.15_	M	12.4 (best) 10.6 (avg)	19.6 ^c^ 10.1	1.02	20.4	60.0	−5.44	1.63	8.9 × 10^−5^	-	65%, 1400 h, (NE, 85% RH, 80 °C)	[119]
**PBDT-N5**	(FAPbI_3_)_0.85_ (MAPbBr_3_)_0.15_	M	15.5 (best) 13.2 (avg)	**19.6**^c^ 10.1	1.05	22.4	66.0	−5.45	1.52	1.2 × 10^−4^	-	75% 1400 h, (NE, 85% RH, 80 °C)	[119]
**PBDT-N20**	(FAPbI_3_)_0.85_ (MAPbBr_3_)_0.15_	M	18.9 (best) 17.0 (avg)	**19.6 ^c^** 10.1	1.08	23.4	75.0	−5.44	1.52	4.6 × 10^−4^	-	95% 1400 h, (NE, 85% RH, 80 °C)	[119]
**RCP-BTT**	Cs_0.05_(FA_0.85_ MA_0.15_)_0.95_Pb(I_0.85_Br_0.15_)_3_	M	8.45	**19.86^c^**	1.08	14.68	53.0	−5.28	1.64	2.3 × 10^−3^	-	>95, 500 h, (NE, 30% RH, rt) 35, 150 h, (E, 85% RH, 85 °C)	[121]
**PTB-BO**	MAPbI_3_	M	7.4 (best) 6.1 (avg)	7.0 ^c^/**11.6**	0.827	14.35	62.0	−5.25	1.93	5.30 × 10^−5^	-	Incr. 21%, 140 h, NE (desiccator, rt)	[123]
**PTB-DCB21**	MAPbI_3_	M	8.7 (best) 6.0 (avg)	7.0 ^c^/**11.6**	0.888	15.35	64.0	−5.22	1.91	5.01 × 10^−5^	-	Incr. 45%, 140 h, NE (desiccator, rt)	[123]
**PBDTTT-CP**	MAPbI_3_	P	9.95 (best) 9.32 (avg)	6.17 ^b^ (best) 5.47 (avg)	0.868	17.68	64.83	−5.12	1.77	2.4 × 10^−4^	-	-	[124]
	MAPbI_3_	M	6.64	4.24 ^b^	0.84	11.98	66.0	−5.24	1.76	6.68 × 10^−4^	-	-	[125]
**RCP**	MAPbI_3_	P	17.3	3.8/**15.3 ^c^**	1.08	21.9	75.0	−5.41	1.66	3.09 × 10^−3^	-	100%, 1400 h, NE, (75% RH, rt)	[126]
**P1**	MAPbI_3_	M	11.22	8.74/**18.28 ^c^**	1.03	16.12	67.68	−5.05	1.60	8.84 × 10^−3^	-	-	[127]
**P2**	MAPbI_3_	M	12.29	8.74/**18.28 ^c^**	1.01	16.84	72.02	−5.09	1.60	1.33 × 10^−3^	-	-	[127]
**P3**	MAPbI_3_	M	17.28	8.74/**18.28 ^c^**	1.09	20.74	75.78	−5.06	1.65	2.01 × 10^−3^	-	100%, 0d, NE, (amb. air, rt)	[127]
**asy-PBTBDT**	Cs-perovskite	M	18.3 (best) 17.4 (avg)	9.9/**18.5 ^c^**	1.11	22.4	73.2	−5.36	1.81	1.34 × 10^−3^	-	91%, 30d, NE, (50–75% RH, rt),	[128]
**PTEG**	Cs-perovskite	M	18.6	**17.9 ^c^**	1.09	22.7	75.4	−5.40	1.78	1.64 × 10^−3^	-	-	[115]
**PTEG**	Cs-perovskite	P	19.8	**17.0 ^c^**	1.14	22.5	77.0	−5.40	1.78	1.64 × 10^−3^	-	-	[115]
**Alkoxy-PTEG**	Cs_0.06_FA_0.78_ MA_0.16_Pb_0.94_I_2.4_Br_0.48_	P	21.2	**21.0 ^c^**	1.14	23.2	79.8	−5.31	1.75	4.06 × 10^−4^	-	88%, 30d, (NE, 40–50% RH, dark, rt)	[116]
**pBBTa-BDT1**	MAPbI_3_	P	7.0 (best) 5.05 (avg)	3.9/**15.2 ^c^**	0.77	18.1	50.6	−5.11	1.39	4.6 × 10^−5^	6.6 × 10^−6^	100%, 18 h, NE, (65% RH, 85 °C), 100%, E, (65% RH, 85 °C)	[129]
**pBBT-BDT2**	MAPbI_3_	P	14.5 (best) 12.05 (avg)	3.9/**15.2 ^c^**	0.95	20.3	75.2	−5.21	1.37	2.0 × 10^−3^	1.3 × 10^−5^	100%, 18 h, NE, (65% RH, 85 °C), 100%, E, (65% RH, 85 °C)	[129]
**P1-0F**	MAPbI_3_	M	9.80	8.74/**18.47 ^c^**	1.00	17.85	54.66	−5.41	1.82	2.51 × 10^−5^	-	>75%, 720 h, NE, (amb. air, rt)	[130]
**P2-2F**	MAPbI_3_	M	14.94	8.74/**18.47 ^c^**	1.04	20.17	70.65	−5.43	1.82	9.32 × 10^−5^	-	>75%, 720 h, NE, (amb. air, rt)	[130]
**P3-4F**	MAPbI_3_	M	10.35	8.74/**18.47 ^c^**	1.03	18.58	53.81	−5.50	1.82	4.06 × 10^−5^	-	>75%, 720 h, NE, (amb. air, rt)	[130]
**P-TT-TPD**	CsFAMA	M	17.10 (best) (16.96 (avg)	**21.5 ^c^**	1.04	21.86	74.25	−5.42	1.79	1.29 × 10^−4^	-	-	[131]
**K1**	MAPbI_3_	P	16.02 (best) 15.19 (avg)	-	1.06	19.22	78.0	−5.29	1.96	2.96 × 10^−5^	-	85%, 200 h, NE, (amb. air, rt)	[132]
**K2**	MAPbI_3_	P	3.85 (best) 2.9 (avg)	-	0.92	7.69	55.0	−5.35	1.48	2.55 × 10^−7^	-	-	[132]
**K3**	MAPbI_3_	P	12.50 (best) 11.98 (avg)	-	1.02	16.94	73.0	−5.40	1.97	1.41 × 10^−6^	-	-	[132]
**K4**	MAPbI_3_	P	6.45 (best) 5.84 (avg)	-	0.94	11.07	63.0	−5.31	1.53	9.67 × 10^−7^		-	[132]
**K5**	MAPbI_3_	P	14.65 (best) 13.83 (avg)	-	1.05	18.32	76.0	−5.40	1.92	5.68 × 10^−6^	-	-	[132]
**K6**	MAPbI_3_	P	13.77 (bes) 13.06 (avg)	-	1.04	17.75	75.0	−5.42	1.92	4.89 × 10^−6^	-	-	[132]
**R1**	MAPbI_3_	M	15.88	--	0.98	23.70	68.3	−5.42	1.60	3.7 × 10^−3^	-	100%, 500 h, NE (75% RH, rt)	[133]
**R2**	MAPbI_3_	M	13.57	-	0.88	23.10	66.2	−5.37	1.60	5.8 × 10^−4^		around 67%, 500 h, NE (75% RH, rt)	[133]
**PBT1-C**	CsFAMA	M	19.06 (best) 18.6 (avg)	14.96 ^b^ (best) 14.53 (avg)	1.05	22.37	81.22	−5.43	1.93	1.5 × 10^−3^	-	87%, 300 h NE (inert gas, rt)	[134]
**PBDB-T**	CsPbI_2_Br	P	14.20 (best) 13.56 (avg)		1.12	15.33	81.7	−5.28	1.80	3.07 × 10^−4^	-	-	[135]
**PBDB-T-2Cl**	CsPbI_2_Br	P	14.03 (best) 13.21 (avg)		1.21	14.19	77.2	−5.51	1.80	4.56 × 10^−4^	-	-	[135]
**PBDB-T-2F**	CsPbI_2_Br	P	14.87 (best) 14.12 (avg)		1.20	14.62	81.7	−5.47	1.80	5.68 × 10^−4^	-	-	[135]
**PBDB-T-Si**	CsPbI_2_Br	P	15.60 (best) 14.95 (avg)		1.20	15.28	82.5	−5.45	1.87	6.75 × 10^−4^	-	-	[135]
**P1-B**	MAPbI_3_	M	14.9 (best) 13.3 (avg)	**20.4 ^c^** (best) **19.7** (avg)	0.96	21.5	63.8	−5.16	2.27	3.0 × 10^−5^	-	-	[136]
**P2-T**	MAPbI_3_	M	19.6 (best) 19.2 (avg)	**20.4 ^c^** (best) **19.7** (avg)	1.07	22.8	77.6	−5.27	2.03	3.8 × 10^−4^	-	96%, 40d, NE (20–70% RH, rt)	[136]
**P3-T-F**	MAPbI_3_	M	20.3 (best) 19.7 (avg)	**20.4 ^c^** (best) **19.7** (avg)	1.09	22.8	78.5	−5.38	1.98	5.7 × 10^−4^	-	96%, 40d, NE (20–70% RH, rt)	[136]
**P1-Th-BDT-BO**	(FAPbI_3_)_0.85_ (MAPbBr_3_)_0.15_	P	18.30 (best) 17.76 (avg)	**19.35 ^c^** (best) **18.56** (avg)	1.06	22.39	75.0	−5.43	2.09	1.72 × 10^−3^	-	>89%, 96 h, NE (80% RH, rt)	[137]
**P2-OR-BDT-BO**	(FAPbI_3_)_0.85_ (MAPbBr_3_)_0.15_	P	7.57 (best) 7.55 (avg)	**19.35 ^c^** (best) **18.56** (avg)	0.81	21.75	42.9	−5.76	2.20	3.78 × 10^−4^	-	>89%, 96 h, NE (80% RH, rt)	[137]
**P3-OR-BDT-T**	(FAPbI_3_)_0.85_ (MAPbBr_3_)_0.15_	P	13.03 (best) 12.92 (avg)	**19.35 ^c^** (best) **18.56** (avg)	0.93	22.03	63.3	−5.30	2.13	2.54 × 10^−4^	-	>89%, 96 h, NE (80% RH, rt)	[137]
**PBDT[2F]T**	MAPbI_3_	P	17.52 (best) 16.55 (avg)	**20.20 ^c^ 15.59 ^d^**	1.06	22.64	72.60	−5.29	2.1	9.2 × 10^−6^	-	91%, 16d, NE (30% RH, 30 °C)	[138]
**PBDT(T)[2F]T**	MAPbI_3_	P	11.63 (best) 10.25 (avg)	**20.20 ^c^ 15.59 ^d^**	1.03	16.59	67.95	−5.22	2.0	-	-	-	[138]
**PBDT[2H]T**	MAPbI_3_	P	14.24 (best) 13.50 (avg)	**20.20 ^c^ 15.59 ^d^**	1.05	20.89	64.97	−5.03	2.1	2.2 × 10^−6^ [139]	-	-	[138]

^a^ M: mesoporous, P: planar. ^b^ ref. std. P3HT; ^c^ ref. std. spiro-OMeTAD; ^d^ ref. std. PTAA. PCEs for doped standards are reported in bold.

**Table 4:** Dopant-free benzothiadiazole and carbazole polymeric HTM performances. (see legend for details).

HTM	PSK	Cell ^a^	PCE (%)	Std PCE (%) ^b^	V_oc_ (mV)	J_sc_ (mA cm^−2^)	FF (%)	HOMO eV)	B_g_ (eV)	HM (cm^2^ V^−1^ S^−1^)	Stab. (h)	Ref.
**PCDTBT**	MAPbI_3_	P	16.5	-	1.05	21.9	72.0	−5.3	-	-	-	[99]
**PCDTBT**	MAPbI_3_	P	17.1 (best) 16.43 (avg)	**19.4 ^b^** (best) **18.81** (avg)	1.05	21.62	72.34	−5.35	-	-	>90%, 30d, E, (RH 40–70%, 25-35 °C),	[150]
**PCDTBT1**	MAPbI_3_	P	19.1 (best) 18.67 (avg)	**19.4 ^b^** (best) **18.81** (avg)	1.10	21.78	77.63	−5.38	-	-	>90%, 30d, E, (RH 40–70%, 25–35 °C),	[150]
**PCDTBT8**	MAPbI_3_	P	15.4 (best) 14.81 (avg)	**19.4 ^b^** (best) **18.81** (avg)	1.02	21.29	67.31	−5.40	-	-	25%, 30d, E, (RH 40–70%, 25–35 °C),	[150]
**BF-NMe_3_**	MAPbI_3_	I	14.92 (best) 14.65 (avg)	12.81 PEDOT: PSS	1.01	18.51	79.7	−5.40	1.88	-	>90% 960 h, (NE, 25% RH, rt)	[152]
**BF SO_3_**	MAPbI_3_	I	17.14 (best) 16.85 (avg)	12.81 PEDOT: PSS	1.05	19.92	82.1	−5.40	1.88	-	>90% 960 h, (NE, 25% RH, rt)	[152]
**BF-NH_3_**	MAPbI_3_	I	17.71 (best) 16.94 (avg)	12.81 PEDOT: PSS	1.05	20.10	84.04	−5.39	1.86	-	->90% 960 h, (NE, 25% RH, rt)	[152]
**TTB-TTQ**	MAPbI_3-x_Cl_x_	M	6,2	**11.5 ^c^** (best) **10.1** (avg)	0.94	11.8	55.4	−5.11	1.61	1.01 × 10^−4^	-	[153]
**BTTP**	MAPbI_3_	M	3.80	7.55 ^c^/**12.53 ^c^**	0.78	10.11	48.2	−5.40	2.2	-	-	[154]
**BTTP-CN**	MAPbI_3_	M	3.48	7.55 ^c^/**12.53 ^c^**	0.69	10.01	50.3	−5.25	2.13	-	-	[154]
**PVBT-SO_3_**	MAPbI_3_	I	18.4 (best) 17.57 (avg)	-	1.06	22.5	77.0	−5.27	1.72	-	-	[155]
**PVF2BT-SO_3_**	MAPbI_3_	I	13.6 (best) 13.2 (avg)	-	1.05	18.89	69.0	−5.40	1.78	-	-	[155]
**PVF4-SO_3_**	MAPbI_3_	I	7.3 (best) 6.69 (avg)	-	0.99	12.9	56.0	−5.66	2.16	-	-	[155]
**PDTIDTBT**	CsFAMA	M	17.9	**18.63 (spiro)**	1.09	22.5	79.0	−5.35	2.06	5.24 × 10^−4^		[156]
**PDTIDTBT**	CsFAMA (PMMA)	M	19.89	**19.28 (spiro)**	1.14	22.6	77.2	−5.35	2.06	5.24 × 10^−4^	>93%, 42d, (NE, 42% RH, rt)	[156]
**DTS**	MAPbI_3_(Cl)	I	17.27	16.92 ^c^	1.01	21.97	77.46	−5.01	1.67	8.83 × 10^−5^	-	[157]
**DTS**	MAPbI_3_(Cl)/ (PSBMA)	I	20.17	18.02 ^c^	1.10	22.44	81.67	−5.01	1.67	8.83 × 10^−5^	>83%, 400 h (NE, N_2_, 85 °C) >94%, 1500 h (NE, 20% RH, rt)	[157]
**CDT**	MAPbI_3_(Cl)	I	17.02	16.92 ^c^	0.98	22.23	78.17	−5.14	1.54	8.43 × 10^−5^	-	[157]
**CDT**	MAPbI_3_(Cl)/ (PSBMA)	I	20.05	18.02 ^c^	1.08	22.75	81.59	−5.14	1.54	8.43 × 10^−5^	>83%, 400 h (NE, N_2_, 85 °C) >94%, 1500 h (NE, 20% RH, rt)	[157]
**DTP**	MAPbI_3_(Cl)	I	14.87	16.92	1.10	21.44	63.05	−5.23	1.45	4.96 × 10^−5^	-	[157]
**DTP**	MAPbI_3_(Cl)/ (PSBMA)	I	17.60	18.02 ^c^	1.04	21.59	78.38	−5.23	1.45	4.96 × 10^−5^	>83%, 400 h (NE, N_2_, 85 °C) >94%, 1500 h (NE, 20% RH, rt)	[157]
**3,6PCzTPA**	(FAPbI_3_)_0.85_ (MAPbBr_3_)_0.15_	I	16.4	**18.1 ^c^**	0.96	21.93	77.9	−5.18	3.18	1.46 × 10^−5^	-	[158]
**2,7PCzTPA**	(FAPbI_3_)_0.85_ (MAPbBr_3_)_0.15_	I	17.1	**18.1 ^c^**	1.07	21.78	73.2	−5.23	2.86	1.49 × 10^−6^	-	[158]
**3,6-2,7-PCzTPA**	(FAPbI_3_)_0.85_ (MAPbBr_3_)_0.15_	I	18.4	**18.1 ^c^**	1.04	22.66	77.9	−5.22	3.00	1.74 × 10^−5^	-	[158]
**PC1**	MAPbI_3_	M	8.8	**20.3 spiro**	0.85	21.3	49.1	−5.37	2.37	4.2 × 10^−5^	-	[159]
**PC2**	MAPbI_3_	M	18.3	**20.3 spiro**	1.06	21.7	76.2	−5.17	2.13	7.3 × 10^−4^	-	[159]
**PC3**	MAPbI_3_	M	20.8	**20.3 spiro**	1.11	23.5	80.0	−5.16	2.09	8.1 × 10^−4^	100%, 1000 h (NE, light, rt)	[159]
**PDTPC-1**	CsFAMA	P	16.96	-	1.08	21.94	71.58	−5.11	1.60	3.98 × 10^−3^		[160]
**PDTPC-2**	CsFAMA	P	12.45	-	0.97	20.15	63.70	−4.97	1.57	2.94 × 10^−3^		[160]

^a^ M: mesoporous, P: planar. ^b^ ref. std. spiro-OMeTAD; ^c^ ref. std. PTAA. PCEs for doped standards are reported in bold.

**Table 5:** Dopant-free polymeric HTMs of various structures and their performances. (see legend for details).

HTM	PSK	Cell ^a^	PCE (%)	Std PCE (%)^b^	V_oc_ (mV)	J_sc_ (mA cm^−2^)	FF (%)	HOMO (eV)	B_g_ (eV)	HM (cm^2^ V^−1^ S^−1^)	Stab. (h)	Ref.
**PF8-TAA**	MAPbBr_3_	M	6.0	5.9 ^d^	1.36	6.3	70.0	−5.44		0.02	-	[162]
**PF8-TAA**	MAPbI_3_	M	4.6	16.2 ^d^	0.92	8.9	56.0	−5.44		0.02	-	[162]
**PIF8-TAA**	MAPbBr_3_	M	6.7	5.9 ^d^	1.40	6.1	79.0	−5.51		0.04	-	[162]
**PIF8-TAA**	MAPbI_3_	M	9.1	16.2 ^d^	1.04	19.0	46.0	−5.51		0.04	-	[162]
**PSQ1**	α- CsPbI_2_Br	P	13.6	**14.4 ^c^**	1.24	14.4	76.1	−5.21	1.79	1.09 × 10^−3^	-	[163]
**PSQ2**	α- CsPbI_2_Br	P	15.5	**14.4 ^c^**	1.27	15.4	79.0	−5.31	1.86	9.87 × 10^−3^	83%, 300 h, (light rt)	[163]
**P3-3,5-diOMe**	α- CsPbI_2_Br	P	11.6	**14.4 ^c^**	1.30	12.9	69.4	−5.27	2.47	1.45 × 10^−5^	-	[163]
**PBDTT**	Cs_0.05_FA_0.81_MA_0.14_ PbI_2.55_Br_0.45_	P	20.28	13.88 ^b^ **18.32 ^c^**	1.12	23.64	76.67	−5.31	1.87	7.10 × 10^−4^	80%, 720 h, NE (30% RH, rt)	[165]
**PBTTT**	Cs_0.05_FA_0.81_MA_0.14_ PbI_2.55_Br_0.45_	P	19.48	13.88 ^b^ **18.32 ^c^**	1.10	22.30	79.57	−5.24	1.89	6.99 × 10^−4^	80%, 720 h, NE (30% RH, rt)	[165]
**IDTB**	Cs_0.05_FA_0.81_MA_0.14_ PbI_2.55_Br_0.4_	P	19.38 (best) 16.39 (avg)	**18.22 ^b^**	1.107	23.06	75.9	−5.20	2.19	2.9 × 10^−5^	70%, 60d (NE, 30–40% RH, 30 °C)	[166]
**PFDT-COOH**	FA_1-x_MA_x_PbI_3_	I	20.64 (best) 19.65 (avg)	-	1.079	24.78	77.2	−5.22	2.18	-	80%, 720 h, NE (N_2_, 85 °C)	[167]
**PFDT-2F-COOH**	FA_1-x_MA_x_PbI_3_	I	21.68 (best) 20.51 (avg)	-	1.111	24.86	78.5	−5.32	2.3	-	80%, 720 h, NE (N_2_, 85 °C)	[167]
**MEH-PPV**	MAPbI_3_	M	9.65	6.74 ^b^/**13.38 ^c^**	0.88	17.36	63.0	−5.40	2.1			[168]
**DTB**	FA_0.85_MA_0.15_ PbI_2.55_Br_0.45_	M	19.68 (best) 19.19 (avg) 19.5 (cert)	**19.81 ^c^** (best) **19.38** (avg)	1.10	25.50	69.85	−4.96	2.14	1.0 × 10^−4^	-	[169]
**DTB/CuSCN**	FAPbI_3_)_0.92_ (MAPbBr_3_)_0.08_	P	22.0	-	1.15	24.31	78.6	−	-	0.014	90%, 1000 h, (NE, 60% RH, rt)	[170]
**DTB(0%DEG)**	Cs_0.05_FA_0.81_MA_0.14_ PbI_2.55_Br_0.45_	M	19.65 (best) 18.57 (avg)	-	1.10	25.01	71.48	−4.99	2.14	1.74 × 10^−4^	87%, NE (amb.air, rt)	[171]
**DTB(3%DEG)**	Cs_0.05_FA_0.81_MA_0.14_ PbI_2.55_Br_0.45_	M	20.19 (best) 19.22 (avg) 20.10 (cert)	-	1.14	23.64	75.08	−4.97	2.14	3.22 × 10^−4^	92%, NE (amb.air, rt)	[171]
**DTB(10%DEG)**	Cs_0.05_FA_0.81_MA_0.14_ PbI_2.55_Br_0.45_	M	17.82 (best) 16.78 (avg)	-	1.05	24.02	72.42	−4.94	2.14	2.02 × 10^−4^	87%, NE (amb.air, rt)	[171]
**PTA-P1**	MAPbI_3_	M	12.1 (best) 10.3 (avg)	**17.6 ^d^**, 4.9	1.04	21.6	54.0	−5.07	2.58	2.8 × 10^−−4^		[172]
**PTA-P2**	MAPbI_3_	M	1.3 (best) 0.97 (avg)	**17.6 ^d^** 4.9	0.83	11.3	14.0	−5.44	2.84	1.5 × 10^−−5^		[172]
**PTA-P3**	MAPbI_3_	M	9.1 (best) 8.2 (avg)	**17.6 ^d^** 4.9	0.97	20.6	45.0	−5.16	2.64	3.4 × 10^−−5^		[172]
**PTA-P4**	MAPbI_3_	M	11.1 (best) 10.3 (avg)	**17.6 ^d^** 4.9	1.04	21.6	50.0	−5.21	2.26	2.6 × 10^−−4^		[172]
**PTPADT-SO3Na**	MAPbI_3_	I	18.85	**13.86 ^e^**	1.08	21.26	79.2	−5.18	2.37	-	81%, 500 h (NE, 50% RH, rt) 79%, 200 h (NE, 50% RH, 80 °C)	[173]
**Poly** **(DTSTPD-r-** **BThTPD)**	CsPbI_2_Br:1%Pb (CH_3_CH_2_COO)_2_	M	14.58	**11.14 ^b^**	1.15	15.66	81.0	−5.44	1.70	1.5 × 10^−3^	-	[174]
**poly-TATPyr**	(FAPbI_3_)_x_ (MAPbBr_3_)_(1-x)_	I	16.5 (best) 15.5 (avg)	16.3 ^d^	0.998	23.3	71.0	−5.4	-	1.0 × 10^−5^	91%, 1000 h (NE, dark, rt)	[175]
**PIDF-BT**			17.45									[176]
**PPE1**	(FAPbI_3_)_0.83_ (MAPbBr_3_)_0.17_	I	11.13 (best) 9.50 (avg)	**21.56 ^d^**	1.03	15.53	70.0	−5.08	2.45	2.2 × 10^−6^	-	[177]
**PPE2**	(FAPbI_3_)_0.83_ (MAPbBr_3_)_0.17_	I	19.33 (best) 18.10(avg)	**21.56 ^d^**	1.07	22.84	79	−5.06	2.70	1.9 × 10^−6^	-	[177]
**PPE2** **(PSK passivation** **with PEAI)**	(FAPbI_3_)_0.83_(MAPbBr_3_)_0.17_	I	21.31 best 19.77 (avg)	**21.56 ^d^**	1.18	22.30	81	−5.06	2.70	1.9 × 10^−6^	-	[177]
**PPY1**	(FA_0.92_MA_0.08_)_0.9_Cs_0.1_Pb(I_0.92_Br_0.08_)_3_	I	15.08	**20.98 ^d^**	0.92	22.15	74.0	−4.96	2.67	1.30 × 10^−3^	-	[178]
**PPY2**	(FA_0.92_MA_0.08_)_0.9_Cs_0.1_Pb(I_0.92_Br_0.08_)_3_	I	22.41	20.98 ^d^	1.16	23.56	82.0	−5.17	2.48	1.90 × 10^−3^	>97%, 500h (NE, light, rt9	[178]
**PDT-T**		P	18.69	13.69 ^b^	1.13	21.36	77.63	−5.45	1.88	6.29 × 10^−4^	>90%, 100d, (E, 30% RH, rt)	[179]
**PDTT-T**		P	19.02	13.69 ^b^	1.14	21.68	76.79	−5.44	1.89	7.41 × 10^−4^	>90%, 100d, (E, 30% RH, rt)	[179]
**TZ-P1**	FA_0.81_MA_0.15_PbI_2.5_Br_0.45_	M	10.26	13.02 ^c^	0.94	17.62	62.0	−5.21	1.88	-	-	[180]
**TZ-P2**	FA_0.81_MA_0.15_PbI_2.5_Br_0.45_	M	13.21	13.02 ^c^	1.02	19.87	65.0	−5.19	1.83	-	80%, 113d, NE, (N_2_, rt)	[180]
**TZ-P3**	FA_0.81_MA_0.15_PbI_2.5_Br_0.45_	M	8.47	13.02 ^c^	0.90	16.47	57.0	−5.20	1.84	-	-	[180]
**PEDOT-TMbCD-P1**	MAPbI_3_	M	5.54 (best) 3.64 (avg)	6.27 ^c^/**14.41 ^c^**	0.74	19.51	38.0	−4.20	2.60	-	-	[181]
**PEDOT-TMbCD-P2**	MAPbI_3_	M	3.80 (best) 2.02 (avg)	6.27 ^c^/**14.41 ^c^**	0.60	13.55	47.0	−4.32	2.20	-	-	[181]
**TQ1**	CsBi_3_I_10_	M	0.51	0.36 ^b^/0.003 ^c^	0.48	2.9	37.0	−5.70	1.78	1.6 × 10^−3^		[182]
**P3TI**	CsBi_3_I_10_	M	0.47	0.36 ^b^/0.003 ^c^	0.47	2.6	38.0	−5.69	1.72	1.8 × 10^−3^		[182]

^a^ M: mesoporous, P: planar. ^b^ ref. std. P3HT; ^c^ ref. std. spiro-OMeTAD; ^d^ ref. std. PTAA; ^e^ ref. std. PEDOT:PSS. PCEs for doped standards are reported in bold. 3BBA: 3-bromobenzylammonium (quasi-2D perovskite). PEAI: Phenylethylammonium iodide. PSBMA: poly(sulfobetaine methacrylate). PMMA: poly(methyl metacrylate).

## Abbreviations

AgTFSI	Silver bis(TriFluoromethane-Sulfonyl)Imide
ALD-TiO_2_	Atomic Layer Deposited Titanium dioxide thin film
BCF	Tris(pentaflurophenyl)borane
BDT	Benzodithiophene
BG or B_g_	Band-Gap
BIPV	Building-integrated photovoltaics
BTZ	Benzothiadiazole
CB	Conduction Band
CBz	Carbazole
CsPbI_3_	Cesium Lead Iodide Perovskite
CsFAMA	Cesium FormAmidinium/MethylAmmonium Perovskite
c-TiO_2_	Compact Titanium Dioxide
CV	Cyclic Voltammetry
D-A	Donor–Acceptor
D-π-A	Donor–π-conjugated bridge–Acceptor
DFT	Density Functional Theory
DSC or DSSCs	Dye-Sensitized Solar Cells
ETL	Electron Transporting Layer
ETM	Electron Transporting Material
FA	Formamidinium
FAMA	Formamidinium methylammonium
FAPbI_3_	FormAmidinium Lead triiodide Perovskite
FF	Fill Factor
FTO	Fluorine doped Tin Oxide
GIWAXS	Grazing Incidence Wide Angle X-Ray Scattering
HM	Hole Mobility
HOMO	Highest Occupied Molecular Orbital
HTL	Hole Transporting Layer
HTM	Hole Transporting Material
IDT	Indacenothiophene
IDTT	Indacenodithieno[3,2-b]thiophene T
J_sc_	Short Circuit Current Density
LiTFSI	Lithium bis(TriFluoromethylSulfonyl)Imide
LUMO	Lowest Unoccupied Molecular Orbital
M_n_	Number Average Molecular Weight
M_w_	Weight Average Molecular Weight
MA	Methylammonium
MAPbBr_3_	MethylAmmonium Lead Bromide Perovskite
MAPbI_3_	MethylAmmonium Lead Iodide Perovskite
MAPbI_3-x_Cl_x_	MethylAmmonium Lead Iodide/Chloride
m-TiO_2_	Mesoporous titanium dioxide
NE	Non-Encapsulated devices
NMR	Nuclear Magnetic Resonance
OFET	Organic thin-film transistor
OLED	Organic Light Emitting Diode
OPV	Organic PhotoVoltaics
P	Maximum Power
P_in_	Incident Power at Constant Flux of Photons
P3HT	Poly(3-hexyl)thiophene
PANI	Polyaniline
PCE	Power Conversion Efficiency
PDI	Polydispersity index
PEDOT:PSS	Poly(3,4-ethylenedioxythiophene) polystyrene sulfonate
PFN	Poly[(9,9-bis(3′-(N,N-dimethylamino)propyl)-2,7-fluorene)-alt-2,7-(9,9-dioctylfluorene)]
PPN	Poly(4,4′-bis(N-carbazolyl)-1,10-biphenyl)
PPP	Polyphenylene
PSC	Perovskite Solar Cell
PT	Polythiophene
PTAA	Poly TriArylAmine
QD	Quantum Dot
RH	Relative Humidity
Spiro-OMeTAD	2,2′,7,7′-Tetrakis[N,N-di(4-methoxyphenyl)amino]-9,9′-spirobifluorene
tBP	4-Tert-ButylPyridine
V_oc_	Open Circuit Voltage
VB	Valence Band
